# Gamma band functional connectivity reduction in patients with amnestic mild cognitive impairment and epileptiform activity

**DOI:** 10.1093/braincomms/fcac012

**Published:** 2022-02-03

**Authors:** Pablo Cuesta, Manuela Ochoa-Urrea, Michael Funke, Omar Hasan, Ping Zhu, Alberto Marcos, Maria Eugenia López, Paul E. Schulz, Samden Lhatoo, Dimitrios Pantazis, John C. Mosher, Fernando Maestu

**Affiliations:** 1 Department of Radiology, Rehabilitation and Physiotherapy, Complutense University of Madrid, Madrid, Spain; 2 Department of Neurology, McGovern Medical School, The University of Texas Health Science Center at Houston, Houston, TX, USA; 3 Texas Institute for Restorative Neurotechnologies, University of Texas Health Science Center at Houston, Houston, TX, USA; 4 Department of Pediatrics, McGovern Medical School, The University of Texas Health Science Center at Houston, Houston, TX, USA; 5 Vivian L. Smith Department of Neurosurgery, McGovern Medical School, The University of Texas Health Science Center at Houston, Houston, TX, USA; 6 Neurology Department, Hospital Clinico San Carlos and Instituto de Investigación Sanitaria del Hospital Clínico San Carlos, Madrid, Spain; 7 Department of Experimental Psychology, Cognitive Processes and Speech Therapy, Complutense University of Madrid, Madrid, Spain; 8 McGovern Institute for Brain Research, Massachusetts Institute of Technology, Cambridge, USA

**Keywords:** MCI, epileptiform activity, functional connectivity, gamma

## Abstract

There is growing evidence for neuronal hyperexcitability in Alzheimer’s disease. Hyperexcitability is associated with an increase in epileptiform activity and the disruption of inhibitory activity of interneurons. Interneurons fire at a high rate and are frequently associated with high-frequency oscillations in the gamma frequency band (30–150 Hz). It is unclear how hyperexcitability affects the organization of functional brain networks. A sample of 63 amnestic mild cognitive impairment patients underwent a magnetoencephalography resting-state recording with eyes closed. Twenty (31.75%) mild cognitive impairment patients had epileptiform activity. A cluster-based analysis of the magnetoencephalography functional connectivity revealed a region within the right temporal cortex whose global connectivity in the gamma frequency band was significantly reduced in patients with epileptiform activity relative to those without epileptiform activity. A subsequent seed-based analysis showed that this was largely due to weaker gamma band connectivity of this region with ipsilateral frontal and medial regions, and the upper precuneus area. In addition, this reduced functional connectivity was associated with higher grey matter atrophy across several cortical regions in the patients with epileptiform activity. These functional network disruptions and changes in brain physiology and morphology have important clinical implications as they may contribute to cognitive decline in mild cognitive impairment and Alzheimer’s disease.

## Teaser

Cuesta *et al*. report that epileptiform activity in magnetoencephalography data of patients with mild cognitive impairment is linked to reduced gamma band functional connectivity of the right temporal cortex with the rest of the brain. This reduced connectivity was associated with grey matter atrophy across several cortical regions.

## Graphical Abstract

**Graphical Abstract:**
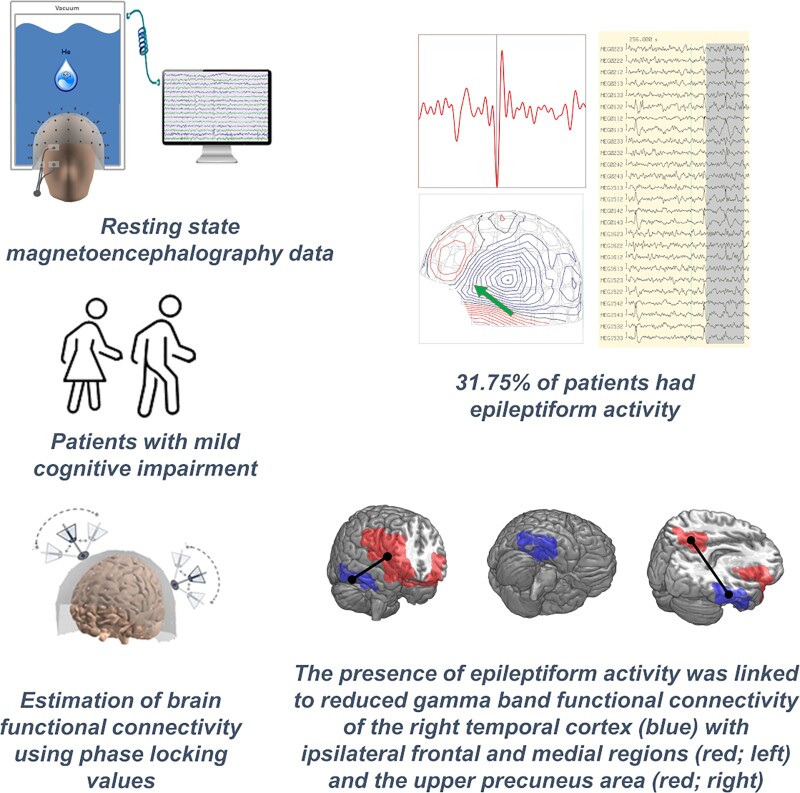

## Introduction

Cognitive decline in the development of Alzheimer’s disease has been associated with progressive brain atrophy and the accumulation of hyperphosphorylated tau and amyloid proteins.^1^ A functional neuronal network is supported by a fine balance between neuronal excitation and inhibition (E/I) and a disruption in this balance may lead to alterations in network organization. Additionally, network disruptions can affect signal transmission and a breakdown of interareal communication.

In the last decade, there has been growing evidence for hyperexcitability in Alzheimer’s disease patients. Compared with the general population, Alzheimer’s disease patients are 8–10 times more likely to develop spontaneous seizures.^2,3^ Cortical hyperexcitability in Alzheimer’s disease patients could reflect a disruption of the E/I balance. Animal models of Alzheimer’s disease have shown increased neuronal firing in the vicinity of amyloid plaques.^4^ Studies in humans have demonstrated that amyloid toxicity causes a loss of inhibitory terminals,^5^ and amyloidosis (lower amyloid-β levels in cerebrospinal fluid) is associated with the existence of late-onset epilepsy of unknown aetiology in mild cognitive impairment (MCI) patients.^6^ Finally, increased epileptiform activity (EA) has been found in brain regions typically affected by the neuropathology of Alzheimer’s disease.^7–10^

Hyperexcitability could lead to increased neuronal synchronization and a dysfunctional organization of the profiles of brain activity shown at multiple frequency bands with EEG and magnetoencephalography (MEG). Increased phase synchrony between the anterior and posterior regions has been found in mid-adult humans with amyloid deposition^11^ and in relatives of Alzheimer’s disease patients,^12^ all at preclinical stages. Furthermore, this neurophysiological signature was also found in elders with subjective cognitive decline^13^ and in MCI patients who later progressed to dementia.^14,15^

Palop and Mucke^16^ noted a link between cognitive impairment and disruption of interneuron inhibitory activity in a comprehensive review in 2016. In addition, the firing of interneurons is more prominent and synchronized with high-frequency oscillations, such as those in the gamma band (30–150 Hz). The gamma band has been frequently associated with local and long-distance communication^17^ and with memory function by predicting those items that will successfully be recalled later.^18^ A reduction of the gamma band power has been associated with the appearance of epileptic discharges in an animal model of Alzheimer’s disease.^19^ Furthermore, patients with epilepsy tend to show higher gamma band activity during successful encoding of words.^20^ Thus, extensive evidence now directly links the gamma band, memory formation and interneuron modulatory activity.

The goal of this study was to test whether hyperexcitability, in the form of EA, found in MCI patients induces alterations of crucial oscillatory activity associated with memory formation. We hypothesized that there would be greater network dysfunction in MCI with EA.

## Materials and methods

### Subjects

Sixty-three amnestic MCI patients were recruited from the Hospital Universitario San Carlos (Madrid, Spain).^21^ All were native Spanish speakers and right-handed. The MCI diagnosis was established according to the NIA-AA clinical criteria,^22^ which include (i) self- or informant-reported cognitive complaints, (ii) objective evidence of impairment in one or more cognitive domains, (iii) preserved independence in functional abilities and (iv) not demented.^23^ For more information about the diagnostic criteria for MCI see López *et al*.^24^ None of the participants exhibited a history of psychiatric or neurological disorders other than MCI. General inclusion criteria were as follows: age between 60 and 90 years, a modified Hachinski score ≤4, a short form Geriatric Depression Scale score ≤5 and a T_1_/T_2_-weighted MRI within 54 weeks before the MEG recordings (on average, the time period between the MEG and MRI recordings was 3 months) without an indication of infection, infarction or focal lesions (rated by two independent experienced radiologists^25^). In addition, we advised subjects to avoid medications that could affect MEG activity, such as benzodiazepines, for 48 h before recordings. All participants provided written, informed consent. The Institutional Review Board Ethics Committee at Hospital Universitario San Carlos approved the study protocol, and the procedure was performed following the Helsinki Declaration and National and European Union regulations.

### MRI acquisition and volumetric analyses

T_1_-weighted MRI images from each participant were acquired with a General Electric 1.5T MRI scanner using a high-resolution antenna and a homogenization PURE filter (Fast Spoiled Gradient Echo sequence, TR/TE/TI = 11.2/4.2/450 ms; flip angle 12°; 1 mm slice thickness, 256 × 256 matrix and FOV 25 cm). The resulting images were processed using Freesurfer software (v. 5.1.0) and its specialized tool for automated cortical parcellation and subcortical segmentation.^26^

### MEG recordings and interpretation

**Figure 1:**
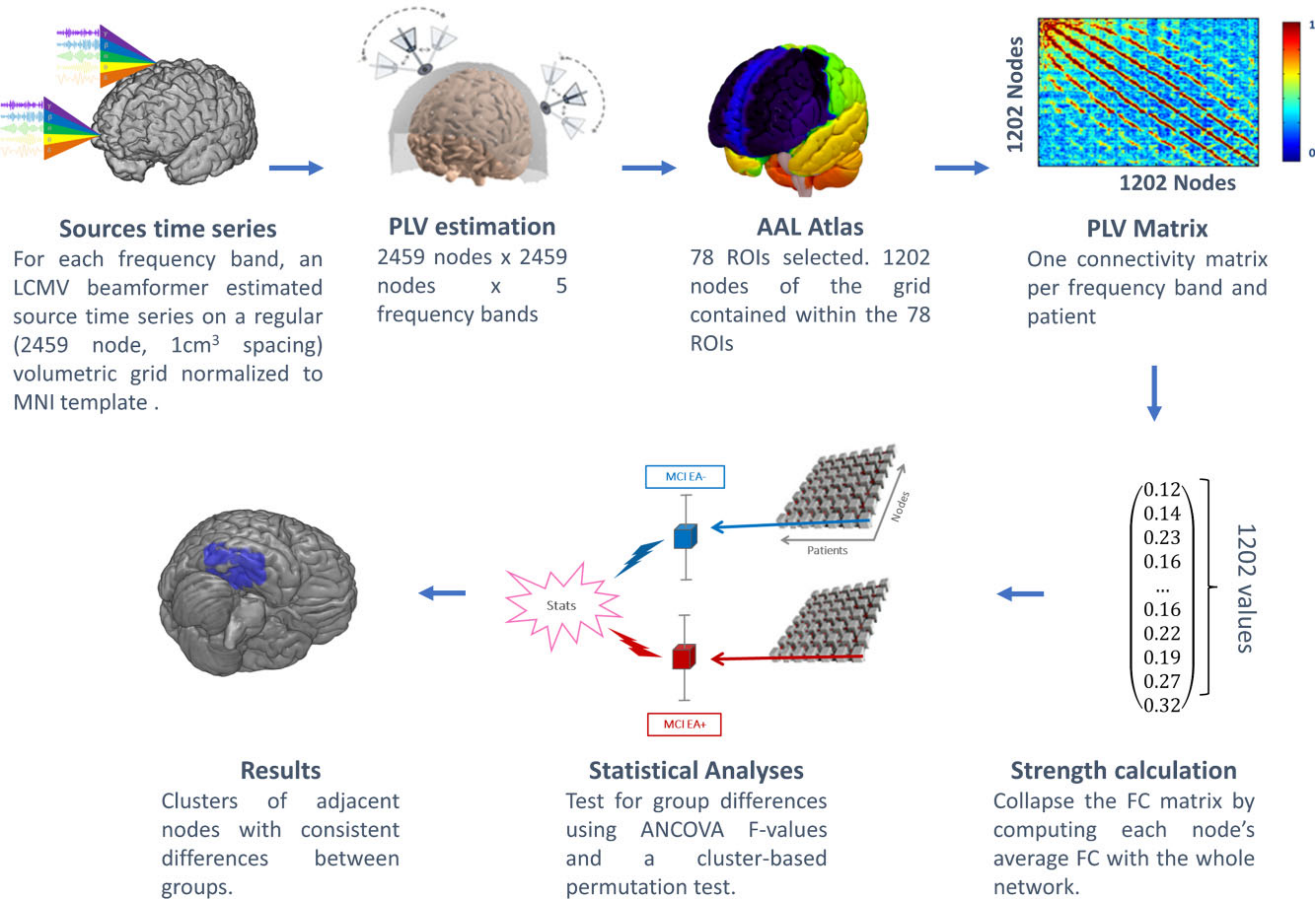
**Schematic diagram of the processing pipeline**. For each frequency band, an LCMV beamformer estimated source time series in a regular volumetric grid of nodes. Phase locking values (PLV) were then estimated between every pair of nodes contained within one of 78 ROIs from the AAL atlas. These values were used to compute the normalized strength of each node, defined as the sum of its PLV with the rest of the nodes, divided by the number of connected nodes. Last, the strength values were subjected to statistical analyses using ANCOVA and a cluster-based permutation test to identify clusters of adjacent nodes with significant functional connectivity differences between the MCI EA+ and MCI EA− groups.

A scheme of the methodological pipeline can be seen in Fig. 1. MEG signals were acquired using a whole-head Elekta-Neuromag MEG system with 306 channels (Elekta AB, Stockholm, Sweden) at the Center for Biomedical Technology (Madrid, Spain). Data were collected at a sampling frequency of 1000 Hz and online band-pass filtered between 0.1 and 330 Hz. The MEG protocol consisted of 5-min resting-state eyes closed, 5-min resting-state eyes open and 10-min of passive face viewing whilst sitting comfortably inside a magnetically shielded room. For functional connectivity analysis, we used the 5 min of resting-state eyes closed recordings. For EA screening, we visually inspected all three recordings comprising ∼20 min. Participants were asked to stay awake and to minimize their body movements. Each participant’s head shape was defined relative to three anatomical locations (nasion and bilateral preauricular points) using a 3D digitizer (Fastrak, Polhemus, VT, USA) and the head motion was tracked through four head-position indicator (HPI) coils attached to the scalp. These HPI coils continuously monitored the subjects’ head movements, whilst eye movements were monitored by a vertical electrooculogram (EOG) assembly composed of a pair of bipolar electrodes. Raw data were first processed with Maxfilter software [v. 2.2, temporal signal-space separation (tSSS), correlation threshold = 0.9, time window = 10 s] to remove external noise using the temporal extension of the signal-space separation method with movement compensation.^27^ Data underwent automatic artefact selection using FieldTrip,^28^ and a MEG expert confirmed the findings. After the artefact’s removal, we applied second-order, blind identification (SOBI)^29^ to remove artefacts from ECG, EOG and other noise-related interferences. Only the magnetometers’ data were used for subsequent analysis since the sensor-space data are highly redundant after Maxfilter processing.^30^ The remaining artefact-free data were partitioned into 4 s segments (epochs). Only those recordings with at least 20 clean segments (80 s of brain activity) were included in subsequent analyses. Prior to source estimation, the MEG time courses were filtered into theta (4.1–7.9 Hz), alpha (8.1–11.9 Hz), beta (12.1–29.9 Hz) and gamma (30.1–45.0 Hz) frequency bands with a 1500 order finite impulse response filter using a Hamming window and a two-pass filtering procedure.

**Figure 2:**
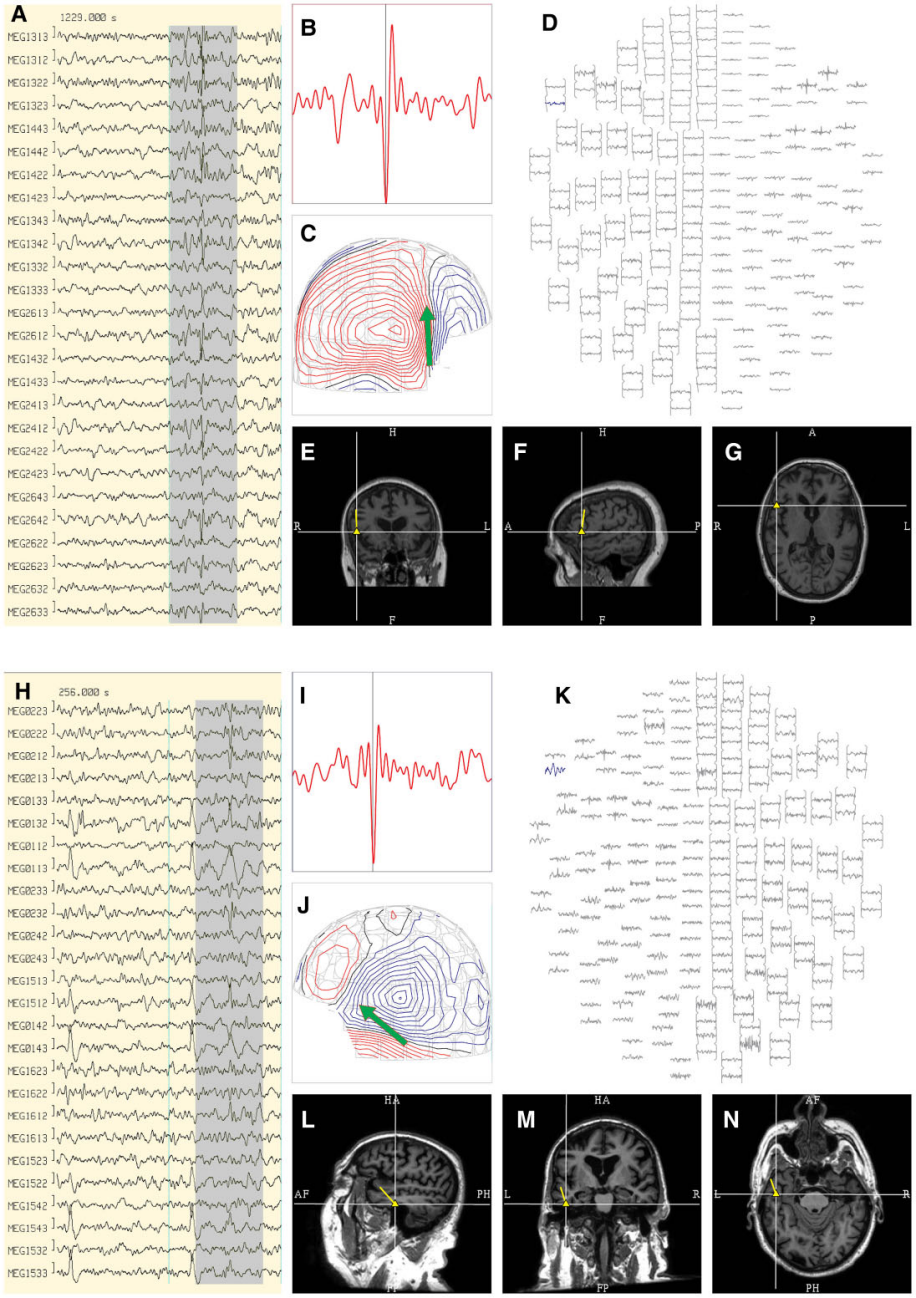
**Epileptiform activity**. Upper panel: (**A**) An example of an individual MEG discharge in the right frontal area (radiological convention). (**B**) Selected MEG channel and time instance of the magnetic field distribution (**C**) in sensor space with the projected source estimate (green arrow). The MEG channel plot (**D**) of the selected time interval as shown in **A** shows the planar gradiometer channel (red square and arrow) with the earliest peak time. Figures (**E–G**) represent the dipole location (yellow triangle) and its orientation (yellow tail) arising from the frontal opercular region in the coronal (**E**), sagittal (**F**) and axial (**G**) views. Lower panel: (**H**) An example of an individual MEG discharge in the left temporal area (neurological convention). (**I**) Selected MEG channel and time instance of the magnetic field distribution (**J**) in sensor space with the projected source estimate (green arrow). The MEG channel plot (**K**) of the selected time interval as shown in **H** shows the planar gradiometer channel (orange square and arrow) with the earliest peak time. Figures (**L–N**) represent the dipole location (yellow triangle) and its orientation (yellow tail) originating from anterior basal temporal structures in the sagittal (**L**), coronal (**M**) and axial (**N**) views.

A MEG expert (MEF) and a neurologist trained in MEG reading (MOU) screened MEG signals for EA (see Fig. 2). Both reviewers inspected the data together and decisions on the presence of EA discharges were taken by consensus. In case of disagreement, resolution relied on the opinion of the most experienced neurophysiologist (MEF). No additional opinion from a third reader was obtained. Localization of the spikes was obtained using single dipole modelling, which is the accepted approach in clinical MEG (see CPG 1).^31^ Raw data were reviewed after Maxfilter processing. We defined EA as a transient signal that was clearly distinguished from background activity, and with a pointed peak component.^32,33^ In order to be valid, only statistically significant sources (reduced χ^2^ ≥1 and ≤2, confidence volume <1000 mm^3^, source strength 100–500 nA, goodness of fit >75%) were accepted. Source localization of the dipoles was performed in the DANA Elekta Neuromag Software (Elekta AB, Stockholm, Sweden, now MEGIN, Helsinki, Finland) superimposing the dipoles on the patient’s structural MRI. Depending on the presence or absence of EA, patients were divided into two groups: MCI EA+ and MCI EA−, comprising patients with and without EA, respectively.

### Source reconstruction and connectivity analysis

The geometry of the MEG source space was modelled with a regular volumetric grid with 10 mm spacing created in the template MNI brain. This set of nodes was transformed to each participant’s space using a non-linear normalization between the native T_1_ image (whose coordinate system was previously converted to match the MEG coordinate system) and a standard T_1_ image in MNI space. The forward model solution used a single-shell method^34^ with a unique boundary defined by the inner skull (the combination of white matter, grey matter and CSF) extracted from each individual T_1_ image. Source reconstruction was carried out independently for each subject and frequency band with a linearly constrained minimum variance (LCMV) beamformer,^35^ using the epochs-average covariance matrix and a regularization factor of 5% of the average sensor power. This method has yielded reliable results for the estimation of resting-state functional connectivity.^36^ Each source position was labelled using the automated anatomical labelling (AAL) atlas.^37^ Only those sources labelled as part of one of the 78 cortical areas of the atlas were included in subsequent analyses (1202 nodes in total). Functional connectivity (FC) between these 1202 nodes was assessed with phase locking value (PLV), a phase synchronization measure that evaluates the distribution of phase differences extracted from two ROIs time series^38^ and has high reliability across sessions.^39^ Symmetrical, whole-brain matrices of 1202 × 1202 nodes were thus obtained by averaging PLV values across epochs for each participant and frequency band. Lastly, we computed the strength of each node (also known as weighted global connectivity), which is defined as the sum of its FC with the rest of the nodes. To account for the number of links, the strength of each node was then normalized by dividing the number of links connected to it. This procedure resulted in one brain map of normalized node strengths per each participant and frequency band.

### Statistical analyses

The assessment of significant group FC differences was based on a cluster-based permutation test (CBPT) described previously^40,41^ where the units of study were clusters of spatially adjacent nodes whose strength (weighted global connectivity) differed significantly between groups with the same sign. This procedure was applied independently for each frequency band, as implemented in Fieldtrip.^28^ The methodology started by testing each of the 1202 nodes separately for strength differences between the two groups using an ANCOVA test whilst adjusting for the effects of age. This procedure yielded one *F*-statistic value per node and resulted in a volumetric map of 1202 *F*-statistic values. This *F*-statistic map was thresholded using a critical value corresponding to the 0.005 significance level for the *F*-statistic (cluster-defining threshold). Subsequently, the thresholded map was split into two maps corresponding to the voxels with connectivity MCI EA+ >MCI EA− or MCI EA+ <MCI EA−. For each map, a clustering procedure identified groups of adjacent nodes in the volume space, and the mass of each cluster was defined as the sum of the *F*-values of the nodes comprising each cluster (cluster mass statistic). We employed this measure because it reflects a combination of the topological extent (number of nodes) and the effect size (*F*-values). As an inclusion criterion to suppress spurious findings, we required the minimum size for each candidate cluster to be equal to 1% of the total nodes in the volume, and all cluster smaller than this size were automatically deemed non-significant.

**Figure 3.**
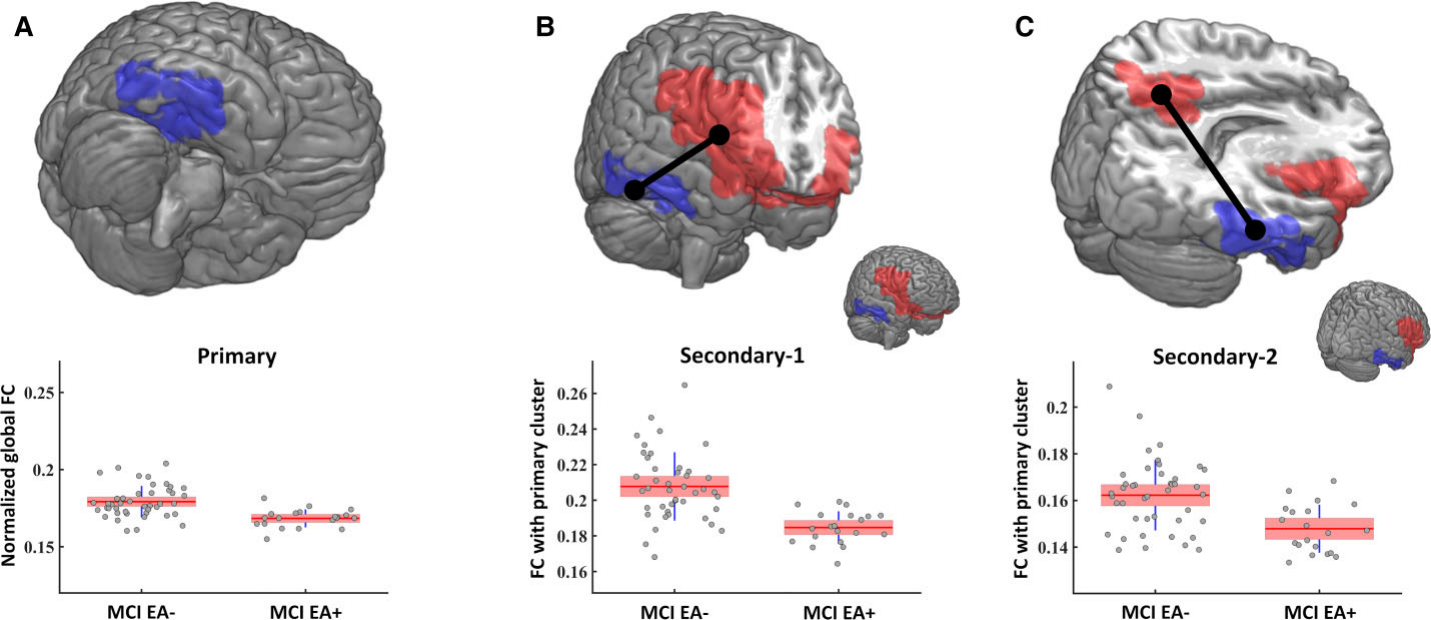
**FC results**. Significant MCI EA+ and MCI EA− network differences in the gamma band. (**A**) Dark blue region in the right temporal lobe had significantly decreased gamma band global connectivity in the MCI EA+ group (cluster named primary). (**B**, **C**) Red regions, marked as secondary-1 and secondary-2, have FC with the primary cluster significantly decreased in the MCI EA+ compared with the MCI EA− group. Black lines in **B** and **C** represent the significant FC link between the primary and the secondary clusters. Boxplots describe the FC of the corresponding cluster for each group, with dots representing individual patients.

Then, to control for multiple comparisons, this entire procedure was repeated 5000 times after randomly shuffling the original group’s labels and creating permutation samples of the original *F*-statistic maps. At each repetition, the maximum cluster mass statistic of the surrogate clusters was stored, thus constructing an empirical distribution of the maximum cluster mass statistic. This maximal null distribution enables us to compute the *P*-value for each candidate cluster of the original data and thus the control of the family-wise error rate (FWER) at the cluster level. Only those clusters that survived the CBPT at *P* < 0.05 were considered for the subsequent analyses as potential MEG markers. As a representative value of these MEG markers, we computed the average strength of the nodes contained in the cluster. These values are shown in the boxplots of Fig. 3 and were tested for differences between both groups with further ANCOVA test with age as covariate. These values were also used in a subsequent Spearman correlation analysis with cognitive and structural scores.

The significant clusters detected with the above procedure differed in global connectivity between the two groups. As a *post hoc* analysis, we applied a seed-based procedure to determine whether the strength differences (i.e. the global FC of the cluster) were primarily driven by the existence of a few isolated connections rather than caused by a ‘global/widespread’ effect (widespread connections of each original cluster to the rest of the brain). The seed region was calculated by taking all nodes that were in a radius of 20 mm from the physical mass centre of the cluster. Then, we computed all 1202 FC values between each node and the cluster’s mass centre (seed), yielding a seed connectivity map. This map was subjected to the same clustering procedure described above. Only clusters that did not overlap with the original cluster were reported in this study to ensure reliable results that did not depend on the precise extent of the original cluster. Statistical analyses were carried out using Matlab R2020b (Mathworks Inc) and all tests were two-tailed.

### Data availability

The data that support the findings of this study are available from the corresponding author, upon request. All the algorithms used in the present paper are reported in the ‘Materials and methods’ section.

## Results

Of the 63 patients in the cohort, MEG was positive for EA in 20 patients (31.75%). The average number of spikes in the patients that showed EA was 2.04 ± 1.79. Most of the spikes detected in MCI patients, were in broad regions encompassing temporal (58%) and frontal (24%) areas (see Supplementary Table 1 for specific information). None of the subjects showed a clear cluster of EA, nor had seizures. Examples of EA discharges for two patients are shown in Fig. 2.

The demographics, genetics, clinical scores and brain volumetric data information at baseline evaluation for the Madrid Cohort are shown in Table 1. There were no statistically significant differences between the patients with (MCI EA+) and without (MCI EA−) EA activity in any of the comparisons.

**Table 1:** Demographics, genetics, clinical scores and brain volumetric information of the 63 MCI patients indicating that the two groups are similar with regard to age, ApoE 4 genotyping, cognitive status and selected grey matter volumes

	MCI EA+ (*n* = 20)		MCI EA− (*n* = 43)		*P*-value
	Mean	SD	Mean	SD	
Age (years)	74.35	5.33	74.23	5.36	0.9356^a^
Gender (females)	12		24		0.7912^b^
APOE 4 genotype (%)	6		22		0.1581^b^
Education (years)	8.24	3.87	8.62	4.83	0.7719^a^
Geriatric depression scale	3.79	2.94	3.69	2.98	0.9227^a^
MMSE score	25.44	2.57	25.63	2.74	0.8141^a^
Immediate recall	15.22	7.46	13.86	10.41	0.6173^a^
Delayed recall	4.50	5.87	4.75	7.99	0.9058^a^
Forward digits	7.33	1.68	6.57	2.11	0.1800^a^
Backward digits	4.28	1.13	4.19	1.61	0.8355^a^
Left hippocampal volume	0.0022	0.0005	0.0022	0.0004	0.9289^a^
Right hippocampal volume	0.0022	0.0004	0.0022	0.0003	0.7858^a^
Total grey matter volume	512 690	61 353	527 175	51 912	0.3444^a^
Total cerebral white matter	400 070	80 787	392 009	58 289	0.6617^a^

Total grey matter and whiter matter volumes are in mm^3^. Volumes of anatomical structures are normalized by intracranial volume.

MCI, mild cognitive impairment patients; EA, +−, existence or not of epileptiform activity; MMSE, Mini Mental State Examination.

^a^ *t*-test.

^b^ Fisher’s exact test.

Next, we analysed the MEG functional networks to identify brain regions with global connectivity differences between the MCI EA+ and MCI EA− groups. We found one significant cluster (CBPT; cluster mass statistic = 268.14, *P*-value = 0.0160) in the gamma band (henceforth referred to as ‘primary’), largely focused on the right temporal region [mass centre (49 −32 −22) mm, MNI coordinates] of the brain (see Fig. 3A and Table 2, column 1). Comparing groups, the MCI EA+ cluster had significantly reduced normalized global connectivity relative to the MCI EA− group. We computed the average strength of the nodes contained in the cluster as a surrogate effect size and carried out a new ANCOVA test with age to quantify it. The values obtained of the differences at the cluster level were *P-*value < 0.001 and *F*-statistic 19.4. This result indicates that the oscillatory activity (within the gamma frequency band) of that cluster was less synchronously paired with activity from across the brain.

**Table 2:** Regions of interest (ROIs) from the AAL atlas that comprise each significant cluster

Primary cluster			Secondary-1 cluster			Secondary-2 cluster		
ROI name	%	*F* ^a^	ROI name	%	*F* ^a^	ROI name	%	*F* ^a^
rITG	54	11.9	rIFGor	77	15.4	lMCC	19	11.8
rFusiG	42	11.6	rPosG	18	15.0	lPCC	80	11.2
rMTG	3	9.3	rRO	64	14.8	lPrecu	14	10.9
			rSTG	4	14.5	rPrecu	5	9.7
			rRectus	25	14.1	lMOccL	3	9.7
			rPreCG	11	13.1	lSPG	6	9.3
			rIFGt	63	12.6			
			rSFo	33	12.3			
			rInsula	57	11.3			
			rTPmid	10	11.2			
			lInsula	36	11.1			
			lAmyg	50	10.9			
			rIFGo	58	10.8			
			lMFGo	14	10.0			
			rMFG	3	10.0			
			rTPsup	50	9.9			
			lIFGt	33	9.9			
			lIFGo	14	9.7			
			lParahip	13	9.7			
			lMFG	3	9.6			
			lIFGo	58	9.5			
			lRectus	25	9.2			

%: percentage of the ROI within the cluster.

ITG, inferior temporal gyrus; FusiG, fusiform gyrus; MTG, middle temporal gyrus; PCC, posterior cingulate gyrus; Precu, precuneus; MCC, middle cingulate gyrus; MOccL, middle occipital lobe; SPG, superior parietal gyrus; IFGt, inferior frontal gyrus triangular; IFGor, inferior frontal gyrus opercular; IFGo, inferior frontal gyrus orbital; SFGo, superior frontal gyrus orbital; RO, rolandic operculum; PosCG, postcentral gyrus; TPsup, temporal pole, superior temporal gyrus; PreCG, precentral gyrus; SFo, superior frontal gyrus orbital; MFG, middle frontal gyrus; MFGo, middle frontal gyrus orbital; Amyg, amygdala; Parahip, parahippocampus; STG, superior temporal gyrus; TPmid, temporal pole, middle temporal gyrus.

^a^ Sum of all *F*-values obtained at the node level. ROIs were ordered based on their significance (*F* column). r/l = right/left.

In order to identify specific connections that drove the global connectivity change of the primary cluster, we performed a subsequent seed-based analysis. This analysis identified the specific regions across the rest of the brain (secondary clusters) that showed significant between-group FC differences with the primary cluster. We found two secondary clusters where the FC with the original cluster was significant decreased in the MCI EA+ group as compared with the MCI EA− group. The first (referred to as ‘secondary-1’) involved mainly ipsilateral frontal and medial regions (Fig. 3B and Table 2, column 2). The average strength of the FC between the primary cluster and the secondary-1 differed between groups with the following scores *P-*value < 0.001 and *F*-statistic 25.6. The second (referred to as ‘secondary-2’) was found in the upper precuneus area (bilateral) (Fig. 3C and Table 2, column 3). In this case, the differences between groups for the average strength of the FC between the primary cluster and the secondary-2 showed a *P-*value < 0.001 and *F*-statistic 15.5.

To establish a critical link between the aberrant FC of the above clusters and scores of brain health (neurophysiological assessment and structural quantitative scores associated with grey matter atrophy), we conducted Spearman correlation analyses between these measures. For functional values, this analysis used the normalized global connectivity of the primary cluster or the FC between <primary, secondary-1> and <primary, secondary-2> (see Supplementary Table 2 for individual normalized global connectivity scores). For neurophysiological scores, we used those described in Table 1. For grey matter scores, we used those of regions contained within the significant clusters. This analysis did not yield any significant between-group differences in any correlation between the FC and the brain health scores.

Next, we conducted a similar correlation analysis, but within each group separately. This analysis yielded significant effects in the comparisons involving the FC of the primary cluster with the secondary-2 cluster in the MCI EA+ group. Specifically, the <primary, secondary-2> FC values were positively correlated with several markers of grey matter volume in the MCI EA+ group (Table 3). This result suggests that reduced FC between these two clusters is associated with higher grey matter atrophy across several brain regions in the MCI EA+ patients. We did not find significant effects for any group in all other comparisons involving the global connectivity of the primary cluster, or the FC of the primary with the secondary-1 cluster.

**Table 3.** Spearman correlation analyses between the FC of the <primary, secondary-2> clusters and brain structural integrity scores for the regions contained within the significant clusters

Structure	*r*	*P-*value*
MCI EA+		
l GM lateral orbitofrontal	0.618	0.004
l GM medial orbitofrontal	0.639	0.003
l GM pars opercularis	0.660	0.002
l GM pars orbitalis	0.553	0.013
l GM posterior cingulate	0.594	0.007
r GM lateral orbitofrontal	0.580	0.008
r GM medial orbitofrontal	0.626	0.004
r GM precuneus	0.644	0.003

l/r, left/right; GM, grey matter.

* *P-*values remained significant after FDR (*q* = 0.05) correction.

## Discussion

We evaluated MEG functional networks in MCI patients with and without EA to test whether crucial frequency bands, previously associated with memory functioning, were disrupted. If so, this would contribute to a better understanding of cognitive decline in this stage of the Alzheimer’s disease continuum. MCI patients with EA showed decreased gamma band connectivity in comparison with MCI patients without EA. The brain regions identified in this reduced gamma network involved right temporal regions, ipsilateral dorsolateral and medial frontal regions, and the upper precuneus area. Previous studies, involving non-elder patients with epilepsy, have found decreased functional connectivity in similar areas,^42^ and the diminished FC has been associated with the appearance of neurocognitive problems, including memory and language impairment.^43^ In fact, these regions are typically associated with executive functions and episodic memory in healthy subjects, and with cognitive impairment in patients with brain lesions and different types of neurological disorders.^44^

EA in patients with Alzheimer’s disease (including patients with MCI and epilepsy) has been associated with a faster decline in global cognition and executive functions^9,45,46^ as well as with a higher percentage of conversion from MCI to dementia.^47^ The existence of FC disruptions in the locations involved in the episodic memory network is consistent with our findings. Aberrant functional connectivity of this network could indicate a higher risk of compromised neurophysiological mechanisms that support memory function. Past work has shown that EA may induce transient cognitive impairment, indicating how this phenomenon interferes with cognitive processing.^48^ Since episodic memory decline is one of the initial symptoms of Alzheimer’s disease, the localization of EA at the medial temporal lobe regions could be associated with cognitive impairment. However, the scarce incidence, likely influenced for having only 20 min long recordings, and heterogeneity of localization of EA in our cohort of MCI patients, make it difficult to statistically assess this hypothesis.

It is possible that the reduction of functional connectivity in the group of MCI EA+ patients was caused by the EA itself or by the malfunction of the memory networks. In epilepsy, electrophysiological data tend to show an increased high gamma frequency (30–100 Hz) associated with EA.^49–51^ This seems to be counterintuitive with our findings assessing MCI patients in the current study. However, there are some critical differences between those studies and ours. Whilst the cited papers assessed patients with epilepsy, the presence of EA in our MCI EA+ patients were scarce (patients did not have seizures), indicating that EA itself does not sustain the network malfunction. This fact is important because the neuropathological findings in epilepsy may differ from those typically found along the Alzheimer’s disease continuum. In addition, our data consisted of non-invasive MEG recordings, whilst other studies^49,50^ were carried out using intracranial EEG. The results from these two methodologies are difficult to interpret together since the local effects found with intracranial EEG are quite different to the macroscopic signals assessed with non-invasive EEG/MEG. Besides the differences in the data and technique, there are some methodological differences such as the different gamma band definitions. We focused on the classical gamma band (30–45 Hz) rather than the broader definitions used in the referenced studies. The gamma band has been associated with episodic memory function^52^ and has been found to predict successful or unsuccessful recovery.^53^ Furthermore, patients with epilepsy show a reduction of gamma band power associated with EA in the hippocampal area during an episodic memory task.^54^ Given the differences in technique and methodology, our study offers new and different information into the underlying processes that occur in tandem with the development of Alzheimer’s disease neuropathology. Therefore, our findings of aberrant functional connectivity in MCI patients are more consistent with a disruption of the episodic memory networks. This disruption cannot be explained by the epileptogenic activity alone. In fact, we hypothesize that abnormal network functioning is a risk factor for EA, and hence that MCI EA+ patients might be at higher risk of conversion to dementia. This assumption is in line with a study by Baker *et al.*^46^ where the authors performed a longitudinal cognitive assessment of Alzheimer’s disease patients with and without a seizure history. At baseline, patients did not differ; but, after 1 year, the Alzheimer’s disease patients with a seizure history had faster cognitive decline. This suggests that EA potentially contributes to network decline.

The correlational analyses with grey matter volumes strengthen the hypothesis that our results are consistent with a disruption of memory networks. The MCI EA+ patients showed a direct relationship between brain atrophy and the reduction of the gamma band connectivity between the middle temporal gyrus and the prefrontal/parietal regions. This indicates that the functional reduction of the gamma band connectivity is accompanied by orbitofrontal, precuneus and posterior cingulate cortex volume reduction. These brain regions have been typically associated with the episodic memory network.^44^ Neurodegeneration is one of the key features of the course of the disease, forming one of the core elements of the amyloidosis- pathologic tau- neurodegeneration (ANT axis).^55^ Therefore, it seems logical that the loss of grey matter volume may affect the functional connections within the episodic memory network. This network (orbitofrontal, cingulate cortex, precuneus and the hippocampus) is densely interconnected by the cingulum bundle, which connects with the callosal splenium and through there with precuneus and prefrontal regions.^56^ Therefore, the morphological alterations of these regions could cause a depletion of the functional connectivity in the temporal lobe at a specific frequency associated with memory formation. Although we did not find differences in brain atrophy between groups, the correlation analysis revealed important associations with the functional connections pointing to an anatomo-functional dysfunction in the MCI EA+ group, suggesting that the higher the FC (i.e. the more similar it is to the MCI EA− group), the better the grey matter integrity in these patients.

There were no differences between groups in cognitive performance, nor in brain structural integrity. The only difference between groups was found for the FC assessment. However, the fact that only the EA+ group showed significant correlations with brain structural integrity might indicate that the appearance of EA could reflect alterations at the structural level. This relationship between function and structure was not found in the EA− group. The precedence in the brain resting state of functional abnormalities to any structural or cognitive damage has been stated in previous studies.^11,57^ In this study, the reported gamma FC pattern is different from the typical electrophysiological ‘slowing’ effect usually found in Alzheimer’s disease. This fact suggests that the gamma depletion could be an epiphenomenon of network disruption associated with new neuropathological pathways that could accelerate the neuronal damage induced by the dementia progression.

This study has some limitations. The MEG recordings were acquired during a resting state condition, but most of the studies referenced in our discussion found gamma band episodic memory effects during memory tasks and not at rest. Whilst this prevents a direct comparison of our findings with previous literature, it is important to highlight that many task-related networks have also been found at rest, such as the sensorimotor and social networks.^58,59^ Furthermore, there is strong evidence that associates the default mode network with episodic memory functions^60^ indicating that brain regions associated with task performance are also engaged in some default mode functions crucial to memory reorganization and engram maintenance. It is important to note that our MCI sample did not have any Alzheimer’s disease neuropathological marker (tau or amyloid). Our patients met the MCI clinical criteria of the NIA-AA and had neurodegeneration biomarkers (i.e. hippocampal grey matter measures) but more studies including tau or amyloid information will be key to understand how Alzheimer’s disease-specific network disruption may be. Another important limitation is the low incidence of EA in our MCI EA+ patients and its heterogeneous localization pattern. The low incidence could be partially underestimated due to the limited amount of data. Longer and more epilepsy-specific recordings could have allowed both the detection of more MCI EA+ patients (with a consequent decrease in the number of MCI EA−), and a better characterization of the EA in the MCI EA+ patients. Finally, to identify and exclude MEG-unique normal variants, EEG is needed. Our preliminary data were recorded without simultaneous EEG. Hence there is a possibility that source heterogeneity is inflated, and that we missed EEG unique spike discharges.

Our findings link EA, gamma band functional disruptions, and alterations contained within the memory network in our MCI patients. Moreover, they demonstrate the importance of all these factors for a better understanding of memory decline in the early stages of Alzheimer’s disease. Future studies with increased sample size, more sensitive memory tasks and longer brain recordings that are optimal for the detection and characterization of EA activity, could extend our findings and assess the potential influence of other occult factors that may have an important role in memory decline and the onset of EA.

## Supplementary Material

fcac012_Supplementary_Data

## Abbreviations

AAL =: automated anatomical labelling
CBPT =: cluster-based permutation test
EA =: epileptiform activity
EOG =: electrooculogram
FC =: functional connectivity
FWER =: family-wise error rate
HPI =: head-position indicator
LCMV =: linearly constrained minimum variance
MCI =: mild cognitive impairment
MEG =: magnetoencephalography
MNI =: Montreal Neurological Institute
NIA-AA =: National Institute on Aging and Alzheimer’s Association
PLV =: phase locking value
ROI =: region of interest
SOBI =: second-order, blind identification
tSSS =: temporal signal-space separation.
