# Supplementary material for: Gamma band functional connectivity reduction in patients with amnestic mild cognitive impairment and epileptiform activity
Source: Brain Commun. 2022 Feb 3;4(2):fcac012. doi: 10.1093/braincomms/fcac012 (PMC8914494; doi:10.1093/braincomms/fcac012)
Supplement: fcac012_Supplementary_Data [file fcac012_supplementary_data.zip › Original Submission.pdf]

OXFORD UNIVERSITY PRESS | Brain Communications

Gamma band functional connectivity reduction in patients with amnestic mild cognitive impairment and epileptogenic activity

Journal:	*Brain Communications*
Manuscript ID	BRAINCOM-2021-090
Manuscript Type:	Original Article
Date Submitted by the Author:	14-Mar-2021
Complete List of Authors:	Cuesta, Pablo; Universidad Politecnica de Madrid, Center for Biomedical Technology; Universidad Complutense de Madrid, Basic Psychology II<br>Ochoa-Urrea, M; University of Texas McGovern Medical School, Department of Neurology<br>Funke, ME; University of Texas McGovern Medical School, Department of Pediatrics<br>Hasan, O; University of Texas McGovern Medical School, Department of Neurology<br>Zhu, P; University of Texas McGovern Medical School, Vivian L. Smith Department of Neurosurgery; The University of Texas Health Science Center at Houston, Texas Institute for Restorative Neurotechnologies<br>Marcos Dolado, Alberto; Hospital Clínico Universitario San Carlos, Neurology<br>López, Maria; Universidad Politecnica de Madrid, Center for Biomedical Technology; Universidad Complutense de Madrid, Basic Psychology II<br>Schulz, Paul; University of Texas McGovern Medical School, Department of Neurology<br>Lhatoo, S; University of Texas McGovern Medical School, Department of Neurology<br>Pantazis, D; Massachusetts Institute of Technology McGovern Institute for Brain Research<br>Mosher, JC; University of Texas McGovern Medical School, Department of Pediatrics<br>Maestú, Fernando; Universidad Politecnica de Madrid, Centre for Biomedical Technology; Universidad Complutense de Madrid, Basic Psychology II
Keywords:	MCI, Epilepsy, Functional Connectivity, MEG, Gamma

SCHOLARONE™
Manuscripts

Gamma band functional connectivity reduction in patients with amnestic mild cognitive impairment and epileptogenic activity

Cuesta P[1], Ochoa-Urrea M[2], Funke ME[3], Hasan O[2], Zhu P[4,5], Marcos A[1], López ME[1], Schulz PE[2], Lhatoo S[2], Pantazis D[6], Mosher JC[3], Maestu F[1,3]

[1] Department of Experimental Psychology, Complutense University of Madrid, Madrid, Spain

[2] Department of Neurology, McGovern Medical School, The University of Texas Health Science Center at Houston, Houston, Texas, USA

[3] Department of Pediatrics, McGovern Medical School, The University of Texas Health Science Center at Houston, Houston, Texas, USA

[4] Vivian L. Smith Department of Neurosurgery, McGovern Medical School, The University of Texas Health Science Center at Houston, Houston, Texas, USA

[5] Texas Institute for Restorative Neurotechnologies, University of Texas Health Science Center at Houston

[6] McGovern Institute for Brain Research, Massachusetts Institute of Technology, Cambridge, USA

Correspondence:

For Review Only

Abstract

There is growing evidence in recent years of brain hyperexcitability in Alzheimer's disease (AD). It is associated with an increased prevalence of epileptogenic activity (EA) and disruption of inhibitory activity of interneurons, whose firing couples with high-frequency oscillations such as the gamma band (30–150Hz). It is unclear, however, how this hyperexcitability affects the organization of functional brain networks. Here we studied patients with amnestic Mild Cognitive Impairment (MCI), who underwent a Magnetoencephalography (MEG) resting-state recording with eyes closed. Of 63 patients in our Madrid cohort, we found EA in 20 patients (31.75%). A cluster-based analysis of the MEG functional connectivity revealed a region within the right temporal cortex whose global connectivity in the gamma band was significantly reduced in patients with EA relative to those without EA. A subsequent seed-based analysis showed this was largely due to weaker gamma-band connectivity of this region with ipsilateral frontal and medial regions, and the upper precuneus area. In addition, this reduced functional connectivity was associated with higher gray matter atrophy across several cortical regions in the patients with EA. These functional network disruptions and changes in brain physiology and morphology in AD have important clinical implications as they may contribute to cognitive decline in MCI and AD.

For Review Only

Introduction

Cognitive decline in the development of Alzheimer's Disease (AD) has been associated with progressive brain atrophy and the accumulation of hyperphosphorylated tau and amyloid proteins (Cummings *et al.*, 1998). A complementary factor, however, may be alterations in brain functional network organization, which is supported by a fine balance between neuronal excitation and inhibition (E/I). A disruption of this basic mechanism could cause a malfunctioning of information transmission and a breakdown of interareal communication.

In the last decade, there has been growing evidence for increased brain excitability in patients with AD. Compared to the general population, patients with AD are 8 to 10 times more likely to develop spontaneous seizures (Hauser *et al.*, 1986; Scarmeas *et al.*, 2009). Cortical hyperexcitability in AD patients could reflect a disruption of the E/I balance. This hyperexcitability has been seen in animal models, where increased neuronal firing has been found in the vicinity of amyloid plaques (Busche and Konnerth, 2016). It has also been found in humans where amyloid toxicity has caused a loss of inhibitory terminals (Garcia-Marin *et al.*, 2009). Finally, increased epileptogenic activity (EA) has been found in brain regions typically affected by the neuropathology of AD (Vossel *et al.*, 2013; Vossel KA *et al.*, 2016; Brunetti *et al.*, 2020; Lam *et al.*, 2020).

This hyperexcitability could lead to increased neuronal synchronization and a dysfunctional organization of the profiles of brain activity shown at multiple frequency bands with electroencephalography (EEG) and magnetoencephalography (MEG). Increased phase synchrony between the anterior and posterior regions has been found in mid-adult humans with amyloid deposition (Nakamura *et al.*, 2017) and in relatives of AD patients (Ramírez-Toraño *et al.*, 2020), all at preclinical stages. Furthermore, this neurophysiological signature was also found in elders with subjective cognitive decline (López-Sanz *et al.*, 2017) and in Mild Cognitive Impairment (MCI) patients who later progressed to dementia (López *et al.*, 2016; Pusil *et al.*, 2019).

Palop and Mucke noted a link between cognitive impairment and disruption of interneuron inhibitory activity in a comprehensive review in 2016 (Palop and Mucke, 2016). In addition, the firing of interneurons is more prominent and synchronized with high-frequency oscillations, such as those in the gamma band (30–150 Hz). The gamma band has been frequently associated with local and long-distance communication (Rouhinen *et al.*, 2020) and with memory function by predicting those items that will successfully be recalled later (Sederberg *et al.*, 2007). A reduction of the gamma band power has been associated with the appearance of epileptic discharges in an animal model of AD (Maheshwari *et al.*, 2016). Furthermore, patients with epilepsy tend to show higher gamma band activity during successful encoding of words (Matsumoto *et al.*, 2013). Thus, extensive evidence now directly links the gamma band, memory formation, and interneuron modulatory activity.

The *goal* of this study was to test whether hyperexcitability in the form of EA found in MCI patients induces alterations of crucial oscillatory activity associated with memory formation. We *hypothesized* that there would be greater network dysfunction in MCI with EA than without EA in correlation with grey matter volume alterations due to neurodegeneration.

For Review Only

Materials and Methods

Subjects

Sixty-three amnestic MCI subjects were recruited from the Hospital Universitario San Carlos (Madrid, Spain). All were native Spanish speakers and right-handed. The MCI diagnosis was established according to the NIA-AA criteria (Albert *et al.*, 2011), which includes (i) self- or informant- reported cognitive complaints, (ii) objective evidence of impairment in one or more cognitive domains, (iii) preserved independence in functional abilities, and (iv) not demented (McKhann *et al.*, 2011). For more information about the diagnostic criteria for MCI see (López *et al.*, 2020). None of the participants exhibited a history of psychiatric or neurological disorders other than MCI or AD. General inclusion criteria were as follows: age between 60 and 90 years; a modified Hachinski score ≤ 4; a short form Geriatric Depression Scale score ≤ 5; and, a T1/T2-weighted MRI within 54 weeks before the MEG recordings without indication of infection, infarction, or focal lesions (rated by two independent experienced radiologists (Bai *et al.*, 2012)). In addition, we advised subjects to avoid medications that could affect MEG activity, such as benzodiazepines, for 48 hours before recordings. All participants provided written, informed consent. The Institutional Review Board Ethics Committee at Hospital Universitario San Carlos approved the study protocol and the procedure was performed following the Helsinki Declaration and National and European Union regulations.

MRI acquisition and volumetric analyses

T1-weighted MRI images from each participant were acquired with a General Electric 1.5 T MRI scanner using a high-resolution antenna and a homogenization PURE filter (Fast Spoiled Gradient Echo sequence, TR/TE/TI = 11.2/4.2/450 ms; flip angle 12°; 1 mm slice thickness, 256x256 matrix and FOV 25 cm). The resulting images were processed using Freesurfer software (version 5.1.0) and its specialized tool for automated cortical parcellation and subcortical segmentation (Fischl, 2012).

MEG recordings and interpretation

MEG signals were acquired using a whole-head Elekta-Neuromag MEG system with 306 channels (Elekta AB, Stockholm, Sweden) at the Center for Biomedical Technology (Madrid, Spain). Data was collected at a sampling frequency of 1000 Hz and online band-pass filtered between 0.1 and 330 Hz.

The MEG protocol consisted of a 5 minute, eyes-closed, resting-state MEG recording while sitting comfortably inside a magnetically shielded room. Participants were asked to stay awake and to minimize their body movements. Each participant's head shape was defined relative to three anatomical locations (nasion and bilateral preauricular points) using a 3D digitizer (Fastrak, Polhemus, VT, USA) and head motion was tracked through four head-position indicator (HPI) coils attached to the scalp. These HPI coils continuously monitored the subjects' head movements, while eye movements were monitored by a vertical electro-oculogram (EOG) assembly composed of a pair of bipolar electrodes. Raw data was first processed with Maxfilter software (v 2.2, temporal signal-space separation (tSSS), correlation threshold = 0.9, time window = 10 seconds) to remove external noise using the temporal extension of the signal space separation method with movement compensation (Taulu and Simola, 2006). Data underwent automatic artifact

For Review Only

selection using FieldTrip (Oostenveld *et al.*, 2011), and a MEG expert confirmed the findings. After the artifact's removal, we applied second-order, blind identification (SOBI) (Belouchrani *et al.*, 1997) to remove cardiographic, oculographic, and noise-related components. Only the magnetometers' data were used for subsequent analysis since, after Maxfilter processing, the sensor-space data is highly redundant (Garcés *et al.*, 2017). The remaining artefact-free data were segmented into 4-s segments (epochs). Only those recordings with at least 20 clean segments (80 seconds of brain activity) were utilized in subsequent analyses. Prior to source estimation, the MEG time courses were filtered into theta (4.1-7.9 Hz), alpha (8.1-11.9 Hz), beta (12.1-29.9 Hz), and gamma (30.1-45.0 Hz) frequency bands with a 1500 order finite impulse response filter using a Hamming window and a two-pass filtering procedure.

A MEG expert (MEF) and a neurologist trained in MEG reading (MOU) screened MEG signals from the left and right fronto-temporal montages for EA. Raw data was reviewed after Maxfilter processing. We defined EA as a transient signal that was clearly distinguished from background activity, and with a pointed peak component (Fernandes *et al.*, 2005; Nowak *et al.*, 2009). In order to be valid, the transients had to have an amplitude greater than 100 nanoAmpers, a goodness of fit greater than 80%, and a volume less than 1000 mm^2. Source localization of the dipoles was performed in the DANA Elekta Neuromag Software (Elekta AB, Stockholm, Sweden) superimposing the dipoles on the patient's structural MRI. Depending on the presence or absence of EA, patients were divided into two groups: MCI EA+ and MCI EA-, comprising patients with and without EA, respectively.

Source Reconstruction and Connectivity Analysis

The geometry of the MEG source space was modeled with a regular grid with 10 mm spacing created in the template MNI brain. This set of nodes was transformed to each participant's space using a non-linear normalization between the native T1 image (whose coordinate system was previously converted to match the MEG coordinate system) and a standard T1 image in MNI space. The forward model solution used a single-shell method (Nolte, 2003) with a unique boundary defined by the inner skull (the combination of white matter, grey matter and CSF) extracted from each individual T1 image. Source reconstruction was carried out independently for each subject and frequency band with a linearly constrained minimum variance (LCMV) beamformer (Van Veen *et al.*, 1997), using the trial-average covariance matrix and a regularization factor of 5% of the average sensor power. This method has yielded reliable results for the estimation of resting-state functional connectivity (Hincapié *et al.*, 2017). Each source position was labeled using the Automated Anatomical Labeling (AAL) atlas (Tzourio-Mazoyer *et al.*, 2002). Only those sources labeled as part of one of the 78 cortical areas of the atlas were included in subsequent analyses (1202 nodes in total). Functional connectivity (FC) between these 1202 nodes was assessed with phase locking value (PLV), a phase synchronization measure that evaluates the distribution of phase differences extracted from two ROIs time series (Mormann *et al.*, 2000) and has high reliability across sessions (Garces *et al.*, 2016). Symmetrical, whole-brain matrices of 1202x1202 nodes were thus obtained by averaging PLV values across trials for each participant and frequency band. Lastly, we computed the *strength* of each node (also known as weighted global connectivity), which is defined as the sum of its FC with the rest of the nodes. To account for the number of links, the strength of each node was then normalized by dividing the number of links connected to it. This procedure resulted in one brain map of normalized node strengths per each participant and frequency band.

For Review Only

Statistical analyses

The assessment of significant group FC differences was based on a cluster-based permutation test (CBPT) described previously (Maris and Oostenveld, 2007; Zalesky *et al.*, 2010) where the units of study were clusters of spatially adjacent nodes whose strength (weighted global connectivity) differed significantly between groups with the same sign. This procedure was applied independently for each frequency band. The methodology started by testing each of the 1202 nodes separately for strength differences between the two groups using an ANCOVA test while adjusting for the effects of age. This procedure yielded one F-statistic value per node. The resulting brain map of F-statistics was thresholded using a critical value corresponding to a *p*-value of 0.005 and was then split into two brain maps corresponding to positive and negative differences (of node strengths) between the two groups. Candidate clusters were required to have at least 1% of the total nodes (i.e. 12 nodes). Cluster mass statistics were assessed through the sum of all F-values of the nodes belonging to the cluster. Then, to control for multiple comparisons, this procedure was repeated 5000 times after randomly shuffling the original group's labels. At each repetition, the maximum statistic of the surrogate clusters was stored, creating a maximal null distribution that ensured the control of the family-wise error rate (FWER) at the cluster level. The resulting network based statistic (NBS) *p*-value for a candidate cluster corresponded with the proportion of the permutation distribution with cluster-statistic values greater than or equal to the network-statistic value of the original data. Only those clusters that survived the NBS at $p < 0.05$ were considered for the subsequent analyses as potential MEG markers.

The significant clusters detected with the above procedure differed in global connectivity between the two groups. In order to determine whether these global connectivity differences were primarily attributed to a few isolated, local connections, we performed an additional analysis using the mass center of the previous clusters as seeds. The seed region was calculated by taking all nodes that were in a radius of 20 mm from the mass center of the cluster. In this post hoc analysis, we evaluated the existence of group differences in the FC between each node of the grid and those included in the seed region. Using these new node-to-seed FC, additional cluster-based analyses were performed with the same parameters as the original analysis. Only clusters that did not overlap with the original seed-cluster were reported in this study.

As descriptive values for each significant cluster, we computed its *normalized global connectivity*, defined as the normalized node strength averaged across all nodes that belong to the cluster. These values were used as FC marker values for a subsequent Spearman correlation analysis with neuropsychological and structural scores. In addition, we computed pairwise statistics, between groups, for these MEG markers using ANCOVA with age as covariate. Statistical analyses were carried out using Matlab R2020b (Mathworks Inc) and all tests were two-tailed.

For Review Only

Results

The demographics, genetics, clinical scores, and brain volumetric data information at baseline evaluation for the Madrid Cohort are shown in Table 1. There were no statistically significant differences between the patients with (MCI EA+) and without (MCI EA-) epileptogenic activity in any of the comparisons. Of the 63 patients in the cohort, MEG was positive for EA in 20 patients (31.75%).

Table 1. There were no differences between the mild cognitive impairment patients with (MCI EA+) and without (MCI EA-) epileptogenic activity in terms of demographics, ApoE-ε4, cognitive scores, and brain volumetric data. Statistical tests were computed with Fisher's exact test for Age and ApoE-ε4, and t-tests in all other cases. MMSE: Mini Mental State Examination; forward and backward digit span measures working memory; delayed recall measures episodic memory; total grey matter and whiter matter volumes are in mm^3; volumes of anatomical structures are normalized by intracranial volume.

	MCI EA+ (n=20)		MCI EA- (n=43)		
	Mean	Sd	Mean	Sd	p-value
Age (years)	74,35	5,33	74,23	5,36	0,9356
Gender (females)	12		24		0,7912
APOE 4 genotype (%)	6		22		0,1581
Education (years)	8,24	3,87	8,62	4,83	0,7719
Geriatric depression scale	3,79	2,94	3,69	2,98	0,9227
MMSE score	25,44	2,57	25,63	2,74	0,8141
Immediate recall	15,22	7,46	13,86	10,41	0,6173
Delayed recall	4,50	5,87	4,75	7,99	0,9058
Forward digits	7,33	1,68	6,57	2,11	0,1800
Backward digits	4,28	1,13	4,19	1,61	0,8355
Left hippocampal volume	0,0022	0,0005	0,0022	0,0004	0,9289
Right hippocampal volume	0,0022	0,0004	0,0022	0,0003	0,7858
Total grey matter volume	512690	61353	527175	51912	0,3444
Total cerebral white matter	400070	80787	392009	58289	0,6617

For Review Only

Next, we analyzed the MEG functional networks to identify brain regions with global connectivity differences between the MCI EA+ and MCI EA- groups. We found one significant cluster (CBPT, p-value = 0.0204) in the gamma band (henceforth referred to as "primary"), largely focused in the right temporal region (mass center [49 -32 -22] mm, MNI coordinates) of the brain (see Figure 1A and Table 2, column 1). Comparing groups, the MCI EA+ cluster had significantly reduced normalized, global connectivity versus the MCI EA- group. This indicates that the oscillatory activity (within the gamma frequency band) of that cluster is less synchronously paired with activity from all across the brain.

In order to identify specific connections that drove the global connectivity change of the primary cluster, we performed a subsequent seed-based analysis. This analysis identified the specific regions across the rest of the brain (secondary clusters) that showed significant between-group FC differences with the primary cluster. We found two secondary clusters where the FC with the original cluster was significant decreased in the MCI EA+ group as compared to the MCI EA- group. The first (referred to as "secondary-

1") involved ipsilateral frontal and medial regions (Figure 1B & Table 2, column 2), whereas the second (referred to as "secondary-2") was found in the upper precuneus area (Figure 1C & Table 2, column 3).

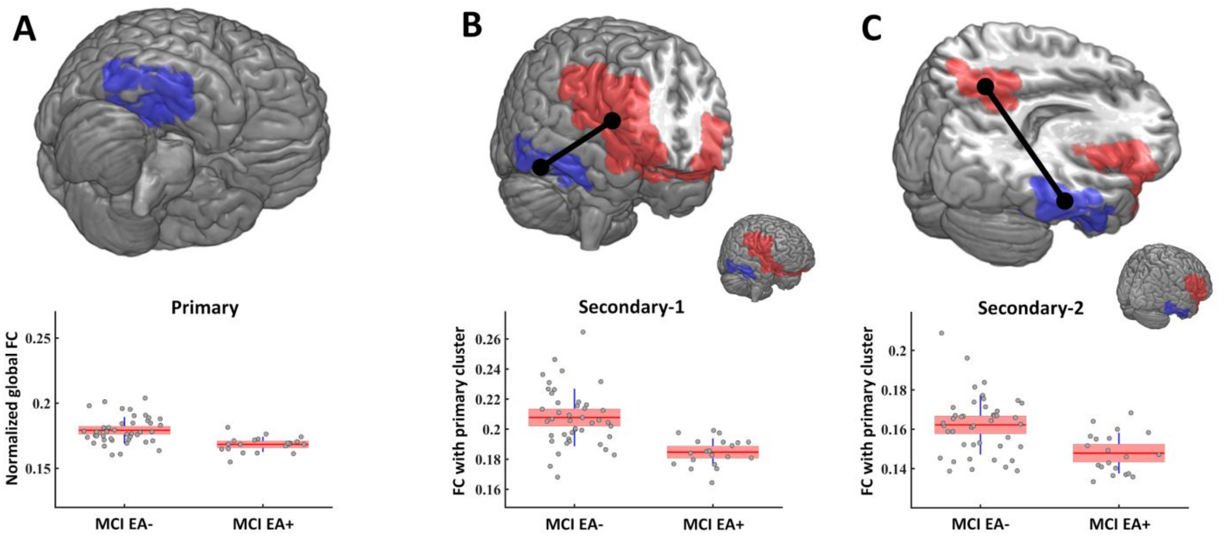

Figure 1. Significant MCI EA+ and MCI EA- network differences in the gamma band. (A) Dark blue region in the right temporal lobe had significantly decreased gamma band global connectivity in the MCI EA+ group (cluster named primary). (BC) Red regions, marked as secondary-1 and secondary-2, have FC with the primary cluster significantly decreased in the MCI EA+ compared to the MCI EA- group. Black lines in B and C represent the significant FC link between the primary and the secondary clusters. Boxplots describe the FC of the corresponding cluster for each group, with dots representing individual patients.

Review Only

Table 2. Regions of interest (ROIs) from the AAL atlas that comprise each significant cluster. Columns '%' depict the percentage of the ROI that fall within the cluster. Columns 'F' show the average F value of the cluster (resulting from summing all F values obtained in the statistics at the node level). ROIs were ordered based on their significance (F column). First letter of the ROIs acronyms denotes laterality (right, r; left, l). ITG: inferior temporal gyrus; FusiG: fusiform gyrus; MTG: middle temporal gyrus; PCC: posterior cingulate gyrus; Precu: precuneus; MCC: middle cingulate gyrus; MOccL: middle occipital lobe; SPG: superior parietal gyrus; IFGt: inferior frontal gyrus triangular; IFGor: inferior frontal gyrus opercular; IFGo: inferior frontal gyrus orbital; SFGo: superior frontal gyrus orbital; RO: rolandic operculum; PosCG: postcentral gyrus; TPsup: temporal pole, superior temporal gyrus; PreCG: precentral gyrus; SFo: superior frontal gyrus orbital; MFG: middle frontal gyrus; MFGo: middle frontal gyrus orbital; Amyg: amygdala; Parahip: parahippocampus; STG: superior temporal gyrus; TPmid: temporal pole, middle temporal gyrus.

**Primary cluster**			**Secondary-1 cluster**			**Secondary-2 cluster**		
**ROI name**	**%**	**F**	**ROI name**	**%**	**F**	**ROI name**	**%**	**F**
rITG	54	11,9	rIFGor	77	15,4	lMCC	19	11,8
rFusiG	42	11,6	rPosG	18	15,0	lPCC	80	11,2
rMTG	3	9,3	rRO	64	14,8	lPrecu	14	10,9
			rSTG	4	14,5	rPrecu	5	9,7
			rRectus	25	14,1	lMOccL	3	9,7
			rPreCG	11	13,1	lSPG	6	9,3
			rIFGt	63	12,6			
			rSFo	33	12,3			
			rInsula	57	11,3			
			rTPmid	10	11,2			
			lInsula	36	11,1			
			lAmyg	50	10,9			
			rIFGo	58	10,8			
			lMFGo	14	10,0			
			rMFG	3	10,0			
			rTPsup	50	9,9			
			lIFGt	33	9,9			
			lIFGo	14	9,7			
			lParahip	13	9,7			
			lMFG	3	9,6			
			lIFGo	58	9,5			
			lRectus	25	9,2			

For Review Only

To establish a critical link between the aberrant FC of the above clusters and scores of brain health (neurophysiological assessment and structural quantitative scores associated with grey matter atrophy), we conducted Spearman correlation analyses between these measures. For functional values, this analysis used the normalized global connectivity of the primary cluster or the FC between <primary, secondary-1> and <primary, secondary-2>. For neurophysiological scores, we used those described in Table 1. For grey matter scores, we used those of regions contained within the significant clusters. This analysis did not yield any significant between-group differences in any correlation between the FC and the brain health scores.

Next, we conducted a similar correlation analysis, but within each group separately. This analysis yielded significant effects in the comparisons involving the FC of the primary cluster with the secondary-2

cluster in the MCI EA+ group. Specifically, the <primary, secondary-2> FC values were positively correlated with several markers of gray matter volume in the MCI EA+ group (Table 3). This result suggests that reduced FC between these two clusters is associated with higher gray matter atrophy across several brain regions in the MCI EA+ patients. We did not find significant effects for any group in all other comparisons involving the global connectivity of the primary cluster, or the FC of the primary with the secondary-1 cluster.

For Review Only

Table 3. Spearman correlation analyses between the FC of the <primary, secondary-2> clusters and brain structural integrity scores for the regions contained within the significant clusters. l: left, r: right, gm: grey matter.

MCI EA+

Structure	***r***	***p value***
l gm lateral orbitofrontal	0,618	0,004
l gm medial orbitofrontal	0,639	0,003
l gm pars opercularis	0,660	0,002
l gm pars orbitalis	0,553	0,013
l gm posterior cingulate	0,594	0,007
r gm lateral orbitofrontal	0,580	0,008
r gm medial orbitofrontal	0,626	0,004
r gm precuneus	0,644	0,003

For Review Only

Discussion

We evaluated MEG functional networks in MCI patients with and without EA to test whether crucial frequency bands, previously associated with memory functioning, are disrupted. If so, it would contribute to a much better understanding of cognitive decline in this stage of the AD continuum. MCI patients with EA showed a decreased gamma band connectivity in comparison with MCI patients without EA. The brain regions identified in this reduced gamma network involved right temporal regions, ipsilateral dorsolateral and medial frontal regions, and upper precuneus area. This areas and behavior, decreased FC in the epileptic patients, has been found in previous studies involving non elders (Englot *et al.*, 2015) and they have associated with the appearance of neurocognitive problems, including memory and language disturbances (Englot *et al.*, 2016). In fact, these regions are typically associated with executive functions and episodic memory in healthy subjects, and with cognitive impairment in patients with brain lesions and different types of neurological disorders (Ferguson *et al.*, 2019).

The gamma band has been associated with episodic memory function (Lin *et al.*, 2019) and predicts successful or unsuccessful recovery (Tan *et al.*, 2020). Furthermore, patients with epilepsy showed a reduction of gamma band power associated with EA during an episodic memory task in the hippocampal area (Lega *et al.*, 2015). Interestingly, these gamma band effects were found in a very similar network as the one described here.

EA in patients with AD (including patients with MCI) has been associated with a faster decline in global cognition and executive functions (Vossel KA *et al.*, 2016) as well as with a higher percentage of conversion (Vossel *et al.*, 2017). This is consistent with our findings that there are localized, functional connectivity disruptions in the brain regions involved in the episodic memory network. Aberrant functional connectivity of this network could indicate a higher risk of compromised neurophysiological mechanisms that support memory function.

The correlational analyses with grey matter volumes reinforce our results. The MCI EA+ patients showed a direct relationship between brain atrophy and reduction of the γ-band connectivity between the middle temporal gyrus and the prefrontal/parietal regions. This indicates that the functional depletion of the γ-band connectivity is accompanied by orbitofrontal, precuneus, and posterior cingulate cortex volume reduction. These brain regions have been typically associated with the episodic memory network (Ferguson *et al.*, 2019).. Neurodegeneration is one of the key features of the course of the disease, forming one of the core elements of the ANT axis (Jack *et al.*, 2017). Therefore, it seems logical that the loss of grey matter volume is affecting the functional connections within the episodic memory network. This network (orbitofrontal, cingulate cortex, precuneus and the hippocampus) is densely interconnected by the cingulum bundle, which connects with the callosal splenium and trough there with precuneus and prefrontal regions (Bubb *et al.*, 2018). Therefore, the morphological alterations of these regions could cause a depletion of the functional connectivity in the temporal lobe at a specific frequency associated with memory formation. Although we did not find differences in brain atrophy between groups, the correlation analysis revealed important associations with the functional connections pointing to an anatomo-functional dysfunction in the MCI EA+ group.

For Review Only

On the other hand, we found that the FC of the edge [main-γ, seed2-γ] was associated with better indicators of healthy brain structural integrity. In particular, we found a direct relationship in the MCI EA+ group, suggesting that the higher the FC, the better the grey matter integrity in these patients.

This study has some limitations. The MEG recordings were acquired during a resting state condition, but most of the studies referenced in our discussion found gamma-band episodic memory effects during memory tasks and not at rest. While this prevents a direct comparison of our findings with previous literature, it is important to highlight that many task-related networks have also been found at rest, such as the sensorimotor and social networks (Biswal *et al.*, 1995; Yeshurun *et al.*, 2021). Furthermore, there is strong evidence that associates the default mode network with episodic memory functions (Sestieri *et al.*, 2011) indicating that brain regions associated with task performance are also engaged in some default mode functions crucial to memory reorganization and engram maintenance.

In MCI patients, our findings link EA, gamma-band functional disruptions, and alterations contained within the memory network, and demonstrate the importance of all these factors for better understanding memory decline in the early stages of AD. Future studies with increased sample size, more sensitive memory tasks, and longer brain recordings that are optimal for the detection and characterization of EA activity, could codify our findings and assess the potential influence of other occult factors that may have an important role in memory decline and the onset of epileptogenic activity.

For Review Only

References

Albert MS, DeKosky ST, Dickson D, Dubois B, Feldman HH, Fox NC, et al. The diagnosis of mild cognitive impairment due to Alzheimer's disease: recommendations from the National Institute on Aging-Alzheimer's Association workgroups on diagnostic guidelines for Alzheimer's disease. Alzheimers Dement 2011; 7: 270–9.

Bai Y, Hu Y, Wu Y, Zhu Y, He Q, Jiang C, et al. A prospective, randomized, single-blinded trial on the effect of early rehabilitation on daily activities and motor function of patients with hemorrhagic stroke. J Clin Neurosci 2012; 19: 1376–9.

Belouchrani A, Abed-Meraim K, Cardoso JF, Moulines E, -. Cardoso J, Moulines E. A blind source separation technique using second-order statistics. IEEE Trans Signal Process 1997; 45: 434–44.

Biswal B, Yetkin FZ, Haughton VM, Hyde JS. Functional connectivity in the motor cortex of resting human brain using echo-planar MRI. Magn Reson Med 1995; 34: 537–41.

Brunetti V, D'Atri A, Della Marca G, Vollono C, Marra C, Vita MG, et al. Subclinical epileptiform activity during sleep in Alzheimer's disease and mild cognitive impairment. Clin Neurophysiol 2020; 131: 1011–8.

Bubb EJ, Metzler-Baddeley C, Aggleton JP. The cingulum bundle: Anatomy, function, and dysfunction. Neurosci Biobehav Rev 2018; 92: 104–27.

Busche MA, Konnerth A. Impairments of neural circuit function in Alzheimer's disease. Philos Trans R Soc Lond B Biol Sci 2016; 371

Cummings JL, Vinters H V, Cole GM, Khachaturian ZS. Alzheimer's disease Etiologies, pathophysiology, cognitive reserve, and treatment opportunities. Neurology 1998; 51: S2–17.

Englot DJ, Hinkley LB, Kort NS, Imber BS, Mizuiri D, Honma SM, et al. Global and regional functional connectivity maps of neural oscillations in focal epilepsy. Brain 2015; 138: 2249–62.

Englot DJ, Konrad PE, Morgan VL. Regional and global connectivity disturbances in focal epilepsy, related neurocognitive sequelae, and potential mechanistic underpinnings. Epilepsia 2016; 57: 1546–57.

Ferguson MA, Lim C, Cooke D, Darby RR, Wu O, Rost NS, et al. A human memory circuit derived from brain lesions causing amnesia. Nat Commun 2019; 10: 3497.

Fernandes JM, da Silva AM, Huiskamp G, Velis DN, Manshanden I, de Munck JC, et al. What does an epileptiform spike look like in MEG? Comparison between coincident EEG and MEG spikes. J Clin Neurophysiol 2005; 22: 68–73.

Fischl B. FreeSurfer. Neuroimage 2012; 62: 774–81.

Garcés P, López-Sanz D, Maestú F, Pereda E. Choice of magnetometers and gradiometers after signal space separation. Sensors (Switzerland) 2017; 17: 2926.

Garces P, Martin-Buro MC, Maestu F. Quantifying the test-retest reliability of MEG resting state functional connectivity. Brain Connect 2016; 6: brain.2015.0416.

Garcia-Marin V, Blazquez-Llorca L, Rodriguez J-R, Boluda S, Muntane G, Ferrer I, et al. Diminished perisomatic {GABAergic} terminals on cortical neurons adjacent to amyloid plaques. Front Neuroanat 2009; 3: 28.

Hauser WA, Morris ML, Heston LL, Anderson VE. Seizures and Myoclonus in Patients with Alzheimer's Disease. Neurology 1986; 36: 1226–30.

Hincapié A-SS, Kujala J, Mattout J, Pascarella A, Daligault S, Delpuech C, et al. The impact of MEG source reconstruction method on source-space connectivity estimation: A comparison between minimum-norm solution and beamforming. Neuroimage 2017; 156: 29–42.

Jack CR, Wiste HJ, Weigand SD, Therneau TM, Lowe VJ, Knopman DS, et al. Defining imaging biomarker cut points for brain aging and Alzheimer's disease. Alzheimers Dement 2017; 13: 205–16.

Lam AD, Sarkis RA, Pellerin KR, Jing J, Dworetzky BA, Hoch DB, et al. Association of epileptiform abnormalities and seizures in Alzheimer disease. Neurology 2020; 95: e2259–70.

Lega B, Dionisio S, Bingaman W, Najm I, Gonzalez-Martinez J. The gamma band effect for episodic memory encoding is absent in epileptogenic hippocampi. Clin Neurophysiol 2015; 126: 866–72.

Lin J-JJ, Umbach G, Rugg MD, Lega B. Gamma oscillations during episodic memory processing provide

For Review Only

evidence for functional specialization in the longitudinal axis of the human hippocampus. Hippocampus 2019; 29: 68–72.
López-Sanz D, Bruña R, Garcés P, Martín-Buro MC, Walter S, Delgado ML, et al. Functional Connectivity Disruption in Subjective Cognitive Decline and Mild Cognitive Impairment: A Common Pattern of Alterations. Front Aging Neurosci 2017; 9: 109.
López ME, Turrero A, Cuesta P, Rodríguez-Rojo IC, Barabash A, Marcos A, et al. A multivariate model of time to conversion from mild cognitive impairment to Alzheimer's disease. Geroscience 2020; 42: 1715–32.
López MEME, Turrero A, Cuesta P, López-Sanz D, Bruña R, Marcos A, et al. Searching for Primary Predictors of Conversion from Mild Cognitive Impairment to Alzheimer's Disease: A Multivariate Follow-Up Study. J Alzheimer's Dis 2016; 52: 133–43.
Maheshwari A, Marks RL, Yu KM, Noebels JL. Shift in interictal relative gamma power as a novel biomarker for drug response in two mouse models of absence epilepsy. Epilepsia 2016; 57: 79–88.
Maris E, Oostenveld R. Nonparametric statistical testing of EEG- and MEG-data. J Neurosci Methods 2007; 164: 177–90.
Matsumoto JY, Stead M, Kucewicz MT, Matsumoto AJ, Peters PA, Brinkmann BH, et al. Network oscillations modulate interictal epileptiform spike rate during human memory. Brain 2013; 136: 2444–56.
McKhann G, Knopman DS, Chertkow H, Hymann B, Jack CR, Kawas C, et al. The diagnosis of dementia due to Alzheimer's disease: Recommendations from the National Institute on Aging- Alzheimer's Association workgroups on diagnostic guidelines for Alzheimer's disease. Alzheimers Dement 2011; 7: 263–9.
Mormann F, Lehnertz K, David P, E. Elger C. Mean phase coherence as a measure for phase synchronization and its application to the EEG of epilepsy patients. Phys D Nonlinear Phenom 2000; 144: 358–69.
Nakamura A, Cuesta P, Kato T, Arahata Y, Iwata K, Yamagishi M, et al. Early functional network alterations in asymptomatic elders at risk for Alzheimer's disease [Internet]. Sci Rep 2017; 7Available from: https://doi.org/10.1038%2Fs41598-017-06876-8
Nolte G. The magnetic lead field theorem in the quasi-static approximation and its use for magnetoencephalography forward calculation in realistic volume conductors. Phys Med Biol 2003; 48: 3637–52.
Nowak R, Santiuste M, Russi A. Toward a definition of MEG spike: parametric description of spikes recorded simultaneously by MEG and depth electrodes. Seizure 2009; 18: 652–5.
Oostenveld R, Fries P, Maris E, Schoffelen J-M. FieldTrip: Open source software for advanced analysis of MEG, EEG, and invasive electrophysiological data. Comput Intell Neurosci 2011; 2011: 156869.
Palop JJ, Mucke L. Network abnormalities and interneuron dysfunction in Alzheimer disease. Nat Rev Neurosci 2016; 17: 777–92.
Pusil S, López ME, Cuesta P, Bruña R, Pereda E, Maestú F. Hypersynchronization in mild cognitive impairment: the 'X' model. Brain 2019; 142: 3936–50.
Ramírez-Toraño F, Bruña R, de Frutos-Lucas J, Rodríguez-Rojo IC, Marcos de Pedro S, Delgado-Losada ML, et al. Functional Connectivity Hypersynchronization in Relatives of Alzheimer's Disease Patients: An Early E/I Balance Dysfunction? Cereb Cortex 2020: 1–10.
Rouhinen S, Siebenhühner F, Palva JM, Palva S, Matias Palva J, Palva S. Spectral and Anatomical Patterns of Large-Scale Synchronization Predict Human Attentional Capacity. Cereb Cortex 2020; 30: 5293–308.
Scarmeas N, Honig LS, Choi H, Cantero J, Brandt J, Blacker D, et al. Seizures in Alzheimer Disease: Who, When, and How Common? Arch Neurol 2009; 66: 992–97.
Sederberg PB, Schulze-Bonhage A, Madsen JR, Bromfield EB, McCarthy DC, Brandt A, et al. Hippocampal and neocortical gamma oscillations predict memory formation in humans. Cereb Cortex 2007; 17: 1190–6.
Sestieri C, Corbetta M, Romani GL, Shulman GL. Episodic memory retrieval, parietal cortex, and the default mode network: functional and topographic analyses. J Neurosci 2011; 31: 4407–20.
Tan RJ, Rugg MD, Lega BC. Direct brain recordings identify hippocampal and cortical networks that

For Review Only

distinguish successful versus failed episodic memory retrieval. Neuropsychologia 2020; 147: 107595.
Taulu S, Simola J. Spatiotemporal signal space separation method for rejecting nearby interference in MEG measurements. Phys Med Biol 2006; 51: 1759–68.
Tzourio-Mazoyer N, Landeau B, Papathanassiou D, Crivello F, Etard O, Delcroix N, et al. Automated anatomical labeling of activations in SPM using a macroscopic anatomical parcellation of the MNI MRI single-subject brain. Neuroimage 2002; 15: 273–89.
Van Veen BD, van Drongelen W, Yuchtman M, Suzuki A, Veen BD Van, Drongelen W Van, et al. Localization of brain electrical activity via linearly constrained minimum variance spatial filtering. IEEE Trans Biomed Eng 1997; 44: 867–80.
Vossel KA, Beagle AJ, Rabinovici GD, Shu H, Lee SE, Naasan G, et al. Seizures and epileptiform activity in the early stages of Alzheimer disease. JAMA Neurol 2013; 70: 1158–66.
Vossel KA, Ranasinghe KG, Beagle AJ, Mizuiri D, Honma SM, Dowling AF, et al. Incidence and Impact of Subclinical Epileptiform Activity in Alzheimer's Disease. Ann Neurol 2016: 1–54.
Vossel KA, Tartaglia MC, Nygaard HB, Zeman AZ, Miller BL. Epileptic activity in Alzheimer's disease: causes and clinical relevance. Lancet Neurol 2017; 16: 311–22.
Yeshurun Y, Nguyen M, Hasson U. The default mode network: where the idiosyncratic self meets the shared social world. Nat Rev Neurosci 2021; 22: 181–92.
Zalesky A, Fornito A, Bullmore ET. Network-based statistic: Identifying differences in brain networks. Neuroimage 2010; 53: 1197–207.

For Review Only
