# Supplementary material for: Gamma band functional connectivity reduction in patients with amnestic mild cognitive impairment and epileptiform activity
Source: Brain Commun. 2022 Feb 3;4(2):fcac012. doi: 10.1093/braincomms/fcac012 (PMC8914494; doi:10.1093/braincomms/fcac012)
Supplement: fcac012_Supplementary_Data [file fcac012_supplementary_data.zip › Revision 1.pdf]

OXFORD UNIVERSITY PRESS | Brain Communications

Gamma band functional connectivity reduction in patients with amnestic mild cognitive impairment and epileptiform activity

Journal:	*Brain Communications*
Manuscript ID	BRAINCOM-2021-090.R1
Manuscript Type:	Original Article
Date Submitted by the Author:	03-Aug-2021
Complete List of Authors:	Cuesta, Pablo; Universidad Politecnica de Madrid, Center for Biomedical Technology; Universidad Complutense de Madrid, Basic Psychology II<br>Ochoa-Urrea, M; University of Texas McGovern Medical School, Department of Neurology<br>Funke, ME; University of Texas McGovern Medical School, Department of Pediatrics<br>Hasan, O; University of Texas McGovern Medical School, Department of Neurology<br>Zhu, P; University of Texas McGovern Medical School, Vivian L. Smith Department of Neurosurgery; The University of Texas Health Science Center at Houston, Texas Institute for Restorative Neurotechnologies<br>Marcos Dolado, Alberto; Hospital Clínico Universitario San Carlos, Neurology<br>López, Maria; Universidad Politecnica de Madrid, Center for Biomedical Technology; Universidad Complutense de Madrid, Basic Psychology II<br>Schulz, Paul; University of Texas McGovern Medical School, Department of Neurology<br>Lhatoo, S; University of Texas McGovern Medical School, Department of Neurology<br>Pantazis, D; Massachusetts Institute of Technology McGovern Institute for Brain Research<br>Mosher, JC; University of Texas McGovern Medical School, Department of Pediatrics<br>Maestú, Fernando; Universidad Politecnica de Madrid, Centre for Biomedical Technology; Universidad Complutense de Madrid, Basic Psychology II
Keywords:	MCI, Epilepsy, Functional Connectivity, MEG, Gamma

SCHOLARONE™ Manuscripts

Gamma band functional connectivity reduction in patients with amnestic mild cognitive impairment and epileptiform activity

Cuesta P[1,4], Ochoa-Urrea M[2,6], Funke ME[3], Hasan O[2], Zhu P[5,6], Marcos A[4], López ME[1], Schulz PE[2], Lhatoo S[2,6], Pantazis D[7], Mosher JC[2,6], Maestu F[1,3]

[1] Department of Experimental Psychology, Cognitive Processes and Speech Therapy, Complutense University of Madrid, Madrid, Spain

[2] Department of Neurology, McGovern Medical School, The University of Texas Health Science Center at Houston, Houston, Texas, USA

[3] Department of Pediatrics, McGovern Medical School, The University of Texas Health Science Center at Houston, Houston, Texas, USA

[4] Department of Radiology, Rehabilitation and Physiotherapy, Complutense University of Madrid, Madrid, Spain

[5] Vivian L. Smith Department of Neurosurgery, McGovern Medical School, The University of Texas Health Science Center at Houston, Houston, Texas, USA

[6] Texas Institute for Restorative Neurotechnologies, University of Texas Health Science Center at Houston

[7] McGovern Institute for Brain Research, Massachusetts Institute of Technology, Cambridge, USA

Correspondence:

For Review Only

Abstract

There is growing evidence in recent years of neuronal hyperexcitability in Alzheimer's disease (AD). Hyperexcitability is associated with an increase in epileptiform activity (EA) and the disruption of inhibitory activity of interneurons. Interneurons fire at a high rate and are frequently associated with high-frequency oscillations and are associated with the gamma frequency band (30–150Hz). It is unclear how hyperexcitability affects the organization of functional brain networks. In a sample of 63 amnestic mild cognitive impairment (MCI) patients underwent a Magnetoencephalography (MEG) resting-state recording with eyes closed. Twenty (31.75%) MCI patients presented with EA. A cluster-based analysis of the MEG functional connectivity revealed a region within the right temporal cortex whose global connectivity in the gamma frequency band was significantly reduced in patients with EA relative to those without EA. A subsequent seed-based analysis showed this was largely due to weaker gamma-band connectivity of this region with ipsilateral frontal and medial regions, and the upper precuneus area. In addition, this reduced functional connectivity was associated with higher gray matter atrophy across several cortical regions in the patients with EA. These functional network disruptions and changes in brain physiology and morphology have important clinical implications as they may contribute to cognitive decline in MCI and AD.

For Review Only

Introduction

Cognitive decline in the development of Alzheimer's Disease (AD) has been associated with progressive brain atrophy and the accumulation of hyperphosphorylated tau and amyloid proteins (Cummings et al., 1998). A functional neuronal network is supported by a fine balance between neuronal excitation and inhibition (E/I) and a disruption in this balance may lead to alterations in network organization. Additionally, network disruptions can affect signal transmission and a breakdown of interareal communication.

In the last decade, there has been growing evidence for hyperexcitability in AD patients. Compared to the general population, AD patients are 8 to 10 times more likely to develop spontaneous seizures (Hauser et al., 1986; Scarmeas et al., 2009). Cortical hyperexcitability in AD patients could reflect a disruption of the E/I balance. Animal models of AD have shown increased neuronal firing in the vicinity of amyloid plaques (Busche and Konnerth, 2016). Studies in humans have demonstrated that amyloid toxicity caused a loss of inhibitory terminals (Garcia-Marin et al., 2009). Finally, increased epileptiform activity (EA) has been found in brain regions typically affected by the neuropathology of AD (Vossel et al., 2013; Vossel KA et al., 2016; Brunetti et al., 2020; Lam et al., 2020).

Hyperexcitability could lead to increased neuronal synchronization and a dysfunctional organization of the profiles of brain activity shown at multiple frequency bands with electroencephalography (EEG) and magnetoencephalography (MEG). Increased phase synchrony between the anterior and posterior regions has been found in mid-adult humans with amyloid deposition (Nakamura et al., 2017) and in relatives of AD patients (Ramírez-Toraño et al., 2020), all at preclinical stages. Furthermore, this neurophysiological signature was also found in elders with subjective cognitive decline (López-Sanz et al., 2017) and in Mild Cognitive Impairment (MCI) patients who later progressed to dementia (López et al., 2016; Pusil et al., 2019).

Palop and Mucke noted a link between cognitive impairment and disruption of interneuron inhibitory activity in a comprehensive review in 2016 (Palop and Mucke, 2016). In addition, the firing of interneurons is more prominent and synchronized with high-frequency oscillations, such as those in the gamma band (30–150 Hz). The gamma band has been frequently associated with local and long-distance communication (Rouhinen et al., 2020) and with memory function by predicting those items that will successfully be recalled later (Sederberg et al., 2007). A reduction of the gamma band power has been associated with the appearance of epileptic discharges in an animal model of AD (Maheshwari et al., 2016). Furthermore, patients with epilepsy tend to show higher gamma band activity during successful encoding of words (Matsumoto et al., 2013). Thus, extensive evidence now directly links the gamma band, memory formation, and interneuron modulatory activity.

The goal of this study was to test whether hyperexcitability in the form of EA found in MCI patients induces alterations of crucial oscillatory activity associated with memory formation. We hypothesized that there would be greater network dysfunction in MCI with EA.

For Review Only

Materials and Methods

Subjects

Sixty-three amnestic MCI patients were recruited from the Hospital Universitario San Carlos (Madrid, Spain) (Maestú *et al.*, 2015). All were native Spanish speakers and right-handed. The MCI diagnosis was established according to the NIA-AA clinical criteria (Albert et al., 2011), which include (i) self- or informant- reported cognitive complaints, (ii) objective evidence of impairment in one or more cognitive domains, (iii) preserved independence in functional abilities, and (iv) not demented (McKhann et al., 2011). For more information about the diagnostic criteria for MCI see (López et al., 2020). None of the participants exhibited a history of psychiatric or neurological disorders other than MCI. General inclusion criteria were as follows: age between 60 and 90 years, a modified Hachinski score ≤ 4, a short form Geriatric Depression Scale score ≤ 5, and a T1/T2-weighted MRI within 54 weeks before the MEG recordings (on average, the time period between the MEG and MRI recordings was 3 months) without an indication of infection, infarction, or focal lesions (rated by two independent experienced radiologists (Bai et al., 2012)). In addition, we advised subjects to avoid medications that could affect MEG activity, such as benzodiazepines, for 48 hours before recordings. All participants provided written, informed consent. The Institutional Review Board Ethics Committee at Hospital Universitario San Carlos approved the study protocol, and the procedure was performed following the Helsinki Declaration and National and European Union regulations.

MRI acquisition and volumetric analyses

T1-weighted MRI images from each participant were acquired with a General Electric 1.5 T MRI scanner using a high-resolution antenna and a homogenization PURE filter (Fast Spoiled Gradient Echo sequence, TR/TE/TI = 11.2/4.2/450 ms; flip angle 12°; 1 mm slice thickness, 256x256 matrix and FOV 25 cm). The resulting images were processed using Freesurfer software (version 5.1.0) and its specialized tool for automated cortical parcellation and subcortical segmentation (Fischl, 2012).

MEG recordings and interpretation

A scheme of the methodological pipeline can be seen in Figure 1. MEG signals were acquired using a whole-head Elekta-Neuromag MEG system with 306 channels (Elekta AB, Stockholm, Sweden) at the Center for Biomedical Technology (Madrid, Spain). Data was collected at a sampling frequency of 1000 Hz and online band-pass filtered between 0.1 and 330 Hz. The MEG protocol consisted of 5-minute resting state eyes-closed, 5-minutes resting state eyes-open and 10-minutes of passive face viewing while sitting comfortably inside a magnetically shielded room. For functional connectivity analysis, we used the 5 minutes of resting state eyes closed recordings. For epileptiform activity screening, we visually inspected all three recordings comprising approximately 20 minutes. Participants were asked to stay awake and to minimize their body movements. Each participant's head shape was defined relative to three anatomical locations (nasion and bilateral preauricular points) using a 3D digitizer (Fastrak, Polhemus, VT, USA) and head motion was tracked through four head-position indicator (HPI) coils attached to the scalp. These HPI coils continuously monitored the subjects' head movements, while eye movements were monitored by a vertical electro-oculogram (EOG) assembly composed of a pair of bipolar electrodes. Raw data was first processed with Maxfilter software (v 2.2, temporal signal-space separation [tSSS], correlation threshold =

For Review Only

0.9, time window = 10 seconds) to remove external noise using the temporal extension of the signal space separation method with movement compensation (Taulu and Simola, 2006). Data underwent automatic artifact selection using FieldTrip (Oostenveld et al., 2011), and a MEG expert confirmed the findings. After the artifact's removal, we applied second-order, blind identification (SOBI) (Belouchrani et al., 1997) to remove artifacts from ECG, EOG, and other noise related interferences. Only the magnetometers' data were used for subsequent analysis since the sensor-space data is highly redundant after Maxfilter processing (Garcés et al., 2017). The remaining artefact-free data were partitioned into 4-s segments (epochs). Only those recordings with at least 20 clean segments (80 seconds of brain activity) were included in subsequent analyses. Prior to source estimation, the MEG time courses were filtered into theta (4.1-7.9 Hz), alpha (8.1-11.9 Hz), beta (12.1-29.9 Hz), and gamma (30.1-45.0 Hz) frequency bands with a 1500 order finite impulse response filter using a Hamming window and a two-pass filtering procedure.

A MEG expert (MEF) and a neurologist trained in MEG reading (MOU) screened MEG signals for EA (see Figure 2). Both reviewers inspected the data together and decisions on the presence of EA discharges were taken by consensus. In case of disagreement, resolution relied on the opinion of the most experienced neurophysiologist (MEF). No additional opinion from a third reader was obtained. Localization of the spikes were obtained using single dipole modeling, which is the accepted approach in clinical MEG (see CPG 1, (Bagić et al., 2011). Raw data was reviewed after Maxfilter processing. We defined EA as a transient signal that was clearly distinguished from background activity, and with a pointed peak component (Fernandes et al., 2005; Nowak et al., 2009). In order to be valid, only statistically significant sources (reduced Chi square ≥ 1 and ≤ 2, confidence volume <1000mm^3, source strength between 100-500 nanoamperes, goodness of fit >75%) were accepted. Source localization of the dipoles was performed in the DANA Elekta Neuromag Software (Elekta AB, Stockholm, Sweden) superimposing the dipoles on the patient's structural MRI. Depending on the presence or absence of EA, patients were divided into two groups: MCI EA+ and MCI EA-, comprising patients with and without EA, respectively.

For Review Only

Source Reconstruction and Connectivity Analysis

The geometry of the MEG source space was modeled with a regular volumetric grid with 10 mm spacing created in the template MNI brain. This set of nodes was transformed to each participant's space using a non-linear normalization between the native T1 image (whose coordinate system was previously converted to match the MEG coordinate system) and a standard T1 image in MNI space. The forward model solution used a single-shell method (Nolte, 2003) with a unique boundary defined by the inner skull (the combination of white matter, grey matter and CSF) extracted from each individual T1 image. Source reconstruction was carried out independently for each subject and frequency band with a linearly constrained minimum variance (LCMV) beamformer (Van Veen et al., 1997), using the epochs-average covariance matrix and a regularization factor of 5% of the average sensor power. This method has yielded reliable results for the estimation of resting-state functional connectivity (Hincapié et al., 2017). Each source position was labeled using the Automated Anatomical Labeling (AAL) atlas (Tzourio-Mazoyer et al., 2002). Only those sources labeled as part of one of the 78 cortical areas of the atlas were included in subsequent analyses (1202 nodes in total). Functional connectivity (FC) between these 1202 nodes was assessed with phase locking value (PLV), a phase synchronization measure that evaluates the distribution of phase differences extracted from two ROIs time series (Mormann et al., 2000) and has high reliability

across sessions (Garces et al., 2016). Symmetrical, whole-brain matrices of 1202x1202 nodes were thus obtained by averaging PLV values across epochs for each participant and frequency band. Lastly, we computed the strength of each node (also known as weighted global connectivity), which is defined as the sum of its FC with the rest of the nodes. To account for the number of links, the strength of each node was then normalized by dividing the number of links connected to it. This procedure resulted in one brain map of normalized node strengths per each participant and frequency band.

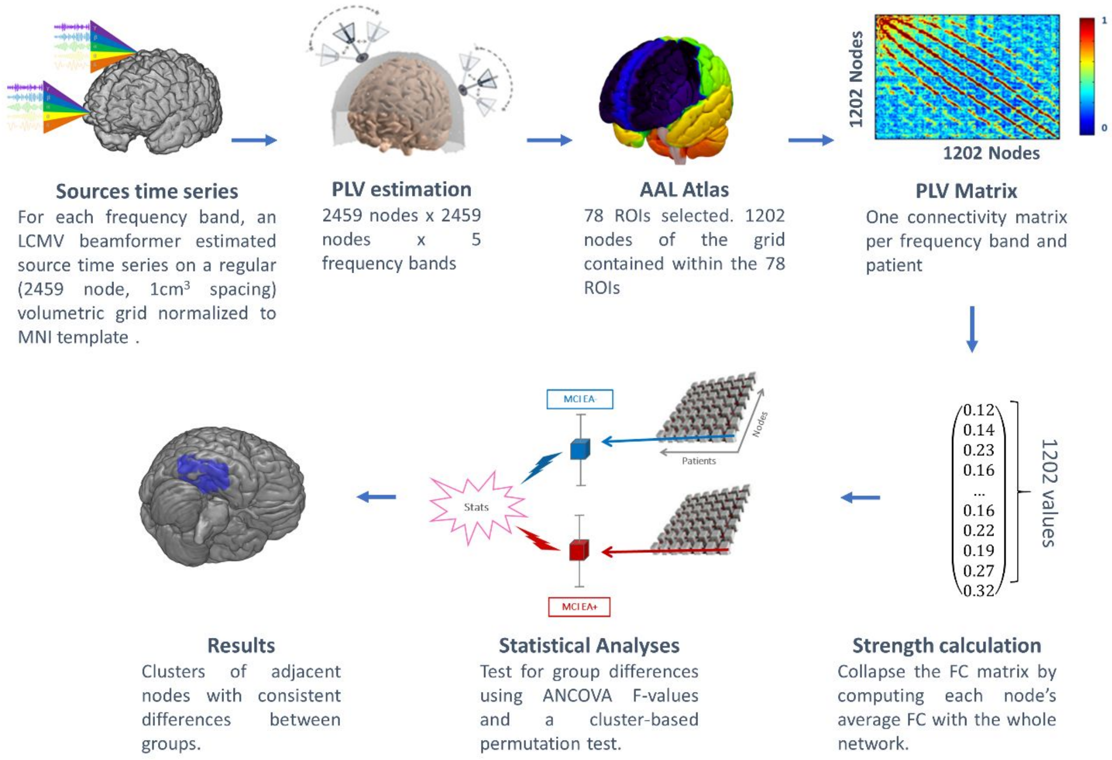

Figure 1. *Schematic diagram of processing pipeline. For each frequency band, an LCMV beamformer estimated source time series in a regular volumetric grid of nodes. Phase locking values (PLV) were then estimated between every pair of nodes contained within one of 78 ROIs from the AAL atlas. These values were used to compute the normalized strength of each node, defined as the sum of its PLV with the rest of the nodes, divided by the number of connected nodes. Last, the strength values were subjected to statistical analyses using ANCOVA and a cluster-based permutation test to identify clusters of adjacent nodes with significant functional connectivity differences between the MCI EA+ and MCI EA- groups.*

Statistical analyses

The assessment of significant group FC differences was based on a cluster-based permutation test (CBPT) described previously (Maris and Oostenveld, 2007; Zalesky *et al.*, 2010) where the units of study were clusters of spatially adjacent nodes whose strength (weighted global connectivity) differed significantly between groups with the same sign. This procedure was applied independently for each frequency band, as implemented in Fieldtrip (Oostenveld et al., 2011). The methodology started by testing each of the 1202 nodes separately for strength differences between the two groups using an ANCOVA test

while adjusting for the effects of age. This procedure yielded one F-statistic value per node and resulted in a volumetric map of 1202 F-statistic values. This F-statistic map was thresholded using a critical value corresponding to the 0.005 significance level for the F-statistic (cluster-defining threshold). Subsequently, the thresholded map was split into two maps corresponding to the voxels with connectivity MCI EA+ > MCI EA- or MCI EA+ < MCI EA-. For each map, a clustering procedure identified groups of adjacent nodes in the volume space, and the mass of each cluster was defined as the sum of the F-values of the nodes comprising each cluster (cluster mass statistic). We employed this measure because it reflects a combination of the topological extend (number of nodes) and the effect size (F-values). As an inclusion criterion to suppress spurious findings, we required the minimum size for each candidate cluster to be equal to 1% of the total nodes in the volume, and all cluster smaller than this size were automatically deemed non-significant.

Then, to control for multiple comparisons, this entire procedure was repeated 5000 times after randomly shuffling the original group's labels and creating permutation samples of the original F-statistic maps. At each repetition, the maximum cluster mass statistic of the surrogate clusters was stored, thus constructing an empirical distribution of the maximum cluster mass statistic. This maximal null distribution enables us to compute the p-value for each candidate cluster of the original data and thus the control of the family-wise error rate (FWER) at the cluster level. Only those clusters that survived the CBPT at $p < 0.05$ were considered for the subsequent analyses as potential MEG markers. As a representative value of these MEG markers, we computed the average strength of the nodes contained in the cluster. These values are shown in the boxplots of Figure 3 and were tested for differences between both groups with further ANCOVA test with age as covariate. These values were also used in a subsequent Spearman correlation analysis with cognitive and structural scores.

The significant clusters detected with the above procedure differed in global connectivity between the two groups. As a post-hoc analysis, we applied a seed-based procedure to determine whether the strength differences (i.e., the global FC of the cluster) were primarily driven by the existence of a few isolated connections rather than caused by a "global/widespread" effect (widespread connections of each original cluster to the rest of the brain). The seed region was calculated by taking all nodes that were in a radius of 20 mm from the physical mass center of the cluster. Then, we computed all 1202 FC values between each node and the cluster's mass center (seed), yielding a seed connectivity map. This map was subjected to the same clustering procedure described above. Only clusters that did not overlap with the original cluster were reported in this study to ensure reliable results that did not depend on the precise extent of the original cluster. Statistical analyses were carried out using Matlab R2020b (Mathworks Inc) and all tests were two-tailed.

Data availability

The data that support the findings of this study are available from the corresponding author, upon request. All the algorithms used in the present paper are reported in the 'Materials and methods' section.

For Review Only

Results

Of the 63 patients in the cohort, MEG was positive for EA in 20 patients (31.75%). The average number of spikes in the patients that showed epileptiform activity was 2.04 ± 1.79. Most of the spikes detected in MCI patients, were in broad regions encompassing temporal (58%) and frontal (24%) areas. None of the subjects showed a clear cluster of EA, nor had seizures. Examples of EA discharges for two patients are shown in Figure 2.

For Review Only

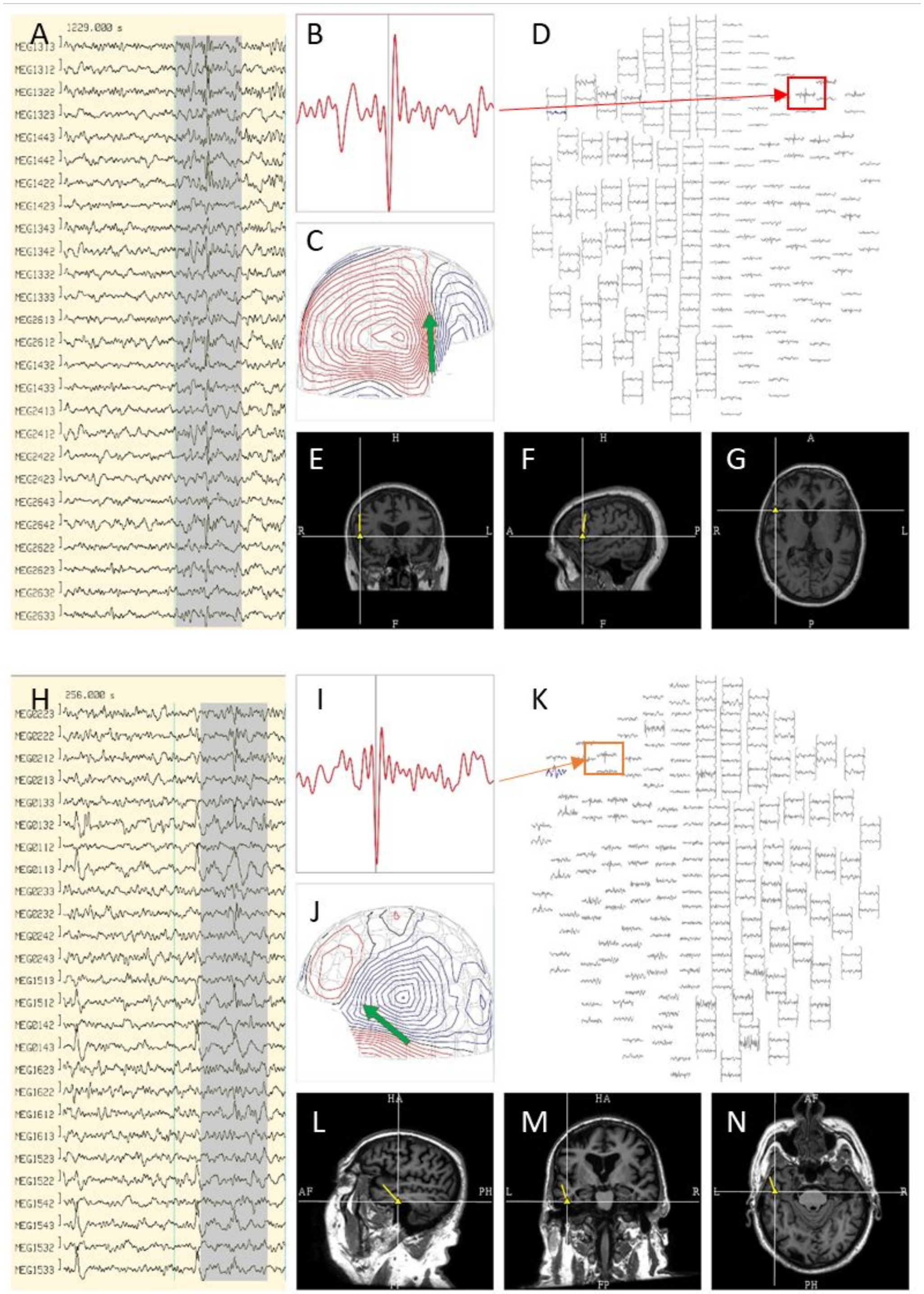

Figure 2. Upper panel: (A) An example of an individual MEG discharge in the right frontal area. (B) Selected MEG channel and time instance of the magnetic field distribution (C) in sensor space with the projected source estimate (green arrow). The MEG channel plot (D) of the selected time interval as shown in (A) shows the planar gradiometer channel (red square and arrow) with the earliest peak time. Figures E-G represent the dipole (yellow triangle) and its orientation (yellow tail) arising from the frontal opercular region in the coronal (E), sagittal (F) and axial (G) views. Lower panel: (H) An example of an individual MEG discharge in the left temporal area. (I) Selected MEG channel and time instance of the magnetic field distribution (J) in sensor space with the projected source estimate (green

arrow). The MEG channel plot (K) of the selected time interval as shown in (H) shows the planar gradiometer channel (orange square and arrow) with the earliest peak time. Figures L-N represent the dipole (yellow triangle) and its orientation (yellow tail) originating from anterior basal temporal structures in the sagittal (L), coronal (M) and axial (N) views.

The demographics, genetics, clinical scores, and brain volumetric data information at baseline evaluation for the Madrid Cohort are shown in Table 1. There were no statistically significant differences between the patients with (MCI EA+) and without (MCI EA-) epileptiform activity in any of the comparisons.

	MCI EA+ (n=20)		MCI EA- (n=43)		
	Mean	Sd	Mean	Sd	p-value
Age (years)	74.35	5.33	74.23	5.36	0.9356#
Gender (females)	12		24		0.7912$
APOE 4 genotype (%)	6		22		0.1581$
Education (years)	8.24	3.87	8.62	4.83	0.7719#
Geriatric depression scale	3.79	2.94	3.69	2.98	0.9227#
MMSE score	25.44	2.57	25.63	2.74	0.8141#
Immediate recall	15.22	7.46	13.86	10.41	0.6173#
Delayed recall	4.50	5.87	4.75	7.99	0.9058#
Forward digits	7.33	1.68	6.57	2.11	0.1800#
Backward digits	4.28	1.13	4.19	1.61	0.8355#
Left hippocampal volume	0.0022	0.0005	0.0022	0.0004	0.9289#
Right hippocampal volume	0.0022	0.0004	0.0022	0.0003	0.7858#
Total grey matter volume	512690	61353	527175	51912	0.3444#
Total cerebral white matter	400070	80787	392009	58289	0.6617#

Table 1. Demographics, genetics, clinical scores, and brain volumetric information of the 63 MCI patients indicating that the two groups are similar with regard to age, ApoE 4 genotyping, cognitive status, and selected gray matter volumes. **MCI**: mild cognitive impairment patients. **EA, +-**: existence or not of epileptiform activity, **$**: Fisher's exact test, **#**: t-tests. **MMSE**: Mini Mental State Examination. Total grey matter and whiter matter volumes are in mm^3. Volumes of anatomical structures are normalized by intracranial volume.

Next, we analyzed the MEG functional networks to identify brain regions with global connectivity differences between the MCI EA+ and MCI EA- groups. We found one significant cluster (CBPT; cluster mass statistic = 268.14, p-value = 0.0160) in the gamma band (henceforth referred to as "primary"), largely focused on the right temporal region (mass center [49 -32 -22] mm, MNI coordinates) of the brain (see Figure 3A and Table 2, column 1). Comparing groups, the MCI EA+ cluster had significantly reduced normalized global connectivity relative to the MCI EA- group. We computed the average strength of the nodes contained in the cluster as a surrogate effect size and carried out a new ANCOVA Test with age to

For Review Only

quantify it. The values obtained of the differences at the cluster level were p value < 0.001 and F-statistic 19.4. This result indicates that the oscillatory activity (within the gamma frequency band) of that cluster was less synchronously paired with activity from across the brain.

In order to identify specific connections that drove the global connectivity change of the primary cluster, we performed a subsequent seed-based analysis. This analysis identified the specific regions across the rest of the brain (secondary clusters) that showed significant between-group FC differences with the primary cluster. We found two secondary clusters where the FC with the original cluster was significant decreased in the MCI EA+ group as compared to the MCI EA- group. The first (referred to as "secondary-1") involved mainly ipsilateral frontal and medial regions (Figure 3B & Table 2, column 2). The average strength of the FC between the primary cluster and the secondary-1 differed between groups with the following scores p value < 0.001 and F-statistic 25.6. The second (referred to as "secondary-2") was found in the upper precuneus area (bilateral) (Figure 3C & Table 2, column 3). In this case, the differences between groups for the average strength of the FC between the primary cluster and the secondary-2 showed a p value < 0.001 and F-statistic 15.5.

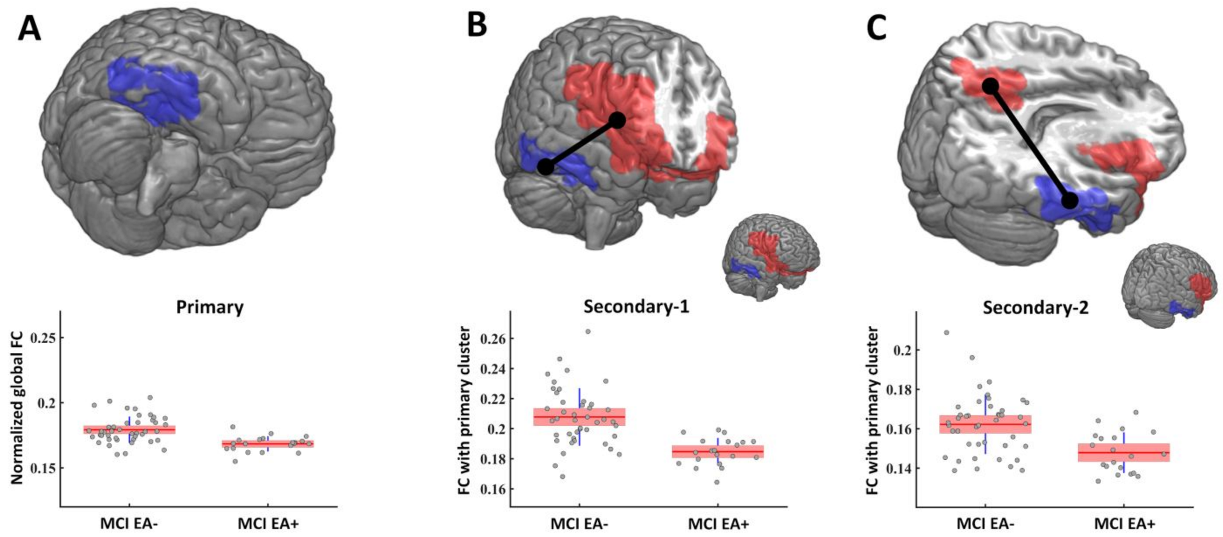

Figure 3. Significant MCI EA+ and MCI EA- network differences in the gamma band. (A) Dark blue region in the right temporal lobe had significantly decreased gamma band global connectivity in the MCI EA+ group (cluster named primary). (B, C) Red regions, marked as secondary-1 and secondary-2, have FC with the primary cluster significantly decreased in the MCI EA+ compared to the MCI EA- group. Black lines in B and C represent the significant FC link between the primary and the secondary clusters. Boxplots describe the FC of the corresponding cluster for each group, with dots representing individual patients.

Primary cluster			Secondary-1 cluster			Secondary-2 cluster		
ROI name	%	F#	ROI name	%	F#	ROI name	%	F#
rITG	54	11,9	rIFGor	77	15,4	lMCC	19	11,8
rFusiG	42	11,6	rPosG	18	15,0	lPCC	80	11,2
rMTG	3	9,3	rRO	64	14,8	lPrecu	14	10,9
			rSTG	4	14,5	rPrecu	5	9,7
			rRectus	25	14,1	lMOccL	3	9,7
			rPreCG	11	13,1	lSPG	6	9,3
			rIFGt	63	12,6			
			rSFo	33	12,3			
			rInsula	57	11,3			
			rTPmid	10	11,2			
			lInsula	36	11,1			
			lAmyg	50	10,9			
			rIFGo	58	10,8			
			lMFGo	14	10,0			
			rMFG	3	10,0			
			rTPsup	50	9,9			
			lIFGt	33	9,9			
			lIFGo	14	9,7			
			lParahip	13	9,7			
			lMFG	3	9,6			
			lIFGo	58	9,5			
			lRectus	25	9,2			

Table 2. Regions of interest (ROIs) from the AAL atlas that comprise each significant cluster. %: percentage of the ROI within the cluster. #: sum of all F values obtained at the node level. ROIs were ordered based on their significance (F column). r/l=right/left. ITG: inferior temporal gyrus; FusiG: fusiform gyrus; MTG: middle temporal gyrus; PCC: posterior cingulate gyrus; Precu: precuneus; MCC: middle cingulate gyrus; MOccL: middle occipital lobe; SPG: superior parietal gyrus; IFGt: inferior frontal gyrus triangular; IFGor: inferior frontal gyrus opercular; IFGo: inferior frontal gyrus orbital; SFGo: superior frontal gyrus orbital; RO: rolandic operculum; PosCG: postcentral gyrus; TPsup: temporal pole, superior temporal gyrus; PreCG: precentral gyrus; SFo: superior frontal gyrus orbital; MFG: middle frontal gyrus; MFGo: middle frontal gyrus orbital; Amyg: amygdala; Parahip: parahippocampus; STG: superior temporal gyrus; TPmid: temporal pole, middle temporal gyrus.

For Review Only

To establish a critical link between the aberrant FC of the above clusters and scores of brain health (neurophysiological assessment and structural quantitative scores associated with grey matter atrophy), we conducted Spearman correlation analyses between these measures. For functional values, this analysis used the normalized global connectivity of the primary cluster or the FC between <primary, secondary-1> and <primary, secondary-2>. For neurophysiological scores, we used those described in Table 1. For grey matter scores, we used those of regions contained within the significant clusters. This analysis did not yield any significant between-group differences in any correlation between the FC and the brain health scores.

Next, we conducted a similar correlation analysis, but within each group separately. This analysis yielded significant effects in the comparisons involving the FC of the primary cluster with the secondary-2 cluster in the MCI EA+ group. Specifically, the <primary, secondary-2> FC values were positively correlated with several markers of gray matter volume in the MCI EA+ group (Table 3). This result suggests that reduced FC between these two clusters is associated with higher gray matter atrophy across several

brain regions in the MCI EA+ patients. We did not find significant effects for any group in all other comparisons involving the global connectivity of the primary cluster, or the FC of the primary with the secondary-1 cluster.

MCI EA+		
Structure	***r***	***p value****
l GM lateral orbitofrontal	0,618	0,004
l GM medial orbitofrontal	0,639	0,003
l GM pars opercularis	0,660	0,002
l GM pars orbitalis	0,553	0,013
l GM posterior cingulate	0,594	0,007
r GM lateral orbitofrontal	0,580	0,008
r GM medial orbitofrontal	0,626	0,004
r GM precuneus	0,644	0,003

Table 3. Spearman correlation analyses between the FC of the <primary, secondary-2> clusters and brain structural integrity scores for the regions contained within the significant clusters. **l/r**, left/right. **GM**, grey matter. * p values remained significant after FDR (q = 0.05) correction.

For Review Only

Discussion

We evaluated MEG functional networks in MCI patients with and without EA to test whether crucial frequency bands, previously associated with memory functioning, were disrupted. If so, this would contribute to a better understanding of cognitive decline in this stage of the AD continuum. MCI patients with EA showed decreased gamma band connectivity in comparison with MCI patients without EA. The brain regions identified in this reduced gamma network involved right temporal regions, ipsilateral dorsolateral and medial frontal regions, and upper precuneus area. These areas and behavior, decreased FC in the epileptic patients, have been found in previous studies involving non elders with epilepsy (Englot et al., 2015) and they have been associated with the appearance of neurocognitive problems, including memory and language impairments (Englot et al., 2016). In fact, these regions are typically associated with executive functions and episodic memory in healthy subjects, and with cognitive impairment in patients with brain lesions and different types of neurological disorders (Ferguson et al., 2019).

The gamma band has been associated with episodic memory function (Lin et al., 2019) and has been found to predict successful or unsuccessful recovery (Tan et al., 2020). Furthermore, patients with epilepsy showed a reduction of gamma band power associated with EA in the hippocampal area during an episodic memory task (Lega et al., 2015). Interestingly, these gamma band effects were found in a very similar network as the one described here.

EA in patients with AD (including patients with MCI) has been associated with a faster decline in global cognition and executive functions (Vossel KA et al., 2016) as well as with a higher percentage of conversion from MCI to dementia (Vossel et al., 2017). This is consistent with our findings here that there were localized, functional connectivity disruptions in the brain regions involved in the episodic memory network.

Aberrant functional connectivity of this network could indicate a higher risk of compromised neurophysiological mechanisms that support memory function. Past work has shown that EA may induce transient cognitive impairment, indicating how this phenomenon interferes with cognitive processing (Ung *et al.*, 2017). Since episodic memory decline is one of the initial symptoms of AD, the localization of EA at the medial temporal lobe regions could be associated with cognitive impairment. However, the scarce incidence, likely influenced for having only 20 minutes long recordings, and heterogeneity of localization of EA in our cohort of MCI patients make it difficult to statistically assess this hypothesis.

It is important to discuss whether the reduction of functional connectivity in the group of MCI EA+ patients was caused by the epileptiform activity epileptiform activity itself or to the malfunction of the memory networks. In epilepsy, electrophysiological data tend to show an increased high gamma frequency (30-100Hz) associated with epileptiform activity (Ren *et al.*, 2015; von Ellenrieder *et al.*, 2016; Rampp *et al.*, 2021). This seems to be counterintuitive with our findings assessing MCI patients in the current study. However, there are some critical differences between those studies and ours. Whilst the cited papers assessed epileptic patients, the presence of epileptiform activity in our MCI EA+ patients were scarce (patients did not have seizures), indicating that EA itself does not sustain the network malfunction. This fact is important because the neuropathological findings in epilepsy may differ from those typically found along the AD continuum. Our data consisted of non-invasive MEG recordings, whilst (Ren et al., 2015; Rampp et al., 2021) were carried out using intracranial EEG. The results from these two methodologies are difficult to interpret together since the local effects found with intracranial EEG are quite different to the macroscopic signals assessed with non-invasive EEG/MEG. Besides the differences in the data and technique, there are some methodological differences such as the different gamma band definitions. We focused on the classical gamma band (30-45 Hz) rather than the broader definitions used in the referenced studies. Given the differences in technique and methodology, our study offers new and different information in the underlying processes that occur in tandem with the development of AD neuropathology. Therefore, our findings of aberrant functional connectivity in MCI patients are more consistent with a disruption of the episodic memory networks. This disruption cannot be explained by the epileptogenic activity alone. In fact, we hypothesize that abnormal network functioning is a risk factor for EA, and hence that MCI EA+ patients might be at higher risk of conversion to dementia. EA potentially contributes to network decline.

We cannot rule out the possibility of a locally produced increase in gamma band frequency oscillations, generated close to the EA. In the context of epilepsy, EA may induce more random phases that cause a reduction in long-distance synchronization. The gamma band hyposynchronized network involves inferior frontal, temporal, and inferior parietal regions. While the spikes found in this population of MCI participants were located essentially in frontal and temporal lobes, they were distributed in broader areas encompassing dorsal aspects of the temporal and frontal regions. Furthermore, the incidence of EA was low, suggesting that this activity did not drive the macroscopic gamma band FC depletion. It is possible, then that this gamma band network disruption reflects the episodic memory dysfunction rather than the EA itself. Nonetheless, it appears that EA is associated with the disruption of this network.

The correlational analyses with grey matter volumes reinforce our that our results are consistent with a disruption of memory networks. The MCI EA+ patients showed a direct relationship between brain atrophy and reduction of the γ-band connectivity between the middle temporal gyrus and the prefrontal/parietal regions. This indicates that the functional reduction of the gamma band connectivity is accompanied by orbitofrontal, precuneus, and posterior cingulate cortex volume reduction. These brain regions have been

For Review Only

typically associated with the episodic memory network (Ferguson et al., 2019). Neurodegeneration is one of the key features of the course of the disease, forming one of the core elements of the ANT axis (Jack et al., 2017). Therefore, it seems logical that the loss of grey matter volume may affect the functional connections within the episodic memory network. This network (orbitofrontal, cingulate cortex, precuneus and the hippocampus) is densely interconnected by the cingulum bundle, which connects with the callosal splenium and trough there with precuneus and prefrontal regions (Bubb et al., 2018). Therefore, the morphological alterations of these regions could cause a depletion of the functional connectivity in the temporal lobe at a specific frequency associated with memory formation. Although we did not find differences in brain atrophy between groups, the correlation analysis revealed important associations with the functional connections pointing to an anatomo-functional dysfunction in the MCI EA+ group, suggesting that the higher the FC, the better the grey matter integrity in these patients.

There were no differences between groups in cognitive performance, nor in brain structural integrity. The only difference between groups was found for the FC assessment. However, the fact that only the EA+ group showed significant correlations with brain structural integrity might indicate that the appearance of EA could reflect alterations at the structural level. This relationship between function and structure was not found in the EA- group. The precedence in bran resting state of functional abnormalities to any structural or cognitive damage has been stated in previous studies (Nakamura *et al.*, 2017, 2018). In this study, the reported gamma FC pattern is different from the typical electrophysiological "slowing" effect usually found in AD. This fact suggests that the gamma depletion could be an epiphenomenon of network disruption associated with new neuropathological pathways that could accelerate the neuronal damage induced by the dementia progression.

For Review Only

This study has some limitations. The MEG recordings were acquired during a resting state condition, but most of the studies referenced in our discussion found gamma-band episodic memory effects during memory tasks and not at rest. While this prevents a direct comparison of our findings with previous literature, it is important to highlight that many task-related networks have also been found at rest, such as the sensorimotor and social networks (Biswal et al., 1995; Yeshurun et al., 2021). Furthermore, there is strong evidence that associates the default mode network with episodic memory functions (Sestieri et al., 2011) indicating that brain regions associated with task performance are also engaged in some default mode functions crucial to memory reorganization and engram maintenance. It is important to note that our MCI sample did not have any AD neuropathological marker (tau or amyloid). Our patients met the MCI clinical criteria of the NIA-AA and have neurodegeneration biomarkers (i.e., hippocampal grey matter measures) but future studies including tau or amyloid information will be key to understand how AD-specific the network disruption may be. Another important limitation is the low incidence of EA in our MCI EA+ patients and its heterogeneous localization pattern. The low incidence could be partially underestimated due to the limited amount of data. Longer and more epilepsy-specific recordings could have allowed both the detection of more MCI EA+ patients, and a better characterization of the EA in the MCI EA+ patients. Finally, to identify and exclude MEG-unique normal variants, EEG is needed. Our preliminary data were recorded without simultaneous EEG. Hence there is a possibility that source heterogeneity is inflated.

Our findings link EA, gamma-band functional disruptions, and alterations contained within the memory network in our MCI patients. Moreover, they demonstrate the importance of all these factors for a better understanding of memory decline in the early stages of AD. Future studies with increased sample size, more sensitive memory tasks, and longer brain recordings that are optimal for the detection and characterization

of EA activity, could extend our findings and assess the potential influence of other occult factors that may have an important role in memory decline and the onset of epileptiform activity.

Funding

This study was supported by two projects (PSI2009-14415-C03-01 and PSI2012-38375-C03-01) and by a postdoctoral fellowship to Pablo Cuesta (IJC2018-038404-I) from the Spanish Ministry of Economy and Competitiveness.

Compliance with ethical standards

The Hospital Universitario San Carlos Ethics Committee (Madrid) approved the study, and all participants or their caregivers signed a written informed consent prior to participation.

Conflict of interest

The authors declare that they have no competing interests.

For Review Only

References

Albert MS, DeKosky ST, Dickson D, Dubois B, Feldman HH, Fox NC, et al. The diagnosis of mild cognitive impairment due to Alzheimer's disease: recommendations from the National Institute on Aging-Alzheimer's Association workgroups on diagnostic guidelines for Alzheimer's disease. Alzheimers Dement 2011; 7: 270–9.

Bagić AI, Knowlton RC, Rose DF, Ebersole JS. American clinical magnetoencephalography society clinical practice guideline 1: Recording and analysis of spontaneous cerebral activity. J Clin Neurophysiol 2011; 28: 348–54.

Bai Y, Hu Y, Wu Y, Zhu Y, He Q, Jiang C, et al. A prospective, randomized, single-blinded trial on the effect of early rehabilitation on daily activities and motor function of patients with hemorrhagic stroke. J Clin Neurosci 2012; 19: 1376–9.

Belouchrani A, Abed-Meraim K, Cardoso JF, Moulines E, -. Cardoso J, Moulines E. A blind source separation technique using second-order statistics. IEEE Trans Signal Process 1997; 45: 434–44.

Biswal B, Yetkin FZ, Haughton VM, Hyde JS. Functional connectivity in the motor cortex of resting human brain using echo-planar MRI. Magn Reson Med 1995; 34: 537–41.

Brunetti V, D'Atri A, Della Marca G, Vollono C, Marra C, Vita MG, et al. Subclinical epileptiform activity during sleep in Alzheimer's disease and mild cognitive impairment. Clin Neurophysiol 2020; 131: 1011–8.

Bubb EJ, Metzler-Baddeley C, Aggleton JP. The cingulum bundle: Anatomy, function, and dysfunction. Neurosci Biobehav Rev 2018; 92: 104–27.

Busche MA, Konnerth A. Impairments of neural circuit function in Alzheimer's disease. Philos Trans R Soc Lond B Biol Sci 2016; 371

Cummings JL, Vinters H V, Cole GM, Khachaturian ZS. Alzheimer's disease Etiologies, pathophysiology, cognitive reserve, and treatment opportunities. Neurology 1998; 51: S2–17.

von Ellenrieder N, Pellegrino G, Hedrich T, Gotman J, Lina JM, Grova C, et al. Detection and Magnetic Source Imaging of Fast Oscillations (40–160 Hz) Recorded with Magnetoencephalography in Focal Epilepsy Patients. Brain Topogr 2016; 29: 218–31.

Englot DJ, Hinkley LB, Kort NS, Imber BS, Mizuiri D, Honma SM, et al. Global and regional functional connectivity maps of neural oscillations in focal epilepsy. Brain 2015; 138: 2249–62.

Englot DJ, Konrad PE, Morgan VL. Regional and global connectivity disturbances in focal epilepsy, related neurocognitive sequelae, and potential mechanistic underpinnings. Epilepsia 2016; 57: 1546–57.

Ferguson MA, Lim C, Cooke D, Darby RR, Wu O, Rost NS, et al. A human memory circuit derived from brain lesions causing amnesia. Nat Commun 2019; 10: 3497.

Fernandes JM, da Silva AM, Huiskamp G, Velis DN, Manshanden I, de Munck JC, et al. What does an epileptiform spike look like in MEG? Comparison between coincident EEG and MEG spikes. J Clin Neurophysiol 2005; 22: 68–73.

Fischl B. FreeSurfer. Neuroimage 2012; 62: 774–81.

Garcés P, López-Sanz D, Maestú F, Pereda E. Choice of magnetometers and gradiometers after signal space separation. Sensors (Switzerland) 2017; 17: 2926.

Garces P, Martin-Buro MC, Maestu F. Quantifying the test-retest reliability of MEG resting state functional connectivity. Brain Connect 2016; 6: brain.2015.0416.

Garcia-Marin V, Blazquez-Llorca L, Rodriguez J-R, Boluda S, Muntane G, Ferrer I, et al. Diminished perisomatic {GABAergic} terminals on cortical neurons adjacent to amyloid plaques. Front Neuroanat 2009; 3: 28.

Hauser WA, Morris ML, Heston LL, Anderson VE. Seizures and Myoclonus in Patients with Alzheimer's Disease. Neurology 1986; 36: 1226–30.

Hincapié A-SS, Kujala J, Mattout J, Pascarella A, Daligault S, Delpuech C, et al. The impact of MEG source reconstruction method on source-space connectivity estimation: A comparison between minimum-norm solution and beamforming. Neuroimage 2017; 156: 29–42.

Jack CR, Wiste HJ, Weigand SD, Therneau TM, Lowe VJ, Knopman DS, et al. Defining imaging biomarker

For Review Only

cut points for brain aging and Alzheimer's disease. Alzheimers Dement 2017; 13: 205–16.
Lam AD, Sarkis RA, Pellerin KR, Jing J, Dworetzky BA, Hoch DB, et al. Association of epileptiform abnormalities and seizures in Alzheimer disease. Neurology 2020; 95: e2259–70.
Lega B, Dionisio S, Bingaman W, Najm I, Gonzalez-Martinez J. The gamma band effect for episodic memory encoding is absent in epileptogenic hippocampi. Clin Neurophysiol 2015; 126: 866–72.
Lin J-JJ, Umbach G, Rugg MD, Lega B. Gamma oscillations during episodic memory processing provide evidence for functional specialization in the longitudinal axis of the human hippocampus. Hippocampus 2019; 29: 68–72.
López-Sanz D, Bruña R, Garcés P, Martín-Buro MC, Walter S, Delgado ML, et al. Functional Connectivity Disruption in Subjective Cognitive Decline and Mild Cognitive Impairment: A Common Pattern of Alterations. Front Aging Neurosci 2017; 9: 109.
López ME, Turrero A, Cuesta P, Rodríguez-Rojo IC, Barabash A, Marcos A, et al. A multivariate model of time to conversion from mild cognitive impairment to Alzheimer's disease. Geroscience 2020; 42: 1715–32.
López MEME, Turrero A, Cuesta P, López-Sanz D, Bruña R, Marcos A, et al. Searching for Primary Predictors of Conversion from Mild Cognitive Impairment to Alzheimer's Disease: A Multivariate Follow-Up Study. J Alzheimer's Dis 2016; 52: 133–43.
Maestú F, Peña J-MJ-M, Garcés P, González S, Bajo R, Bagic A, et al. A multicenter study of the early detection of synaptic dysfunction in Mild Cognitive Impairment using Magnetoencephalography-derived functional connectivity. NeuroImage Clin 2015; 9: 103–9.
Maheshwari A, Marks RL, Yu KM, Noebels JL. Shift in interictal relative gamma power as a novel biomarker for drug response in two mouse models of absence epilepsy. Epilepsia 2016; 57: 79–88.
Maris E, Oostenveld R. Nonparametric statistical testing of EEG- and MEG-data. J Neurosci Methods 2007; 164: 177–90.
Matsumoto JY, Stead M, Kucewicz MT, Matsumoto AJ, Peters PA, Brinkmann BH, et al. Network oscillations modulate interictal epileptiform spike rate during human memory. Brain 2013; 136: 2444–56.
McKhann G, Knopman DS, Chertkow H, Hymann B, Jack CR, Kawas C, et al. The diagnosis of dementia due to Alzheimer's disease: Recommendations from the National Institute on Aging- Alzheimer's Association workgroups on diagnostic guidelines for Alzheimer's disease. Alzheimers Dement 2011; 7: 263–9.
Mormann F, Lehnertz K, David P, E. Elger C. Mean phase coherence as a measure for phase synchronization and its application to the EEG of epilepsy patients. Phys D Nonlinear Phenom 2000; 144: 358–69.
Nakamura A, Cuesta P, Fernández A, Arahata Y, Iwata K, Kuratsubo I, et al. Electromagnetic signatures of the preclinical and prodromal stages of Alzheimer's disease. Brain 2018; 141: 1470–85.
Nakamura A, Cuesta P, Kato T, Arahata Y, Iwata K, Yamagishi M, et al. Early functional network alterations in asymptomatic elders at risk for Alzheimer's disease [Internet]. Sci Rep 2017; 7Available from: https://doi.org/10.1038%2Fs41598-017-06876-8
Nolte G. The magnetic lead field theorem in the quasi-static approximation and its use for magnetoencephalography forward calculation in realistic volume conductors. Phys Med Biol 2003; 48: 3637–52.
Nowak R, Santiuste M, Russi A. Toward a definition of MEG spike: parametric description of spikes recorded simultaneously by MEG and depth electrodes. Seizure 2009; 18: 652–5.
Oostenveld R, Fries P, Maris E, Schoffelen J-M. FieldTrip: Open source software for advanced analysis of MEG, EEG, and invasive electrophysiological data. Comput Intell Neurosci 2011; 2011: 156869.
Palop JJ, Mucke L. Network abnormalities and interneuron dysfunction in Alzheimer disease. Nat Rev Neurosci 2016; 17: 777–92.
Pusil S, López ME, Cuesta P, Bruña R, Pereda E, Maestú F. Hypersynchronization in mild cognitive impairment: the 'X' model. Brain 2019; 142: 3936–50.
Ramírez-Toraño F, Bruña R, de Frutos-Lucas J, Rodríguez-Rojo IC, Marcos de Pedro S, Delgado-Losada ML, et al. Functional Connectivity Hypersynchronization in Relatives of Alzheimer's Disease Patients: An

For Review Only

Early E/I Balance Dysfunction? Cereb Cortex 2020; 31: 1–10.
Rampp S, Rössler K, Hamer H, Illek M, Buchfelder M, Doerfler A, et al. Dysmorphic neurons as cellular source for phase-amplitude coupling in Focal Cortical Dysplasia Type II. Clin Neurophysiol 2021; 132: 782–92.
Ren L, Kucewicz MT, Cimbalnik J, Matsumoto JY, Brinkmann BH, Hu W, et al. Gamma oscillations precede interictal epileptiform spikes in the seizure onset zone. Neurology 2015; 84: 602–8.
Rouhinen S, Siebenhühner F, Palva JM, Palva S, Matias Palva J, Palva S. Spectral and Anatomical Patterns of Large-Scale Synchronization Predict Human Attentional Capacity. Cereb Cortex 2020; 30: 5293–308.
Scarmeas N, Honig LS, Choi H, Cantero J, Brandt J, Blacker D, et al. Seizures in Alzheimer Disease: Who, When, and How Common? Arch Neurol 2009; 66: 992–97.
Sederberg PB, Schulze-Bonhage A, Madsen JR, Bromfield EB, McCarthy DC, Brandt A, et al. Hippocampal and neocortical gamma oscillations predict memory formation in humans. Cereb Cortex 2007; 17: 1190–6.
Sestieri C, Corbetta M, Romani GL, Shulman GL. Episodic memory retrieval, parietal cortex, and the default mode network: functional and topographic analyses. J Neurosci 2011; 31: 4407–20.
Tan RJ, Rugg MD, Lega BC. Direct brain recordings identify hippocampal and cortical networks that distinguish successful versus failed episodic memory retrieval. Neuropsychologia 2020; 147: 107595.
Taulu S, Simola J. Spatiotemporal signal space separation method for rejecting nearby interference in MEG measurements. Phys Med Biol 2006; 51: 1759–68.
Tzourio-Mazoyer N, Landeau B, Papathanassiou D, Crivello F, Etard O, Delcroix N, et al. Automated anatomical labeling of activations in SPM using a macroscopic anatomical parcellation of the MNI MRI single-subject brain. Neuroimage 2002; 15: 273–89.
Ung H, Cazares C, Nanivadekar A, Kini L, Wagenaar J, Becker D, et al. Interictal epileptiform activity outside the seizure onset zone impacts cognition. Brain 2017; 140: 2157–68.
Van Veen BD, van Drongelen W, Yuchtman M, Suzuki A, Veen BD Van, Drongelen W Van, et al. Localization of brain electrical activity via linearly constrained minimum variance spatial filtering. IEEE Trans Biomed Eng 1997; 44: 867–80.
Vossel KA, Beagle AJ, Rabinovici GD, Shu H, Lee SE, Naasan G, et al. Seizures and epileptiform activity in the early stages of Alzheimer disease. JAMA Neurol 2013; 70: 1158–66.
Vossel KA, Ranasinghe KG, Beagle AJ, Mizuiri D, Honma SM, Dowling AF, et al. Incidence and Impact of Subclinical Epileptiform Activity in Alzheimer's Disease. Ann Neurol 2016: 1–54.
Vossel KA, Tartaglia MC, Nygaard HB, Zeman AZ, Miller BL. Epileptic activity in Alzheimer's disease: causes and clinical relevance. Lancet Neurol 2017; 16: 311–22.
Yeshurun Y, Nguyen M, Hasson U. The default mode network: where the idiosyncratic self meets the shared social world. Nat Rev Neurosci 2021; 22: 181–92.
Zalesky A, Fornito A, Bullmore ET. Network-based statistic: Identifying differences in brain networks. Neuroimage 2010; 53: 1197–207.

For Review Only
