# Supplementary material for: Gamma band functional connectivity reduction in patients with amnestic mild cognitive impairment and epileptiform activity
Source: Brain Commun. 2022 Feb 3;4(2):fcac012. doi: 10.1093/braincomms/fcac012 (PMC8914494; doi:10.1093/braincomms/fcac012)
Supplement: fcac012_Supplementary_Data [file fcac012_supplementary_data.zip › Supplementary Materials.docx]

**Supplementary Materials**

| **Patient** | **Location (number of spikes)** |
| --- | --- |
| 1 | Left midtemporal superior gyrus (2) |
| 2 | Right mesial temporal, basal temporal, and posterior temporal (4) |
| 3 | Left=Right mid superior temporal sulcus (2) |
| 4 | Right mesial temporal (1) |
| 5 | Left frontal opercular (1) |
| 6 | Right basal temporal (1) |
| 7 | Right anterior temporal (2) |
| 8 | Left posterior temporal (1) |
| 9 | Right anterior temporal (1), Right frontal opercular (1) |
| 10 | Right peri-Sylvian, inferior frontal gyrus (2) |
| 11 | Left anterior and mid temporal temporal (4) |
| 12 | Right insular (intra-sylvian) (1) |
| 13 | Left mesial temporal (1) |
| 14 | Right inferior frontal gyrus (1) |
| 15 | Left frontal opercular (1) |
| 16 | Right frontal opercular (1) |
| 17 | Right frontal paracentral dorsolateral (1) |
| 18 | Bilateral frontal opercular L>>R (9) |
| 19 | Right mid temporal (1) |
| 20 | Right basal temporal, Right parietal, right insular (3) |

**Supplementary Table 1.** Description of the epileptiform activity found for each MCI (EA+) patient. **L**, left. **R**, right, number of discharges in brackets; laterality is indicated by = (symmetric) and >> for dominance of one hemisphere. The temporal lobe is presented in several sub-regions: mesial, basal, anterior temporal, mid temporal, posterior temporal. Frontal lobe sources are presented in sub-regions: inferior frontal, fronto-opercular, mesial frontal, dorsolateral,

| **Patient** | Primary cluster | Secondary-1 | Secondary-2 |
| --- | --- | --- | --- |
| 1 | 0.1705 | 0.1992 | 0.1492 |
| 2 | 0.1648 | 0.1736 | 0.1600 |
| 3 | 0.1613 | 0.1737 | 0.1550 |
| 4 | 0.1617 | 0.1817 | 0.1374 |
| 5 | 0.1814 | 0.1914 | 0.1683 |
| 6 | 0.1725 | 0.1891 | 0.1429 |
| 7 | 0.1713 | 0.1977 | 0.1402 |
| 8 | 0.1694 | 0.1914 | 0.1409 |
| 9 | 0.1692 | 0.1797 | 0.1472 |
| 10 | 0.1687 | 0.1809 | 0.1460 |
| 11 | 0.1685 | 0.1853 | 0.1640 |
| 12 | 0.1549 | 0.1644 | 0.1334 |
| 13 | 0.1763 | 0.1910 | 0.1565 |
| 14 | 0.1618 | 0.1828 | 0.1418 |
| 15 | 0.1742 | 0.1975 | 0.1365 |
| 16 | 0.1718 | 0.1841 | 0.1370 |
| 17 | 0.1685 | 0.1769 | 0.1584 |
| 18 | 0.1649 | 0.1917 | 0.1358 |
| 19 | 0.1687 | 0.1765 | 0.1554 |
| 20 | 0.1673 | 0.1856 | 0.1517 |

**Supplementary Table 2.** MCI-EA+ individual normalized functional connectivity of each significant marker of the manuscript. Markers are displayed in Figure 3 of the manuscript.
