# Supplementary material for: Gamma band functional connectivity reduction in patients with amnestic mild cognitive impairment and epileptiform activity
Source: Brain Commun. 2022 Feb 3;4(2):fcac012. doi: 10.1093/braincomms/fcac012 (PMC8914494; doi:10.1093/braincomms/fcac012)
Supplement: fcac012_Supplementary_Data [file Original Submission - Author's Response.pdf › Original Submission - Author's Response.pdf]

August 4, 2021

Dr. Alberto Lleo
Editor,
Brain Communications

Manuscript #BRAINCOM-2021-090

Dear Dr. Alberto Lleo,

We attach our revised manuscript entitled "***Gamma band functional connectivity reduction in patients with amnestic mild cognitive impairment and epileptogenic activity***" to be considered for publication in Brain Communications.

We would like to thank the editor and reviewers for their thoughtful and positive comments. We have carefully revised our manuscript and have responded to the individual points raised by the editor and reviewers. We hope the manuscript is now suitable for publication.

The re-submission includes the revised manuscript with revisions highlighted in red. In addition, we have created two new figures inspired by the reviewers' comments.

We look forward to working with you to address any potential remaining concerns in this revision.

Sincerely,

On behalf of the co-authors,
Pablo Cuesta
Laboratory of Cognitive and Computational
Neuroscience Lab. UCM, Madrid, Spain.

Reviewer 1.

Comments to the Author
The authors study differences in network connectivity in patients with MCI with and without activity reminiscent of interictal epileptic discharges (EA). They report gamma-band disruption in patients with EA in right temporal-frontal/medial networks, which were found to be related to gray matter atrophy. The study provides evidence on a potential link between MCI/Alzheimer's disease and epileptic activity, a highly relevant topic both scientifically and clinically. The applied methodology is state of the art and the results largely support the conclusions. I still have a number of questions, comments and suggestions.

I'm not sure if the term „epileptogenic" activity is adequate in the presented context. In contrast to the more neutral (in my opinion at least) "epileptic" or "epileptiform" activity, "epileptogenic" implies a link to generation of epilepsy. While this could be true, I'd believe that this connotation might be too hypothetical at the moment. Then again, this might overinterpret the term.

We thank the reviewer for the positive comments.

Following both reviewers' suggestions, we now describe this as *epileptiform activity* throughout the manuscript.

Not necessarily contradicting the authors' statement on decreased gamma activity with the appearance of epileptic discharges in an animal model of AD, gamma increases in the vicinity of epileptic foci have also been demonstrated (e.g. (Ren et al., 2015; von Ellenrieder et al., 2016; Rampp et al., 2021)), which seem to be associated with the epilepsy, complementing the mentioned findings on gamma changes during cognitive tasks.

The reviewer raises an important point regarding the relation of gamma and EA. The three articles referenced by the reviewer assess gamma band activity in the regions of the seizure onset zone. In our case, the gamma band suppression was not associated with a particular region associated with the dipole-spike localization (see new Figure 1 in the new version of the manuscript). Our patient cohort came from a previous project focused on the study of the prodromal stages of Alzheimer's Disease. When we assessed the existence of epileptiform activity on these patients, we inspected about 18 minutes of brain activity (this new information is clarified in the new version of the manuscript). With this amount of data, we were able to find EA in 31% of MCI patients. Notwithstanding, the average number of spikes we found was 2.04 ± 1.79 per MCI EA+ patient, indicating that the incidence of EA in our sample was low. Of course, more data for extended periods of time would have led to a higher incidence of EA, but in our data the epileptiform activity was scarce and not clearly associated with an epileptogenic region or a particular epileptic profile (our participants did not have seizures). Last but also important, the studies (Ren *et al.*, 2015; Rampp *et al.*, 2021) were carried out with invasive EEG techniques. The translation of the local effects obtained with intracranial EEG to macroscopic methodologies such as MEG is not obvious. Regarding the gamma oscillations, in our study we used the classical lower gamma band (30-45 Hz) whereas in the three papers previously referred to, the gamma band was defined much broader (30 to

100 Hz). Given the above couple of points, we believe our study is very different from the ones cited by the reviewer. Furthermore, the three papers reported data from epileptic patients, and this could reflect a different severity of the epileptiform phenomena and the associated epileptic networks. Actually, the gamma band increase was associated with seizure onset as well as with dysmorphic neurons (Rampp *et al.*, 2021), which could be a different type of neuropathological alteration than the one typically found in AD.

The reviewer raises the important topic of relating the previous studies with our results here and addressing whether the functional connectivity reduction in the gamma range is due to epileptiform activity or the memory network impairment. We have modified the manuscript adding a paragraph in the Discussion section to cite these three articles and address these issues:

"It is important to discuss whether the reduction of functional connectivity in the group of MCI EA+ patients was caused by the epileptiform activity epileptiform activity itself or to the malfunction of the memory networks. In epilepsy, electrophysiological data tend to show an increased high gamma frequency (30-100Hz) associated with epileptiform activity (Ren et al., 2015; von Ellenrieder et al., 2016; Rampp et al., 2021). This seems to be counterintuitive with our findings assessing MCI patients in the current study. However, there are some critical differences between those studies and ours. Whilst the cited papers assessed epileptic patients, the presence of epileptiform activity in our MCI EA+ patients were scarce (patients did not have seizures), indicating that EA itself does not sustain the network malfunction. This fact is important because the neuropathological findings in epilepsy may differ from those typically found along the AD continuum. Our data consisted of non-invasive MEG recordings, whilst (Ren et al., 2015; Rampp et al., 2021) were carried out using intracranial EEG. The results from these two methodologies are difficult to interpret together since the local effects found with intracranial EEG are quite different to the macroscopic signals assessed with non-invasive EEG/MEG. Besides the differences in the data and technique, there are some methodological differences such as the different gamma band definitions. We focused on the classical gamma band (30-45 Hz) rather than the broader definitions used in the referenced studies. Given the differences in technique and methodology, our study offers new and different information in the underlying processes that occur in tandem with the development of AD neuropathology. Therefore, our findings of aberrant functional connectivity in MCI patients are more consistent with a disruption of the episodic memory networks. This disruption cannot be explained by the epileptogenic activity alone. In fact, we hypothesize that abnormal network functioning is a risk factor for EA, and hence that MCI EA+ patients might be at higher risk of conversion to dementia. EA potentially contributes to network decline."

Additionally, we have added a new paragraph at the beginning of the results with more information about the epileptiform activity found in our patients.

Of the 63 patients in the cohort, MEG was positive for EA in 20 patients (31.75%). The average number of spikes in the patients that showed epileptiform activity was 2.04 ± 1.79. Most of the spikes detected in MCI patients were in broad regions encompassing temporal (58%) and frontal (24%) areas. None of the subjects showed a clear cluster of EA, nor had seizures. Examples of EA discharges for two patients are shown in Figure 2.

Finally, we have added a new paragraph at the end of the discussion to acknowledge limitations due to the low incidence of EA.

"Another important limitation is the low incidence of EA in our MCI EA+ patients and its heterogeneous localization pattern. The low incidence could be partially underestimated due to the limited amount of data. Longer and more epilepsy-specific recordings could have allowed both the detection of more MCI EA+ patients, and a better characterization of the EA in the MCI EA+ patients. Finally, to identify and exclude MEG-unique normal variants, EEG is needed. Our preliminary data were recorded without simultaneous EEG. Hence there is a possibility that source heterogeneity is inflated."

Subjects: I assume the subjects were recruited prospectively and represent a consecutive series?

Participants were originally recruited (prospectively) from the authors for a different study that aimed to detect MEG signatures that differentiate MCI from age-matched controls (Maestú *et al.*, 2015). Here, we used data from a subset of patients from our original study (the MCI patient cohort) to evaluate the presence of EA and its impact on functional connectivity. We now cite this study to make it clear we are using patients from the same cohort.

"Sixty-three amnestic MCI patients were recruited from the Hospital Universitario San Carlos (Madrid, Spain) (Maestú *et al.*, 2015)."

What is the reason for the T1/T2 MRI within 54 weeks before the MEG inclusion criterion? Fr MEG analysis, new T1 were acquired, as I understand?

In the original project, the exclusion criteria for year 1 were fixed to facilitate the recruitment from different hospitals of the Madrid area. The average time between the MEG recording and the MRI scan was 3 months. However, in some subjects (12%) we had to use previous MRIs because of motion artifacts (patient inability to stay still inside the MRI) or because they were afraid of having another MRI and could not perform this test again. This is the reason why the time range between MRI and MEG appears long. In the new version of the manuscript, we have included a new sentence to be more precise:

"(on average, the time period between the MEG and MRI recordings was 3 months)"

Recording time: 5 minutes of recording, while potentially adequate of investigating connectivity changes in relation to MCI, would be rather short to capture interictal epileptic discharges at least in a patient with focal epilepsy. Potentially, this would lead to an underestimation of EA occurrence in the study. However, this would likely decrease the contrast between groups, suggesting that if this is a problem, the results would potentially also underestimate the observed effects.

We thank the reviewer for highlighting this issue which allowed us to improve the description in the methods. We used 5 minutes of resting-state data with eyes closed for functional connectivity analysis. However, our complete MEG protocol consisted of three

recordings: 5-minute resting state eyes-closed, 5-minutes resting state eyes-open, and 10-minutes of passive face viewing while sitting comfortably inside a magnetically shielded room. For EA screening, we visually inspected all three recordings comprising in total approximately 20 minutes. We have added this text to the manuscript:

"A scheme of the methodological pipeline can be seen in Figure 1. MEG signals were acquired using a whole-head Elekta-Neuromag MEG system with 306 channels (Elekta AB, Stockholm, Sweden) at the Center for Biomedical Technology (Madrid, Spain). Data was collected at a sampling frequency of 1000 Hz and online band-pass filtered between 0.1 and 330 Hz. The MEG protocol consisted of 5-minute resting state eyes-closed, 5-minutes resting state eyes-open and 10-minutes of passive face viewing while sitting comfortably inside a magnetically shielded room. For functional connectivity analysis, we used the 5 minutes of resting state eyes closed recordings. For epileptiform activity screening, we visually inspected all three recordings comprising approximately 20 minutes".

Minor issue: numbers in table 1 should use dots instead of commas

Corrected.

Did you investigate the localizations of the EA? Were they consistent and/or did they localize to the MCI EA+ cluster? If so, one explanation for the lower connectivity in the gamma band could be due to the local gamma being generated or associated with the EA patterns themselves, which would show local increases with limited synchronization with distant areas. Furthermore, filter ringing due to two-pass filtering of sharp EA patterns with a high order filter (although FIR filters were used) could induce "false" gamma, which would probably also show only local increases.

This is an insightful hypothesis about the relationship between EA and gamma band reduction. We have now added the information that the average number of spikes in the MCI EA+ patients was 2.04 ± 1.79. This means that the presence of epileptiform activity was scarce. Also, none of our patients had seizures. In addition, most of the spikes detected in MCI patients were in diverse regions encompassing temporal (58%) and frontal (24%) areas. None of the subjects showed a clear cluster of EA, as it typically happens in focal epilepsy. Therefore, neither the spike density nor the spike location seemed to be the cause of the gamma hyposynchrony. In fact, the localization of the spikes in the frontal lobe were in dorsal and ventral regions while only ventral areas were involved in the gamma band clustering. Similarly, the temporal lobe was hypo-connected with the inferior parietal region, a lobule where a rather low number of spikes were located (2%). For these reasons, we believe that the hyposynchrony of the gamma band is instead consistent with a disruption of the memory network, with EA potentially a risk factor that accelerates such disruption. Importantly, the FC effect consisted of an alteration of the global gamma FC of the primary cluster, implying a widespread effect (that can be seen in both secondary clusters) rather than a local effect.
Regarding the potential issue of a filter ringing effects caused by sharp EA patterns, the incidence of EA was so low that any ringing effects would have negligible impact on signal power.

We have added this text in the Discussion section:

"We cannot rule out the possibility of a locally produced increase in gamma band frequency oscillations, generated close to the EA. In the context of epilepsy, EA may induce more random phases that cause a reduction in long-distance synchronization. The gamma band hyposynchronized network involves inferior frontal, temporal, and inferior parietal regions. While the spikes found in this population of MCI participants were located essentially in frontal and temporal lobes, they were distributed in broader areas encompassing dorsal aspects of the temporal and frontal regions. Furthermore, the incidence of EA was low, suggesting that this activity did not drive the macroscopic gamma band FC depletion. It is possible, then that this gamma band network disruption reflects the episodic memory dysfunction rather than the EA itself. Nonetheless, it appears that EA is associated with the disruption of this network"

Were correlation analyses corrected for multiple comparisons?

The post hoc correlation analyses were not corrected for multiple comparisons. This is because this analysis aimed to identify the overall association of FC disruption with anatomy rather than focus on specific areas. As it can be seen in Table 3, all correlations had the same sign, positive. This consistent finding indicates that reduced FC between these two clusters was associated with higher gray matter atrophy across several brain regions in the MCI EA+ patients. Notwithstanding, we have applied a FDR ($q = 0.05$) test for multiple comparison correction and all p values displayed in Table 3 remained significant ($p < 0.05$). We have added the following text to the table 3 caption:

"* *p values remained significant after FDR (q = 0.05) correction".*

Minor issue: p. 12 line 12: "these" instead of "this" and following sentence "have been associated"

Corrected.

The lack of group differences or correlations to behavioral variables/cognitive performance is somewhat puzzling. Would you believe that this is due to intact compensation mechanisms? Could more detailed neuropsychological evaluation yield differences? Or is this due to the (shortly mentioned) fact that only resting-state activity was evaluated? This aspect should be discussed.

The reviewer raises an interesting point. The lack of differences was initially quite intriguing for us as well. Here, as commented before, one important point is that the sample was focused (originally) in Alzheimer's Disease and not in assessing neurophysiological alterations associated with an epileptic phenotype. On one hand, this factor constrained methodological aspects such as the neuropsychological assessment, defined for MCI in the context of the AD continuum, or the availability of only resting state eyes closed activity for the FC analyses. On the other hand, it is true that the latter

is broadly assessed in interictal epileptic literature, and that the neuropsychological assessment is designed to capture the memory failure that typically emerges with the AD progression. Nevertheless, our point here was to assess the epileptiform activity within the AD context. Thus, we cannot be more precise, with the evidence on our hands, about the extent of the influence that the emergence of epileptic activity exerts on the brain integrity or cognitive performance. However, the fact that only the EA+ group showed significant correlations with brain structural integrity might indicate that the appearance of epileptiform activity could be reflecting alterations at the structural level. These structural alterations were not found in the EA- group.

Regarding the use of resting state, it is true that it could influence our results. But this issue is present in most interictal literature. Resting state has been proven to be useful to assess brain functional integrity alterations, before any structural or cognitive damage could be detected (Nakamura *et al.*, 2017, 2018a). Therefore, we believe that our approach helps in the understanding of the electrophysiological processes that occur unnoticed when an underlying neuropathological process develops. Of note, the gamma FC patterns we found are different from the typical electrophysiological "slowing" effect often found in AD. This fact suggests that the gamma reduction may be an epiphenomenon of network disruption associated with new neuropathological pathways that could be accelerating the neuronal damage induced by the dementia progression. We aim to address this specific factor in a new study we just began with an increased number of patients.

We have included the following paragraph in the Discussion section:

"There were no differences between groups in cognitive performance, nor in brain structural integrity. The only difference between groups was found for the FC assessment. However, the fact that only the EA+ group showed significant correlations with brain structural integrity might indicate that the appearance of EA could reflect alterations at the structural level. This relationship between function and structure was not found in the EA- group. The precedence in bran resting state of functional abnormalities to any structural or cognitive damage has been stated in previous studies (Nakamura *et al.*, 2017, 2018b). *In this study, the reported gamma FC pattern is different from the typical electrophysiological "slowing" effect usually found in AD. This fact suggests that the gamma depletion could be an epiphenomenon of network disruption associated with new neuropathological pathways that could accelerate the neuronal damage induced by the dementia progression"*

The results show interesting parallels to the finding of transient cognitive impairment (TCI) in patients with epilepsy (e.g. (Ung et al., 2017)), which suggest that epileptic activity itself may interfere with cognitive processes. Since the spatial distribution of epileptic activity seems to play a role in this phenomenon, it would be interesting to also describe and discuss the EA localization in relation to the observed findings.

As we discussed in the points above, the type of epileptiform activity we found in our patients was broadly distributed and scarce. From a clinical point of view, they were not considered patients with epilepsy. Thus, it is difficult to explore direct associations between EA localization and cognitive impairment with the current data. Additionally, there were no differences in neuropsychological performance between the EA+ and EA-

MCI groups. We could hypothesize, based on the neuropathological evolution of the AD continuum (Jack and Holtzman, 2013), that the presence of EA could indicate network malfunction that is associated with loss of brain structural integrity and ends up triggering cognitive impairment. However, the question raised by the reviewer is important and it will need to be addressed in future projects, specifically designed to study epileptiform activity along with neurodegeneration. We have included the following paragraph to the discussion section:

"Past work has shown that EA may induce transient cognitive impairment, indicating how this phenomenon interferes with cognitive processing (Ung et al., 2017). Since episodic memory decline is one of the initial symptoms of AD, the localization of EA at the medial temporal lobe regions could be associated with cognitive impairment. However, the scarce incidence, likely influenced for having only 20 minutes long recordings, and heterogeneity of localization of EA in our cohort of MCI patients make it difficult to statistically assess this hypothesis".

On the bottom line, would you think that EA is actively contributing to cognitive decline? Or is this "only" an epiphenomenon of network disruption, but a potentially valuable indicator nonetheless?

In our view, EA is a sign of loss of the E/I balance. The loss of inhibitory contacts leads to cortical hyperexcitability and spikes. This EA is not focal because this loss of inhibitory terminals occurs in a widespread manner. Therefore, EA could be seen as a telltale sign of a neurodegenerative process affecting, initially, inhibitory terminals due to the toxicity exerted by the amyloid plaques. In this sense, it is a parallel neurophysiological phenomenon to cognitive decline, affecting the organization of functional networks and incidentally associated with cognitive decline. We agree with the reviewer that this neurophysiological sign has a potential value for establishing the stage of the disease process as well as future clinical status.

Reviewer 2.

The term of "epileptogenic activity" means that there is certainly an epileptic activity. If I understand correctly, the patients did not have a diagnosis of epilepsy but some EEG alterations resembling epilepsy were detected. In this case, it might be more accurate to use the term "epileptiform activity"

We fully agree with the reviewer that the term epileptiform activity is more accurate. In fact, this was also argued by reviewer 1. We have revised the manuscript accordingly.

Page 4, line19. In case the two independent experienced radiologists did not agree, how did the authors decide? Did they request a third opinion to another experienced radiologist?7

We apologized for not being clear in the original submission. Two neurophysiologists reviewed the MEG traces to detect EA. Both reviewers inspected the data together and decisions on the presence of EA discharges were taken by consensus. In case of disagreement, resolution relied on the opinion of the most experienced neurophysiologist (author MEF). We have added the following text in the Method section:

"A MEG expert (MEF) and a neurologist trained in MEG reading (MOU) screened MEG signals for EA (see Figure 2). Both reviewers inspected the data together and decisions on the presence of EA discharges were taken by consensus. In case of disagreement, resolution relied on the opinion of the most experienced neurophysiologist (MEF). No additional opinion from a third reader was obtained. Localization of the spikes were obtained using single dipole modeling, which is the accepted approach in clinical MEG (see CPG 1, (Bagić et al., 2011). Raw data was reviewed after Maxfilter processing. We defined EA as a transient signal that was clearly distinguished from background activity, and with a pointed peak component (Fernandes *et al.*, 2005; Nowak, Santiuste and Russi, 2009). *In order to be valid, only statistically significant sources (reduced Chi square ≥ 1 and ≤ 2, confidence volume <1000mm3, source strength between 100-500 nanoamperes, goodness of fit >75%) were accepted. Source localization of the dipoles was performed in the DANA Elekta Neuromag Software (Elekta AB, Stockholm, Sweden) superimposing the dipoles on the patient's structural MRI. Depending on the presence or absence of EA, patients were divided into two groups: MCI EA+ and MCI EA-, comprising patients with and without EA, respectively."*

Page 5, line 8. In the artifact-free data, further analyzed, did the authors find any signs of the epileptiform activity? Did an expert in epilepsy check the epochs in order to identify possible spikes? The presence of spikes should affect the analysis, especially in terms of frequency bands. In this case, I think the authors should discuss their findings with respect to their presence.

The trials used for FC were processed to clean any artefacts using visual inspection, data filtering and ICA, as indicated in the method section. Furthermore, the patients did not show seizures or any consistent epileptiform activity; MCI EA+ patients had on average

2.04 spikes (new information added to the revised manuscript). Thus, we consider that the possible influence in the FC computations would be negligible.

(4) Page 5, line 23. The approach used to identify the epileptiform activity is quite clear. However, I do not understand if the authors have selected the patients with an analysis of their electrical activity or with the localization of the dipoles. This aspect should be clarified and in case of the dipoles, the authors should explain this choice. Moreover, it might be interesting to specify how many spikes were found in each patient, in order to better describe the epileptiform activity, which is the crucial factor used to divided the investigated clinical cohort. Finally, if the MEG expert and neurologist trained in MEG reading did not agree, how did the authors decided? Did they request a third opinion to another experienced neurologist?

We have divided patients in two groups considering the presence or absence of EA. The localization of dipoles did not play a role in the selection of patients. However, localization in the frontal and temporal regions were more frequent. Also, as we responded in an earlier point, on average patients with epileptiform activity displayed 2.04 ± 1.79 spikes per recording, ranging from 1 to 9.

We have added the following paragraph in the Method section to clarify the selection of EA.

"A MEG expert (MEF) and a neurologist trained in MEG reading (MOU) screened MEG signals for EA (see Figure 2). Both reviewers inspected the data together and decisions on the presence of EA discharges were taken by consensus. In case of disagreement, resolution relied on the opinion of the most experienced neurophysiologist (MEF). No additional opinion from a third reader was obtained. Localization of the spikes were obtained using single dipole modeling, which is the accepted approach in clinical MEG (see CPG 1, (Bagić et al., 2011). Raw data was reviewed after Maxfilter processing. We defined EA as a transient signal that was clearly distinguished from background activity, and with a pointed peak component (Fernandes *et al.*, 2005; Nowak, Santiuste and Russi, 2009). *In order to be valid, only statistically significant sources (reduced Chi square ≥ 1 and ≤ 2, confidence volume <1000mm3, source strength between 100-500 nanoamperes, goodness of fit >75%) were accepted. Source localization of the dipoles was performed in the DANA Elekta Neuromag Software (Elekta AB, Stockholm, Sweden) superimposing the dipoles on the patient's structural MRI. Depending on the presence or absence of EA, patients were divided into two groups: MCI EA+ and MCI EA-, comprising patients with and without EA, respectively."*

(5) Page 5, lines 30-54. I think that in order to explain more clearly the procedure used for the connectivity analysis, it might be useful to present a figure with the sequences followed by the authors. For example, a brain image related to the 78 cortical areas of AAL with superimposed nodes. Then, a schematic representation of the whole-brain matrix of the 1202 nodes with the formula of PLV etc. to conclude with the brain map the authors obtained after the normalization of the node strengths, specifying that this approach was applied for each patient and frequency band.

Following the reviewer's suggestion, we have created a new figure that is included in the methods section (new Figure 1):

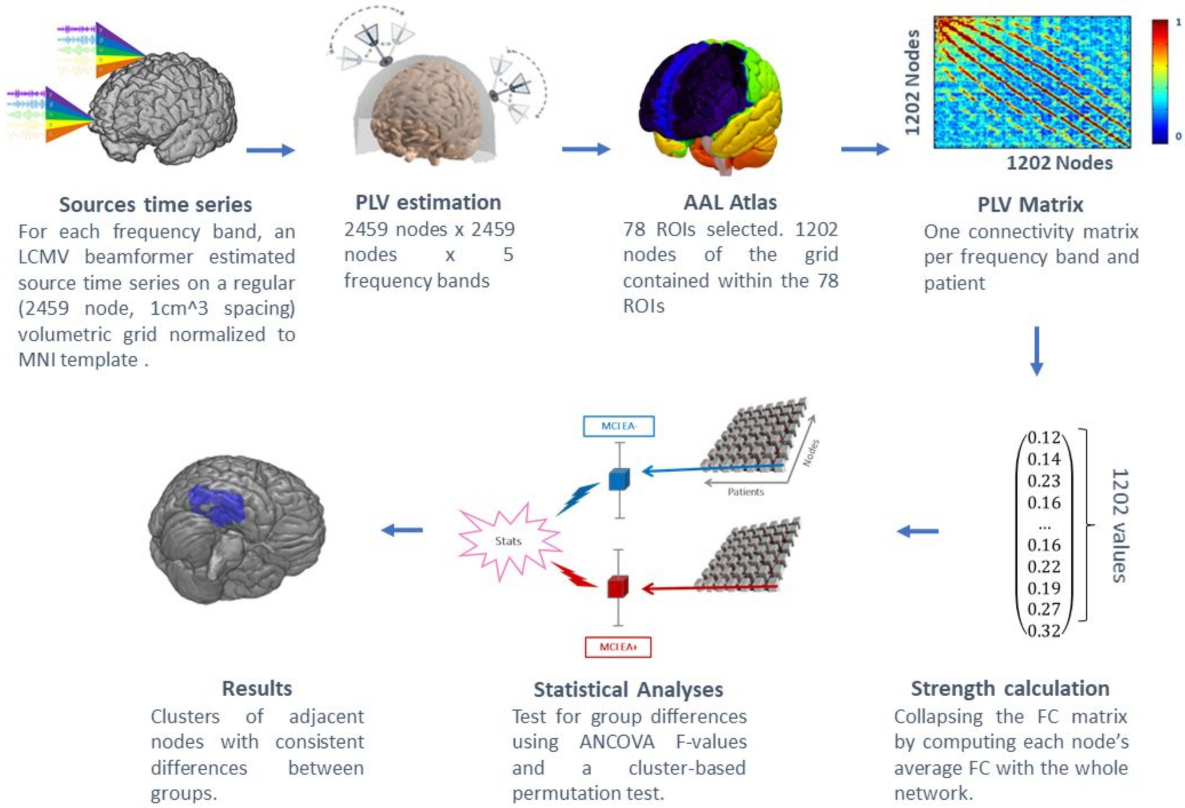

Figure 1. Schematic diagram of processing pipeline. For each frequency band, an LCMV beamformer estimated source time series in a regular volumetric grid of nodes. Phase locking values (PLV) were then estimated between every pair of nodes contained within one of 78 ROIs from the AAL atlas. These values were used to compute the normalized strength of each node, defined as the sum of its PLV with the rest of the nodes, divided by the number of connected nodes. Last, the strength values were subjected to statistical analyses using ANCOVA and a cluster-based permutation test to identify clusters of adjacent nodes with significant functional connectivity differences between the MCI EA+ and MCI EA- groups.

(6) Page 6, lines 15-18. In the manuscript, for each patient and for each frequency band, a brain map with normalized node strengths (1202 nodes) has been computed. After that, ANCOVA has been applied in order to identify the differences of strength's nodes between the experimental groups. This procedure gave another brain map composed by 1202 F-values regarding the comparisons of each node between the two clinical cohorts (line13). A threshold of p-value=0.005 was used to select the nodes which differs significantly among the two group of patients. This brain map, composed by suprathreshold F-values, was then additionally split in two: one with nodes that showed positive differences and another one with nodes with negative differences. At this point, the analysis was still at a node level. Then, the cluster level has been introduced. A cluster has to be composed by at least 12 nodes. How did the authors cluster the nodes? For example, can they identify a criterion such as the one discussed at line 32 and 33 for the seeds? Moreover, how

many clusters did the authors find in the positive and negative F-values maps? In the followed statistical procedure, did the authors include clusters from both positive and negative F-values map? It might be helpful to show in a figure the procedure with which the authors extracted the clusters, perhaps together with the previous one in a different panel (e.g. Fig.1b). In addition, I think that some part of the presentation should be rewritten in order to make the statistical analysis section more comprehensible.

(7) Page 6, line 33. Why did the authors think that comparing each node of the grid with the seed-cluster regions could provide information about a possible isolated, local contribution of few nodes to FC differences between groups? Which kind of statistical analysis did the authors apply to evaluate the existence of group differences in the FC in terms of seeds? Could the authors explain the sentence at line 37? Why did they decide to take in account only the clusters that did not overlap with the original seed-cluster?

(8) Page 6, line 43. This sentence should be clarified.

Answers to points 6, 7, and 8. We apologize for the confusion and thank the reviewer for giving us the opportunity to rewrite this section to improve clarity. Since all these questions are related, we address them together.

Following reviewer considerations, we have clarified the statistical analyses section, also providing a more formal description of the CBPT procedure (e.g., introducing the cluster-defining threshold, cluster mass statistic, etc):

"The assessment of significant group FC differences was based on a cluster-based permutation test (CBPT) described previously (Maris and Oostenveld, 2007; Zalesky, Fornito and Bullmore, 2010) *where the units of study were clusters of spatially adjacent nodes whose strength (weighted global connectivity) differed significantly between groups with the same sign. This procedure was applied independently for each frequency band, as implemented in Fieldtrip (Oostenveld et al., 2011). The methodology started by testing each of the 1202 nodes separately for strength differences between the two groups using an ANCOVA test while adjusting for the effects of age. This procedure yielded one F-statistic value per node and resulted in a volumetric map of 1202 F-statistic values. This F-statistic map was thresholded using a critical value corresponding to the 0.005 significance level for the F-statistic (cluster-defining threshold). Subsequently, the thresholded map was split into two maps corresponding to the voxels with connectivity MCI EA+ > MCI EA- or MCI EA+ < MCI EA-. For each map, a clustering procedure identified groups of adjacent nodes in the volume space, and the mass of each cluster was defined as the sum of the F-values of the nodes comprising each cluster (cluster mass statistic). We employed this measure because it reflects a combination of the topological extend (number of nodes) and the effect size (F-values). As an inclusion criterion to suppress spurious findings, we required the minimum size for each candidate cluster to be equal to 1% of the total nodes in the volume, and all cluster smaller than this size were automatically deemed non-significant.*

Then, to control for multiple comparisons, this entire procedure was repeated 5000 times after randomly shuffling the original group's labels and creating permutation samples of the original F-statistic maps. At each repetition, the maximum cluster mass statistic of the surrogate clusters was stored, thus constructing an empirical distribution of the maximum cluster mass statistic. This maximal null distribution enables us to compute the

p-value for each candidate cluster of the original data and thus the control of the family-wise error rate (FWER) at the cluster level. Only those clusters that survived the CBPT at $p < 0.05$ were considered for the subsequent analyses as potential MEG markers. As a representative value of these MEG markers, we computed the average strength of the nodes contained in the cluster. These values are shown in the boxplots of Figure 3 and were tested for differences between both groups with further ANCOVA test with age as covariate. These values were also used in a subsequent Spearman correlation analysis with cognitive and structural scores.

The significant clusters detected with the above procedure differed in global connectivity between the two groups. As a post-hoc analysis, we applied a seed-based procedure to determine whether the strength differences (i.e., the global FC of the cluster) were primarily driven by the existence of a few isolated connections rather than caused by a "global/widespread" effect (widespread connections of each original cluster to the rest of the brain). The seed region was calculated by taking all nodes that were in a radius of 20 mm from the physical mass center of the cluster. Then, we computed all 1202 FC values between each node and the cluster's mass center (seed), yielding a seed connectivity map. This map was subjected to the same clustering procedure described above. Only clusters that did not overlap with the original cluster were reported in this study to ensure reliable results that did not depend on the precise extent of the original cluster. Statistical analyses were carried out using Matlab R2020b (Mathworks Inc) and all tests were two-tailed.".

Since we initially adapted our methodology from the procedures described in (Maris and Oostenveld, 2007) and used in Fieldtrip (Oostenveld *et al.*, 2011), we will detail here the analysis pipeline step-by-step, concentrating in those steps where our methodology differs from the Maris procedure (i.e. basically to adapt the procedure to assess strength differences at the sources space). The CBPT detailed description can be found elsewhere (Maris and Oostenveld, 2007), but briefly this procedure (detailing our modifications) can be summarized as follows:

1. Every participant was represented with a vector of 1202 values corresponding to the strength computed as the average FC of each grid location and the whole network.
2. For each of these 1202 nodes, a statistical test between groups was applied. In the standard CBPT pipeline the authors used a t-statistic to test the null hypothesis of equality in the mean FC between two populations. However, in our study, we wanted to account for the possible influence of age in our results. Therefore, we used an Ancova test with this factor as a confounding variate, which resulted in obtaining F-statistics instead of t-statistics.
3. Then, the vector of F-statistics (as the reviewer pointed out) was subjected to a thresholding procedure. In our case we chose a critical F-value corresponding to a cluster configuration p-value of 0.005. We used a p-value of 0.005 to focus on any possible small subset of nodes with stronger differences between groups (rather than broad clusters with weak effects). In any case, the FWER is controlled at the level of clusters independently of the selected configuration p-value (Maris and Oostenveld, 2007; Zalesky, Fornito and Bullmore, 2010). The F-values of the original F-statistics vector smaller/larger than the critical value were set to 0/1,

respectively, ending up with one binary vector that could be treated as an assembly of nodes showing an effect of interest. In our pipeline we split this binary vector in two attending to the sign of the differences between groups, obtaining two binary vectors corresponding for the case MCI EA+ > MCI EA- and MCI EA+ < MCI EA-.

4. A clustering procedure was used to identify clusters in each binary vector. The clustering methodology is the one implemented in Fieldtrip and consists of detecting groups of adjacent nodes in the physical space. These clusters constituted the "candidate clusters of interest". To prevent spurious findings, we fixed a condition for the minimum "size" that a candidate cluster should accomplish. This condition corresponded with the 1% of the nodes involved in the study. Thus, the clustering procedure extracts all possible clusters (positives or negatives) that succeed the minimum size parameter.
5. For each candidate cluster, a cluster mass statistic was computed as the sum of the F-values associated with those cluster's strength differences. We employed this measure in the non-parametric permutation because it reflects a combination of the topological extent (number of nodes) and the effect size (F-values).
6. The next step required a permutation test procedure used to estimate an empirical null distribution. This test consisted of 5000 repetitions of the analysis pipeline after shuffling the participants labels, storing the maximum cluster mass.
7. The corrected p-value for a candidate cluster corresponded to the proportion of the permutation distribution with cluster-statistic values greater or equal than the cluster-statistic value of the original data. The use of the null distribution of maximal cluster mass ensures control of the FWER for the cluster as a whole, not being able to reject the null hypothesis at the level of single nodes (Maris and Oostenveld, 2007; Zalesky, Fornito and Bullmore, 2010).
8. As a representative value of the cluster, we computed the average strength of the nodes contained in the cluster. These values were represented in the boxplots of Figure 3 and were tested for differences between both groups with further ANCOVA test with age as covariate. These values were used as well in the correlation analysis with cognitive and structural scores.

This methodology was employed as detailed for the assessing of strength differences. In our analysis, we wanted to know whether the overall strength differences (i.e. the global FC of the cluster with the rest of the brain) was caused by the existence of a few isolated strong connections rather than by a "global/widespread" effect (widespread – possibly weak – connections of each original cluster to the rest of the brain). Therefore, we focused on the mass center of the cluster and computed the FC of each node of the grid with the cluster's mass center. Using this new FC data set, we repeated the methodology of the points 2 to 4, to detect possible (secondary) clusters that are significantly connected with the original cluster of the primary analysis. Such finding would imply that the global connectivity of the primary cluster was mostly driven by connection with these secondary clusters. We decided to consider only those clusters that were not adjacent to the original one to prevent degenerate cases were the primary and secondary clusters may join if weaker cluster defining thresholds were used. they could be driven by the arbitrary selection of the cluster configuration p value.

Finally, we provide an overview of the statistical pipeline in the new Figure 2.

(9) Page 8, lines 3-5. The secondary-1 cluster included also contralateral areas (on the left): for this reason adding "mainly" is more accurate, in order to underline how the localization on the right showed a higher contribution but it is not the only one. Similarly, for the secondary-2 cluster, the fact that the involvement of the precuneus area was bilateral should be added.

We have followed the reviewer suggestion in the manuscript:

"involved mainly ipsilateral frontal and medial regions (Figure 3B & Table 2, column 2), whereas the second (referred to as "secondary-2") was found in the upper precuneus area (bilateral) (Figure 3C & Table 2, column 3)."

(10) In the discussion (and partially also in the Introduction) the authors should spell out the respect in which this study adds further indications for better understanding memory decline in the early stage of AD and also when the MCI patients convert in AD. It would be also important to discuss why in the patients that showed clinical evidence of a cognitive decline, even if mild, the authors did not find any association between FC data and the neuropsychological scores.

This is a great suggestion. We have revised the manuscript to discuss these topics:

"It is important to discuss whether the reduction of functional connectivity in the group of MCI EA+ patients was caused by the epileptiform activity epileptiform activity itself or to the malfunction of the memory networks. In epilepsy, electrophysiological data tend to show an increased high gamma frequency (30-100Hz) associated with epileptiform activity (Ren et al., 2015; von Ellenrieder et al., 2016; Rampp et al., 2021). This seems to be counterintuitive with our findings assessing MCI patients in the current study. However, there are some critical differences between those studies and ours. Whilst the cited papers assessed epileptic patients, the presence of epileptiform activity in our MCI EA+ patients were scarce (patients did not have seizures), indicating that EA itself does not sustain the network malfunction. This fact is important because the neuropathological findings in epilepsy may differ from those typically found along the AD continuum. Our data consisted of non-invasive MEG recordings, whilst (Ren et al., 2015; Rampp et al., 2021) were carried out using intracranial EEG. The results from these two methodologies are difficult to interpret together since the local effects found with intracranial EEG are quite different to the macroscopic signals assessed with non-invasive EEG/MEG. Besides the differences in the data and technique, there are some methodological differences such as the different gamma band definitions. We focused on the classical gamma band (30-45 Hz) rather than the broader definitions used in the referenced studies. Given the differences in technique and methodology, our study offers new and different information in the underlying processes that occur in tandem with the development of AD neuropathology. Therefore, our findings of aberrant functional connectivity in MCI patients are more consistent with a disruption of the episodic memory networks. This disruption cannot be explained by the epileptogenic activity alone. In fact, we hypothesize that abnormal network functioning is a risk factor for EA, and hence that MCI EA+ patients might be at higher risk of conversion to dementia. EA potentially contributes to network decline."

Regarding the correlations between functional connectivity and neuropsychological test, we did not expect any specific results. The FC markers were obtained by assessing differences between groups that did not differ in neuropsychological performance nor other structural markers. The only difference between both groups was the detection of epileptiform activity. In the post hoc correlation analyses we only found some association between structural grey matter integrity markers in the MCI EA+ group. Maybe the possible relationship between FC and cognitive performance was so faint for our data to detect. This point is something that we expect to address by developing the next project.

Minor Corrections –

(1) In the manuscript, there are some words in italics that actually do not need to be emphasized.

We have corrected this issue in the revised manuscript.

(2) Page 2, lines 10-12. This sentence could be rephrased and combined with the subsequent, deleting the part "in our Madrid cohort". This part could be more flowing.

We have modified the text following the reviewer commentary.

(3) Page 2, lines 19-21. This final sentence appears to be misleading. Indeed, the findings of the work were associated to AD and not to MCI cohort:

"These functional network disruptions and changes in brain physiology and morphology in AD (maybe MCI?) have…as they may contribute to cognitive decline in MCRI and AD".

We have corrected this issue in the new version of the manuscript by deleting "in AD".

(4) Page 5, line 49. The authors reported that the mean PLV values, used in the whole-brain matrix of 1202 nodes, were obtained by averaging the epochs corresponding to one session of MEG recording. If I understood correctly, the authors should change the word "trials", which suggests more than one resting-state recording, in "epochs" or "segments".

The reviewer is right. We have changed "trials" by "epochs" through the methods section.

(5) Page 7, line 45. The authors should delete a comma: "the MCI EA+ cluster had significantly reduced normalized global connectivity..:"

Corrected.

(6) Figure 1. At line 28, the authors should insert a comma between B and C, e.g. (B, C), and delete at line 29 "in B and C", as they are already specified at the beginning.

Corrected.

(7) Table 1. Why did the authors mention only the hippocampal volumes? The caption is too long with information that could be placed below the last row of the table and others could be summarized with symbols. For example, the authors could add a symbol ($) close to the p-value and then at the end of the table write "$= Fisher's exact test" or "#=t-Test". It is not necessary to write in the caption the variables that are listened in the table, e.g. ApoE-ε4. Also, if the authors want to indicate that MMSE score, Immediate recall and so on are cognitive scores/evaluations, they should add another row inside the table with that label and the cognitive tests moved a little bit to the right.

We have modified the caption of the Table 1 as follows:

*"**Table 1.** Demographics, genetics, clinical scores, and brain volumetric information of the 63 MCI patients indicating that the two groups are similar with regard to age, ApoE 4 genotyping, cognitive status, and selected gray matter volumes. MCI: mild cognitive impairment patients. EA, +-: existence or not of epileptiform activity, $: Fisher's exact test, #: t-tests. MMSE: Mini Mental State Examination. Total grey matter and whiter matter volumes are in mm3. Volumes of anatomical structures are normalized by intracranial volume."*

We included in the table only the hippocampal values because they are the most related, based on the existing AD literature, with the memory impairment that defines the amnesic MCI diagnosis. The lack of significant differences between groups reinforces the idea that both groups were similar from a neuropsychological or structural point of view.

(8) Table 2. The caption is still to o long. For example, "%" could be put at the end of the table as "%= percentage of the ROI within the cluster". Moreover, the authors could put a symbol close to F and then specify at the end of the table: "#= sum of all F values obtained at the node level". Finally the rest of the caption should be moved at the end of the table, writing r=right and l=left avoiding to write sentences.

Corrected. Now the caption looks as follows:

"Regions of interest (ROIs) from the AAL atlas that comprise each significant cluster. %: percentage of the ROI within the cluster. #: sum of all F values obtained at the node level. ROIs were ordered based on their significance (F column). r/l=right/left. ITG: inferior temporal gyrus; FusiG: fusiform gyrus; MTG: middle temporal gyrus; PCC: posterior cingulate gyrus; Precu: precuneus; MCC: middle cingulate gyrus; MOccL: middle occipital lobe; SPG: superior parietal gyrus; IFGt: inferior frontal gyrus triangular; IFGor: inferior frontal gyrus opercular; IFGo: inferior frontal gyrus orbital; SFGo: superior frontal gyrus orbital; RO: rolandic operculum; PosCG: postcentral gyrus; TPsup: temporal pole, superior temporal gyrus; PreCG: precentral gyrus; SFo:

superior frontal gyrus orbital; MFG: middle frontal gyrus; MFGo: middle frontal gyrus orbital; Amyg: amygdala; Parahip: parahippocampus; STG: superior temporal gyrus; TPmid: temporal pole, middle temporal gyrus."

(9) Table 3. The authors should change the "gm" in "GM" according to the standard notation. Similarly, at line 4 the details about the labels should be moved at the end of the table.

Corrected

(10) Page 12, line 11. The authors should correct "This areas" in "These areas" and change "behavior" with "the" without commas.

Corrected

(11) Page 12, line 42. The authors should delete the double point at the end of the sentence.

Corrected

Editorial Office comments:

-Please supply a graphical abstract. A graphical abstract is a visual representation of the central finding or methodology of the paper. It will be displayed in the table of contents. Authors should strive to make the graphical abstract informative, interesting, visually appealing, and straightforward (no caption needed, no abbreviations except gene names). Please upload as a separate high resolution image file (tiff preferred). The technical requirements for the graphical abstracts are:

--size of the submitted image: 1200 pixels square at 300dpi

--font of the text: Arial 12-16 point (smaller fonts will not be legible online)

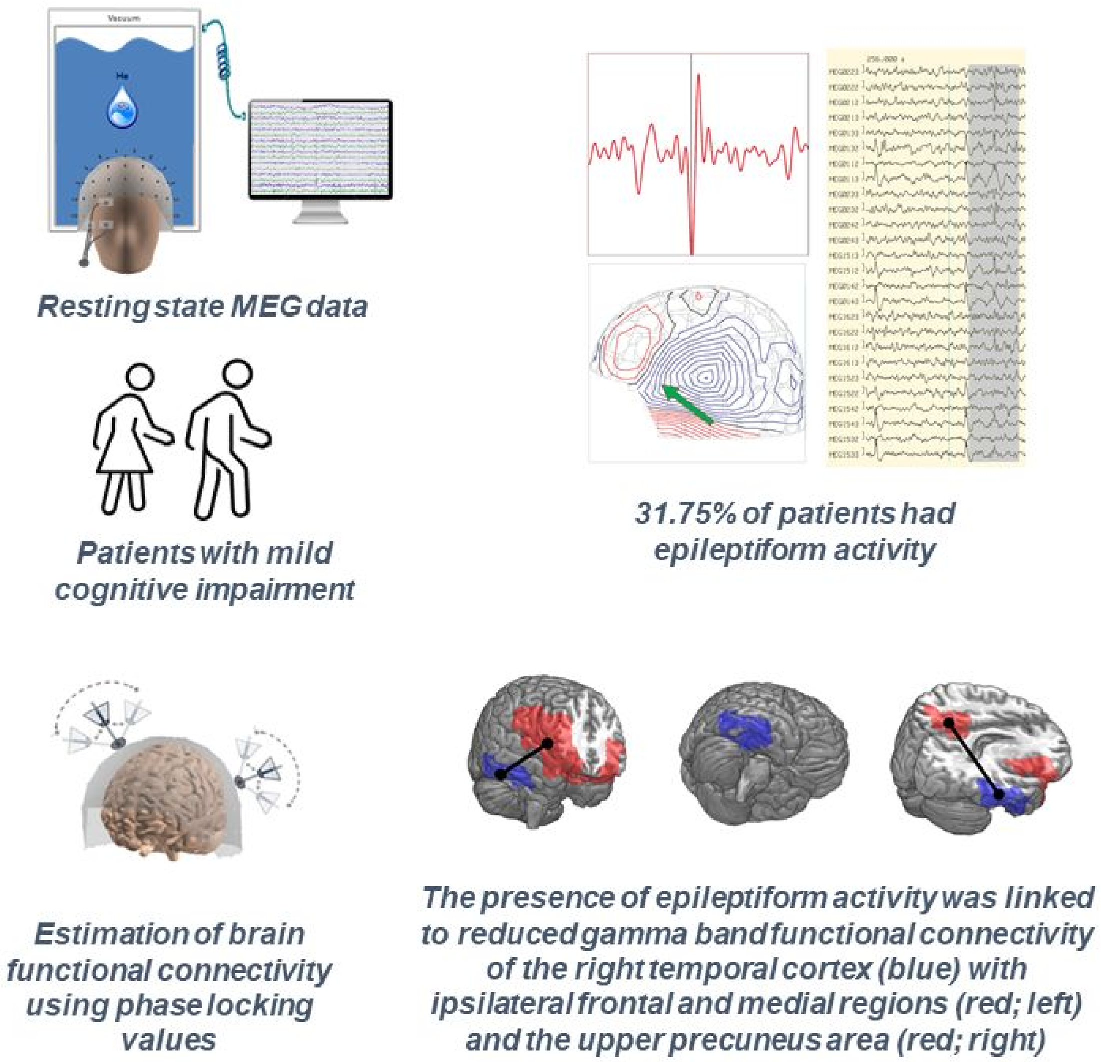

-Please add an Abbreviated Summary (up to 50 words) of your paper that captures the main purpose and conclusions of the work. The summary should state the main results written in the third person ('[1st author surname] et al. report that …') and conclusion. This summary will be used in the contents list online and should not contain any abbreviations other than those on the accepted abbreviations list and accepted gene/protein names, making it accessible to the non-specialist. Please upload it as a Word

document. Allowed abbreviations: AIDS; ANOVA; AMPA; ATP; A,T,C,G; CNS; CSF; CT; DNA; ECG; EEG; EMG; GABA; HIV; MRI; NMDA; PET; PCR; RNA.

We prepared the following abbreviated summary, also uploaded as a Word document:

Cuesta et al. report that the presence of epileptiform activity in magnetoencephalography data of patients with mild cognitive impairment is linked to reduced gamma band functional connectivity of the right temporal cortex with the rest of the brain. This reduced connectivity was associated with gray matter atrophy across several cortical regions.

- To comply with our ethical standards, the appropriate checklist must be included (see Reporting Guideline section within 'Instructions to authors').

We include the checklist of STROBE.

-Please include a paragraph about data availability in the 'Materials and Methods' section. We strongly encourage data sharing either as supplementary information or uploads to repositories which you can link in the paper.

A new paragraph has been added at the end of the methods section.

"Data availability

The data that support the findings of this study are available from the corresponding author, upon request. All the algorithms used in the present paper are reported in the 'Materials and methods' section."

-Please, include details of the statistical analysis including relevant test statistic values alongside p values.

In response to reviewers, we now describe the statistical procedures, and especially the cluster-size permutation test, in much more detail. Further, for the cluster-based permutation test results, we included in the Table 2 the F-statistic values (cluster mass statistic) at the node level corresponding with each AAL ROI region captured by the significant clusters. Additionally, we have included in the new version of the manuscript the information about the whole cluster as follows:

"We found one significant cluster (CBPT; cluster mass statistic = 268.14; p-value = 0.0160) in the gamma band"

Finally, as a unique representative score for the between groups differences that each significant cluster captured, we have displayed the post hoc ANCOVA test (p value and F statistic).

"We computed the average strength of the nodes contained in the cluster as a surrogate effect size and carried out a new ANCOVA Test with age to quantify it. The values obtained of the differences at the cluster level were p value < 0.001 and F-statistic 19.4".

"The average strength of the FC between the primary cluster and the secondary-1 differed between groups with the following scores p value < 0.001 and F-statistic 25.6. The second (referred to as "secondary-2") was found in the upper precuneus area (bilateral) (Figure 3C & Table 2, column 3). In this case, the differences between groups for the average strength of the FC between the primary cluster and the secondary-2 showed a p value < 0.001 and F-statistic 15.5".

References

Bagić, A. I. *et al.* (2011) 'American clinical magnetoencephalography society clinical practice guideline 1: Recording and analysis of spontaneous cerebral activity', *Journal of Clinical Neurophysiology*, 28(4), pp. 348–354. doi: 10.1097/WNP.0b013e3182272fed.

von Ellenrieder, N. *et al.* (2016) 'Detection and Magnetic Source Imaging of Fast Oscillations (40–160 Hz) Recorded with Magnetoencephalography in Focal Epilepsy Patients', *Brain Topography*, 29(2), pp. 218–231. doi: 10.1007/s10548-016-0471-9.

Fernandes, J. M. *et al.* (2005) 'What does an epileptiform spike look like in MEG? Comparison between coincident EEG and MEG spikes', *J. Clin. Neurophysiol.* Department of Electronics and Telecommunications and IEETA, University of Aveiro, Portugal. jfernan@ieeta.pt, 22(1), pp. 68–73. doi: 10.1097/01.wnp.0000150999.67749.6d.

Jack, C. R. and Holtzman, D. M. (2013) 'Biomarker modeling of alzheimer's disease', *Neuron*. Elsevier Inc., 80(6), pp. 1347–1358. doi: 10.1016/j.neuron.2013.12.003.

Maestú, F. *et al.* (2015) 'A multicenter study of the early detection of synaptic dysfunction in Mild Cognitive Impairment using Magnetoencephalography-derived functional connectivity.', *NeuroImage. Clinical*, 9, pp. 103–9. doi: 10.1016/j.nicl.2015.07.011.

Maris, E. and Oostenveld, R. (2007) 'Nonparametric statistical testing of EEG- and MEG-data', *J. Neurosci. Methods*, 164(1), pp. 177–190. doi: 10.1016/j.jneumeth.2007.03.024.

Nakamura, A. *et al.* (2017) 'Early functional network alterations in asymptomatic elders at risk for Alzheimer's disease', *Scientific Reports*. Springer Nature, 7(1). doi: 10.1038/s41598-017-06876-8.

Nakamura, A. *et al.* (2018a) 'Electromagnetic signatures of the preclinical and prodromal stages of Alzheimer's disease', *Brain*. doi: 10.1093/brain/awy044.

Nakamura, A. *et al.* (2018b) 'Electromagnetic signatures of the preclinical and prodromal stages of Alzheimer's disease', *Brain*, 141(5), pp. 1470–1485. doi: 10.1093/brain/awy044.

Nowak, R., Santiuste, M. and Russi, A. (2009) 'Toward a definition of MEG spike: parametric description of spikes recorded simultaneously by MEG and depth electrodes', *Seizure*. Magnetoencephalography Unit, Centro Medico Teknon, Vilana 12, 08022 Barcelona, Spain. rnowak@catalonia.net, 18(9), pp. 652–655. doi: 10.1016/j.seizure.2009.07.002.

Oostenveld, R. *et al.* (2011) 'FieldTrip: Open source software for advanced analysis of MEG, EEG, and invasive electrophysiological data.', *Computational intelligence and neuroscience*, 2011, p. 156869. doi: 10.1155/2011/156869.

Rampp, S. *et al.* (2021) 'Dysmorphic neurons as cellular source for phase-amplitude coupling in Focal Cortical Dysplasia Type II', *Clinical Neurophysiology*. International Federation of Clinical Neurophysiology, 132(3), pp. 782–792. doi: 10.1016/j.clinph.2021.01.004.

Ren, L. *et al.* (2015) 'Gamma oscillations precede interictal epileptiform spikes in the seizure onset zone', *Neurology*, 84(6), pp. 602–608. doi: 10.1212/WNL.0000000000001234.

Ung, H. *et al.* (2017) 'Interictal epileptiform activity outside the seizure onset zone impacts cognition', *Brain*, 140(8), pp. 2157–2168. doi: 10.1093/brain/awx143.

Zalesky, A., Fornito, A. and Bullmore, E. T. (2010) 'Network-based statistic: Identifying differences in brain networks', *NeuroImage*. Elsevier Inc., 53(4), pp. 1197–1207. doi: 10.1016/j.neuroimage.2010.06.041.
