# Supplementary material for: Gamma band functional connectivity reduction in patients with amnestic mild cognitive impairment and epileptiform activity
Source: Brain Commun. 2022 Feb 3;4(2):fcac012. doi: 10.1093/braincomms/fcac012 (PMC8914494; doi:10.1093/braincomms/fcac012)
Supplement: fcac012_Supplementary_Data [file Original Submission GÇô Decision Letter.pdf › Original Submission GÇô Decision Letter.pdf]

Original Submission – Decision Letter

28-Apr-2021

Dear Dr. Cuesta,

Thank you for your recent submission BRAINCOM-2021-090 entitled "Gamma band functional connectivity reduction in patients with amnestic mild cognitive impairment and epileptogenic activity" to Brain Communications.

Your manuscript has been reviewed, and while the reviewers and editorial team appreciate many aspects of the paper, they raise some concerns that you will need to address in order for the paper to be considered for publication.

If you can address these concerns, I invite you to submit a revised manuscript and a response to each of the reviewers' comments. The reviewers' comments are included at the bottom of this message. In particular, you will have to address some methodological issues, and change the terminology used (both reviewers suggest that epileptiform activity is more correct).

In addition to the reviewers' comments, please note our editorial comments added below to comply with our requirements for presentation of scientific data at Brain Communications.

Please format your revised manuscript according to our Instructions to Authors https://academic.oup.com/braincomms/pages/General_Instructions – a summary of key points is attached to help with this.

To revise your manuscript, click the link below or log into https://mc.manuscriptcentral.com/braincom and enter your Author Centre, where you will find your manuscript title listed under "Manuscripts with Decisions." Under "Actions," click on "Create a Revision." Your manuscript number will be appended to denote a revision.

*** PLEASE NOTE: This is a two-step process. After clicking on the link, you will be directed to a webpage to confirm. ***

https://mc.manuscriptcentral.com/braincom?URL_MASK=ca55be388bf14e6b8c0500e19aeb6131

Please upload a revised manuscript with changes tracked with track changes or in coloured text.

When submitting your revised manuscript, you will be asked to respond to the reviewer(s) comments. Please be as specific as possible in your response to all of the comments.

The editorial team at Brain Communications aims to increase transparency in scientific publishing and to demonstrate the value that peer review brings to the scientific process. Please therefore consider whether you are willing to have reviewer(s)' comments on your paper and your response published as supplementary data with the paper if it is accepted. This is entirely optional and will only be done if both the authors and reviewers agree. While submitting your revision there will be an opportunity to review or update the answer you gave previously, if necessary.

IMPORTANT: Your original files are available to you when you upload your revised manuscript. Please delete any redundant files before completing the submission.

Because we are trying to facilitate timely publication of manuscripts submitted to Brain Communications, we would appreciate your revised manuscript as soon as possible. If it is not possible for you to resubmit within 3 months, the option to submit a revision will expire, and we would then consider your revised paper as a new submission. However, if you need a little more time, please get in touch and we can extend this deadline for you.

Once again, thank you for submitting your manuscript to Brain Communications and I look forward to receiving your revision.

Yours sincerely,

Dr. Alberto Lleo
Editor, Brain Communications

Reviewer(s)' Comments to Author:

Reviewer: 1

Comments to the Author
The authors study differences in network connectivity in patients with MCI with and without activity reminiscent of interictal epileptic discharges (EA). They report gamma-band disruption in patients with EA in right temporal-frontal/medial networks, which were found to be related to gray matter atrophy. The study provides evidence on a potential link between MCI/Alzheimer's disease and epileptic activity, a highly relevant topic both scientifically and clinically. The applied methodology is state of the art and the results largely support the conclusions. I still have a number of questions, comments and suggestions.
- I'm not sure if the term „epileptogenic" activity is adequate in the presented context. In contrast to the more neutral (in my opinion at least) "epileptic" or "epileptiform" activity, "epileptogenic" implies a link to generation of epilepsy. While this could be true, I'd believe that this connotation might be too hypothetical at the moment. Then again, this might overinterpret the term.
- Not necessarily contradicting the authors' statement on decreased gamma activity with the appearance of epileptic discharges in an animal model of AD, gamma increases in the vicinity of epileptic foci have also been demonstrated (e.g. (Ren et al., 2015; von Ellenrieder et al., 2016; Rampp et al., 2021)), which seem to be associated with the epilepsy, complementing the mentioned findings on gamma changes during cognitive tasks.
- Subjects: I assume the subjects were recruited prospectively and represent a consecutive series?
- What is the reason for the T1/T2 MRI within 54 weeks before the MEG inclusion criterion? Fr MEG analysis, new T1 were acquired, as I understand?
- Recording time: 5 minutes of recording, while potentially adequate of investigating connectivity changes in relation to MCI, would be rather short to capture interictal epileptic discharges at least in a patient with focal epilepsy. Potentially, this would lead to an underestimation of EA occurrence in the study. However, this would likely decrease the contrast between groups, suggesting that if this is a problem, the results would potentially also underestimate the observed effects.
- Minor issue: numbers in table 1 should use dots instead of commas
- Did you investigate the localizations of the EA? Were they consistent and/or did they localize to the MCI EA+ cluster? If so, one explanation for the lower connectivity in the gamma band could be due to the local gamma being generated or associated with the EA patterns themselves, which would show local increases with limited synchronization with distant areas. Furthermore, filter ringing due to two-pass filtering of sharp EA patterns with a high order filter (although FIR filters were used) could induce "false" gamma, which would probably also show only local increases.
- Were correlation analyses corrected for multiple comparisons?
- Minor issue: p. 12 line 12: "these" instead of "this" and following sentence "have been associated"
- The lack of group differences or correlations to behavioral variables/cognitive performance is somewhat puzzling. Would you believe that this is due to intact compensation mechanisms? Could more detailed neuropsychological evaluation yield differences? Or is this due to the (shortly mentioned) fact that only resting-state activity was evaluated? This aspect should be discussed.
- The results show interesting parallels to the finding of transient cognitive impairment (TCI) in patients with epilepsy (e.g. (Ung et al., 2017)), which suggest that epileptic activity itself may interfere with cognitive processes. Since the spatial distribution of epileptic activity seems to play a role in this phenomenon, it would be interesting to also describe and discuss the EA localization in relation to the observed findings.
- On the bottom line, would you think that EA is actively contributing to cognitive decline? Or is this "only" an epiphenomenon of network disruption, but a potentially valuable indicator nonetheless?

References
von Ellenrieder N, Pellegrino G, Hedrich T, Gotman J, Lina J-M, Grova C, et al. Detection and Magnetic Source Imaging of Fast Oscillations (40–160 Hz) Recorded with Magnetoencephalography in Focal Epilepsy Patients. Brain Topogr. 2016; 29: 218–231.
Rampp S, Rössler K, Hamer H, Illek M, Buchfelder M, Doerfler A, et al. Dysmorphic neurons as cellular source for phase-amplitude coupling in Focal Cortical Dysplasia Type II. Clin. Neurophysiol. 2021; 132: 782–792.
Ren L, Kucewicz MT, Cimbalnik J, Matsumoto JY, Brinkmann BH, Hu W, et al. Gamma oscillations precede interictal epileptiform spikes in the seizure onset zone. Neurology 2015; 84: 602–608.
Ung H, Cazares C, Nanivadekar A, Kini L, Wagenaar J, Becker D, et al. Interictal epileptiform activity outside the seizure onset zone impacts cognition. Brain 2017; 140: 2157–2168.

Reviewer: 2

Comments to the Author
The study submitted by Cuesta et al. presents an interesting analysis of the gamma band functional connectivity by means of MEG recordings in patients with MCI and epileptogenic activity (EA). The authors enrolled a consistent number of MCI subjects with detailed inclusion criteria. For each patient

they acquired: (i) T1-weighted MRI images for the volumetric analysis and (ii) MEG recordings in resting-state condition used for the subsequent source reconstruction and connectivity analysis. The data were analyzed with respect to the presence or the absence of the EA in the MCI patients, investigating possible functional connectivity alterations in specific frequency bands. The authors have also studied the sources of the connectivity analysis and the correlation with the neuropsychological and volumetric measures of patients. Overall the manuscript is well written with some sections that may need further explanations. Specific comments are reported below.

Major Concerns –

(1) The term of "epileptogenic activity" means that there is certainly an epileptic activity. If I understand correctly, the patients did not have a diagnosis of epilepsy but some EEG alterations resembling epilepsy were detected. In this case, it might be more accurate to use the term "epileptiform activity".

(2) Page 4, line19. In case the two independent experienced radiologists did not agree, how did the authors decide? Did they request a third opinion to another experienced radiologist?

(3) Page 5, line 8. In the artifact-free data, further analyzed, did the authors find any signs of the epileptiform activity? Did an expert in epilepsy check the epochs in order to identify possible spikes? The presence of spikes should affect the analysis, especially in terms of frequency bands. In this case, I think the authors should discuss their findings with respect to their presence.

(4) Page 5, line 23. The approach used to identify the epileptiform activity is quite clear. However, I do not understand if the authors have selected the patients with an analysis of their electrical activity or with the localization of the dipoles. This aspect should be clarified and in case of the dipoles, the authors should explain this choice. Moreover, it might be interesting to specify how many spikes were found in each patient, in order to better describe the epileptiform activity, which is the crucial factor used to divided the investigated clinical cohort. Finally, if the MEG expert and neurologist trained in MEG reading did not agree, how did the authors decided? Did they request a third opinion to another experienced neurologist?

(5) Page 5, lines 30-54. I think that in order to explain more clearly the procedure used for the connectivity analysis, it might be useful to present a figure with the sequences followed by the authors. For example, a brain image related to the 78 cortical areas of AAL with superimposed nodes. Then, a schematic representation of the whole-brain matrix of the 1202 nodes with the formula of PLV etc. to conclude with the brain map the authors obtained after the normalization of the node strengths, specifying that this approach was applied for each patient and frequency band.

(6) Page 6, lines 15-18. In the manuscript, for each patient and for each frequency band, a brain map with normalized node strengths (1202 nodes) has been computed. After that, ANCOVA has been applied in order to identify the differences of strength's nodes between the experimental groups. This procedure gave another brain map composed by 1202 F-values regarding the comparisons of each node between the two clinical cohorts (line13). A threshold of p-value=0.005 was used to select the nodes which differs significantly among the two group of patients. This brain map, composed by suprathreshold F-values, was then additionally split in two: one with nodes that showed positive differences and another one with nodes with negative differences. At this point, the analysis was still at a node level. Then, the cluster level has been introduced. A cluster has to be composed by at least 12 nodes. How did the authors cluster the nodes? For example, can they identify a criterion such as the one discussed at line 32 and 33 for the seeds? Moreover, how many clusters did the authors find in the positive and negative F-values maps? In the followed statistical procedure, did the authors include clusters from both positive and negative F-values map? It might be helpful to show in a figure the procedure with which the authors extracted the clusters, perhaps together with the previous one in a different panel (e.g. Fig.1b). In addition, I think that some part of the presentation should be rewritten in order to make the statistical analysis section more comprehensible.

(7) Page 6, line 33. Why did the authors think that comparing each node of the grid with the seed-cluster regions could provide information about a possible isolated, local contribution of few nodes to FC differences between groups? Which kind of statistical analysis did the authors apply to evaluate the existence of group differences in the FC in terms of seeds? Could the authors explain the sentence at line 37? Why did they decide to take in account only the clusters that did not overlap with the original seed-cluster?

(8) Page 6, line 43. This sentence should be clarified.

(9) Page 8, lines 3-5. The secondary-1 cluster included also contralateral areas (on the left): for this reason adding "mainly" is more accurate, in order to underline how the localization on the right showed a higher contribution but it is not the only one. Similarly, for the secondary-2 cluster, the fact that the

involvement of the precuneus area was bilateral should be added.

(10) In the discussion (and partially also in the Introduction) the authors should spell out the respect in which this study adds further indications for better understanding memory decline in the early stage of AD and also when the MCI patients convert in AD. It would be also important to discuss why in the patients that showed clinical evidence of a cognitive decline, even if mild, the authors did not find any association between FC data and the neuropsychological scores.

Minor Corrections –
(1) In the manuscript, there are some words in italics that actually do not need to be emphasized.

(2) Page 2, lines 10-12. This sentence could be rephrased and combined with the subsequent, deleting the part "in our Madrid cohort". This part could be more flowing.

(3) Page 2, lines 19-21. This final sentence appears to be misleading. Indeed, the findings of the work were associated to AD and not to MCI cohort:
"These functional network disruptions and changes in brain physiology and morphology in AD (maybe MCI?) have…as they may contribute to cognitive decline in MCRI and AD".

(4) Page 5, line 49. The authors reported that the mean PLV values, used in the whole-brain matrix of 1202 nodes, were obtained by averaging the epochs corresponding to one session of MEG recording. If I understood correctly, the authors should change the word "trials", which suggests more than one resting-state recording, in "epochs" or "segments".

(5) Page 7, line 45. The authors should delete a comma: "the MCI EA+ cluster had significantly reduced normalized global connectivity..:"

(6) Figure 1. At line 28, the authors should insert a comma between B and C, e.g. (B, C), and delete at line 29 "in B and C", as they are already specified at the beginning.

(7) Table 1. Why did the authors mention only the hippocampal volumes? The caption is too long with information that could be placed below the last row of the table and others could be summarized with symbols. For example, the authors could add a symbol ($) close to the p-value and then at the end of the table write "$= Fisher's exact test" or "#=t-Test". It is not necessary to write in the caption the variables that are listened in the table, e.g. ApoE-ε4. Also, if the authors want to indicate that MMSE score, Immediate recall and so on are cognitive scores/evaluations, they should add another row inside the table with that label and the cognitive tests moved a little bit to the right.

(8) Table 2. The caption is still to o long. For example, "%" could be put at the end of the table as "%= percentage of the ROI within the cluster". Moreover, the authors could put a symbol close to F and then specify at the end of the table: "#= sum of all F values obtained at the node level". Finally the rest of the caption should be moved at the end of the table, writing r=right and l=left avoiding to write sentences.

(9) Table 3. The authors should change the "gm" in "GM" according to the standard notation. Similarly, at line 4 the details about the labels should be moved at the end of the table.

(10) Page 12, line 11. The authors should correct "This areas" in "These areas" and change "behavior" with "the" without commas.

(11) Page 12, line 42. The authors should delete the double point at the end of the sentence.

Editorial Office comments:

-Please supply a graphical abstract. A graphical abstract is a visual representation of the central finding or methodology of the paper. It will be displayed in the table of contents. Authors should strive to make the graphical abstract informative, interesting, visually appealing, and straightforward (no caption needed, no abbreviations except gene names). Please upload as a separate high resolution image file (tiff preferred). The technical requirements for the graphical abstracts are:
--size of the submitted image: 1200 pixels square at 300dpi
--font of the text: Arial 12-16 point (smaller fonts will not be legible online)

-Please add an Abbreviated Summary (up to 50 words) of your paper that captures the main purpose

and conclusions of the work. The summary should state the main results written in the third person ('[1st author surname] et al. report that …') and conclusion. This summary will be used in the contents list online and should not contain any abbreviations other than those on the accepted abbreviations list and accepted gene/protein names, making it accessible to the non-specialist. Please upload it as a Word document. Allowed abbreviations: AIDS; ANOVA; AMPA; ATP; A,T,C,G; CNS; CSF; CT; DNA; ECG; EEG; EMG; GABA; HIV; MRI; NMDA; PET; PCR; RNA.

- To comply with our ethical standards, the appropriate checklist must be included (see Reporting Guideline section within 'Instructions to authors').

-Please include a paragraph about data availability in the 'Materials and Methods' section. We strongly encourage data sharing either as supplementary information or uploads to repositories which you can link in the paper.

-Please, include details of the statistical analysis including relevant test statistic values alongside p values.
