# Supplementary material for: Gamma band functional connectivity reduction in patients with amnestic mild cognitive impairment and epileptiform activity
Source: Brain Commun. 2022 Feb 3;4(2):fcac012. doi: 10.1093/braincomms/fcac012 (PMC8914494; doi:10.1093/braincomms/fcac012)
Supplement: fcac012_Supplementary_Data [file Revision 1 -Author's Response.pdf › Revision 1 -Author's Response.pdf]

November 29, 2021

Dr. Alberto Lleo
Editor,
Brain Communications

Manuscript #BRAINCOM-2021-090

Dear Dr. Alberto Lleo,

We attach our revised manuscript, entitled "***Gamma band functional connectivity reduction in patients with amnestic mild cognitive impairment and epileptogenic activity,***" to be considered for publication in Brain Communications.

We would like to thank the editor and reviewer for their constructive comments. We have carefully revised our manuscript and have responded to the individual points raised by the editor and the reviewer 2. We hope the manuscript is now suitable for publication.

This re-submission includes the revised manuscript with revisions highlighted in red. In addition, we have created a new table displaying the epileptiform activity of each patient following the reviewers' suggestion.

We look forward to hearing from you.

Sincerely,

On behalf of the co-authors,
Pablo Cuesta
Laboratory of Cognitive and Computational
Neuroscience Lab. UCM, Madrid, Spain.

Reviewer 2.

Comments to the Author
The authors have addressed to a sufficient extent the issues I raised in my previous report and the new figures facilitate the comprehension of the methods, making the manuscript more readable. However, as the authors based their study on how the hyper-excitability in MCI patient might induce alterations of crucial frequency bands associated to memory formation, in my opinion a more detailed description of MCI EA+ groups should be made available.

For example, a Supplemental Table that shows for each patient the number of spikes, where they are detected (e.g., left T) and the normalized global FC for primary, secondary-1 and secondary-2 networks.

We appreciate the reviewer's comments on this. The existence of epileptiform activity (EA) in mild cognitive impairment (MCI) patients is key in developing this work. Thus, we have created a supplementary material section that includes two new tables. "Table Supp. 1" depicts the EA information of each patient.

Patient	Location (number of spikes)
1	Left midtemporal superior gyrus (2)
2	Right mesial temporal, basal temporal, and posterior temporal (4)
3	Left=Right mid superior temporal sulcus (2)
4	Right mesial temporal (1)
5	Left frontal opercular (1)
6	Right basal temporal (1)
7	Right anterior temporal (2)
8	Left posterior temporal (1)
9	Right anterior temporal (1), Right frontal opercular (1)
10	Right peri-Sylvian, inferior frontal gyrus (2)
11	Left anterior and mid temporal temporal (4)
12	Right insular (intra-sylvian) (1)
13	Left mesial temporal (1)
14	Right inferior frontal gyrus (1)
15	Left frontal opercular (1)
16	Right frontal opercular (1)
17	Right frontal paracentral dorsolateral (1)
18	Bilateral frontal opercular L>>R (9)
19	Right mid temporal (1)
20	Right basal temporal, Right parietal, right insular (3)

Table Supp. 1. Description of the epileptiform activity found for each MCI (EA+) patient. **L**, left. **R**, right, number of discharges in brackets; laterality is indicated by = (symmetric) and >> for dominance of one hemisphere. The temporal lobe is presented in several sub-regions: mesial, basal, anterior temporal, mid temporal, posterior temporal. Frontal lobe sources are presented in sub-regions: inferior frontal, fronto-opercular, mesial frontal, dorsolateral,

Regarding the second suggestion of the reviewer (i.e., a table with the normalized global FC for primary, secondary-1 and secondary-2 networks), we had indirectly displayed this info in the boxplots of Figure 3. Since the information displayed in this figure is more qualitative than quantitative, we have created a new table that has been added to the new supplementary material section as "Table Supp. 2"

**Patient**	Primary cluster	Secondary-1	Secondary-2
1	0,1705	0,1992	0,1492
2	0,1648	0,1736	0,1600
3	0,1613	0,1737	0,1550
4	0,1617	0,1817	0,1374
5	0,1814	0,1914	0,1683
6	0,1725	0,1891	0,1429
7	0,1713	0,1977	0,1402
8	0,1694	0,1914	0,1409
9	0,1692	0,1797	0,1472
10	0,1687	0,1809	0,1460
11	0,1685	0,1853	0,1640
12	0,1549	0,1644	0,1334
13	0,1763	0,1910	0,1565
14	0,1618	0,1828	0,1418
15	0,1742	0,1975	0,1365
16	0,1718	0,1841	0,1370
17	0,1685	0,1769	0,1584
18	0,1649	0,1917	0,1358
19	0,1687	0,1765	0,1554
20	0,1673	0,1856	0,1517

Table Supp. 2. MCI-EA+ individual normalized functional connectivity of each significant marker of the manuscript. Markers are displayed in Figure 3 of the manuscript.

It might be also interesting to discuss the large percentage of MCI patients with EA (31%) described in the paper with respect to the incidence reported in the literature, ranging from none (routine EEG) to around 12% (long-term monitoring EEG).

We agree that this is an interesting point. In response, we believe that this apparent disagreement is due the heterogeneity of the studies and populations. The relationship between dementia and epilepsy is well described (Vossel *et al.*, 2017). As can be seen in this review, seizures occur in around 10–22% of patients with Alzheimer's disease, but the literature reports incidences as high as 64%. In this study, the patient cohort was designed to study the prodromal stages of Alzheimer's Disease, rather than to assess the existence of subclinical EA. Carefully evaluating the available ~20 minutes of data per each patient, we found that, indeed, 31% of the MCI patients showed at least one spike. It is important to note that the EA described in this study is in all cases subclinical, scarce (2.04 ± 1.79 per MCI EA+ patient), and without a clear association with an epileptogenic region. This pattern indicated that the EA incidence in our sample was low. Nevertheless, we do not believe our numbers diverge too far from previous studies. In Horvath *et al.* (2019), the authors described epileptic seizures in 26% of mild AD patients. In a recent study (Babiloni *et al.*, 2020), the authors found that 41% of patients with amnesic mild cognitive impairment not due to Alzheimer's disease displayed some EA. This cipher was very similar to the one depicted in Vossel *et al.* (2016), where 42% of AD patient showed

subclinical EA (as did 10% of the controls).

One factor that could be driving the diversity of incidences of EA is the existence of comorbid cerebrovascular disease, since it may be the underlying cause of about 50-70% of new-onset epilepsy in the elderly (Sen, Capelli and Husain, 2018). Our sample was recruited with an exclusion criterion of not having vascular dementia. However, subclinical damage cannot be avoided. In fact, we are now running a study to assess the influence of vascular damage in the functional architecture of the brain. We will be able to evaluate whether the MCI-EA+ patients have higher vascular damage than those without EA. Our sample was recruited with an exclusion criterion of not having vascular dementia. However, subclinical damage cannot be avoided.

An additional, and often underestimated contributing factor is the different sensitivity of MEG and EEG for epileptic discharges. Since the advent of clinical MEG we know that there are EEG unique discharges as well es MEG unique discharges (Park et al., 2004; Iwasaki et al., 2005; Ebersole et al., 2018; Laohathai et al., 2021). MEG and EEG are very complimentary modalities. In MEG recordings without EEG one could not determine the EEG only spike contribution. In such data set it also would be difficult to reliably exclude the most common MEG unique normal variants; those usually have no EEG correlates. In an EEG data set without MEG one will miss the MEG unique discharges. In our available preliminary data set we would capture common M/EEG discharges, MEG unique discharges, and MEG unique normal variants. The latter one might contribute to an overestimation of EA in our sample. On the other hand, the lack of simultaneously recorded EEG leads to an underestimation of EA because we cannot capture unique EEG discharges. Consequently, we are now running a study to assess the influence of vascular damage in the functional architecture of the brain, and we record simultaneous M/EEG in all subjects, as per the existing clinical practice guidelines for MEG (Bagić *et al.*, 2011). We will be able to evaluate whether the MCI-EA+ patients have higher vascular damage than those without EA.

Moreover, the authors should remove the list of abbreviation currently in the caption of the tables and put it at the end of the table itself, just below the last line of data, as it is usually done in most scientific papers.

This issue has been corrected in the new version of the manuscript.

Finally, I really appreciated the discussion the authors do of the limitations of the detection of EA in their clinical cohort of MCI patients. However, this limitation might cause not only an underestimation of EA detection in MCI EA+ groups but also in the MCI EA- group. I think this point should be discussed more thoroughly.

We have modified the paragraph in the limitations of the discussion to clarify the point raised by the reviewer:

"Longer and more epilepsy-specific recordings could have allowed both the detection of more MCI EA+ patients (with the consequent decrease in the number of MCI EA-), and a better characterization of the EA in the MCI EA+ patients."

Additional comments on the revised manuscript are.
Page 13, line 52 the authors state that: EA in patients with AD (including patients with

MCI) has been associated with a faster decline in global cognition and executive functions (Vossel KA et al., 2016) as well as with a higher percentage of conversion from MCI to dementia (Vossel et al., 2017). Vossel KA et al. (2016) actually analyzed MCI with epilepsy but not with only EA because they did not detect this group (no epilepsy in the presence of EA) in their large cohort (233 MCI patients). Accordingly, the comment in parenthesis, highlighted in bold above, should be removed because it is not completely correct or, at least, it should be corrected as: (including patients with MCI and epilepsy).

This issue has been corrected in the new version of the manuscript. In addition we have included two new references here (Sen, Capelli and Husain, 2018; Nardi Cesarini *et al.*, 2020). Now the section highlighted by the reviewer looks as follows:

"EA in patients with AD (including patients with MCI and epilepsy) has been associated with a faster decline in global cognition and executive functions (Vossel KA *et al.*, 2016; Sen, Capelli and Husain, 2018; Baker *et al.*, 2019) *as well as with a higher percentage of conversion from MCI to dementia (Vossel et al., 2017)"*

Page 14, line 47-49 the authors state that: It is possible, then that this gamma band network disruption reflects the episodic memory dysfunction rather than the EA itself. Nonetheless, it appears that EA is associated with the disruption of this network. The authors aimed at studying how the hyper-excitability in MCI patient might induce alterations of crucial frequency bands associated to memory formation. However, with the addition of this new part in the discussion, the message seems to be quite different: the hyper-excitability identified in the EA does not alter, or at least not as the main factor, the functioning of the analyzed network. Although the last sentences conveys the idea that at the end the hyper-excitability is associated with disruption of this network, the final results is actually confusing. I reckon to be very important to clarify this crucial point, because these two experimental groups did not differ for their cognitive performance and therefore most probably neither they did for the functioning of the network. Indeed, they are all categorized as MCI patients and the main aspect that differentiates these two groups is the presence or the absence of EA.

We appreciate the reviewer's insight regarding that specific paragraph in the discussion. It is true that with the data presented, we cannot state mechanistic inferences between dementia-epilepsy-functional integrity. Following the suggestion of the reviewer, we have dismissed the whole paragraph and reinforced the idea of EA being an early sign of consequent network disruption. To strengthen this idea, we have included in the previous paragraph a new reference where a longitudinal assessment showed that patients with EA suffered faster cognitive decline. The patients in that study had AD, but we believe that the presence of EA in our sample is reflecting the same underlying process. On the other hand, we postulate that our gamma FC signature is another early marker that can serve as a proxy for network disruption in the making. Nevertheless, epilepsy and dementia seem to be associated through a bidirectional relationship; the presence of epilepsy might enhance the risk of cognitive dysfunction and vice versa. Thus, this paper is aiming to offer valuable information in an important research field that has potential utility for patients with dementia and epilepsy.

The discussion has been modified as follows:

"This assumption is in line with (Baker et al., 2019) where the authors performed a longitudinal

cognitive assessment of AD patients with and without a seizure history. At baseline, patients did not differ but after 1 year, the AD patients with a seizure history had faster cognitive decline. This suggests that EA potentially contributes to network decline."

Throughout the manuscript, especially in the new added parts, there are some repetitions and incomplete pieces of information.

We have reviewed the whole manuscript to correct the appearance of repetitions and incomplete pieces of information.

Comments from the Scientific Editor.

-Please supply a graphical abstract. A graphical abstract is a visual representation of the central finding or methodology of the paper. It will be displayed in the table of contents. Authors should strive to make the graphical abstract informative, interesting, visually appealing, and straightforward (no caption needed, no abbreviations except gene names). Please upload as a separate high resolution image file (tiff preferred). The technical requirements for the graphical abstracts are:

--size of the submitted image: 1200 pixels square at 300dpi

--font of the text: Arial 12-16 point (smaller fonts will not be legible online)

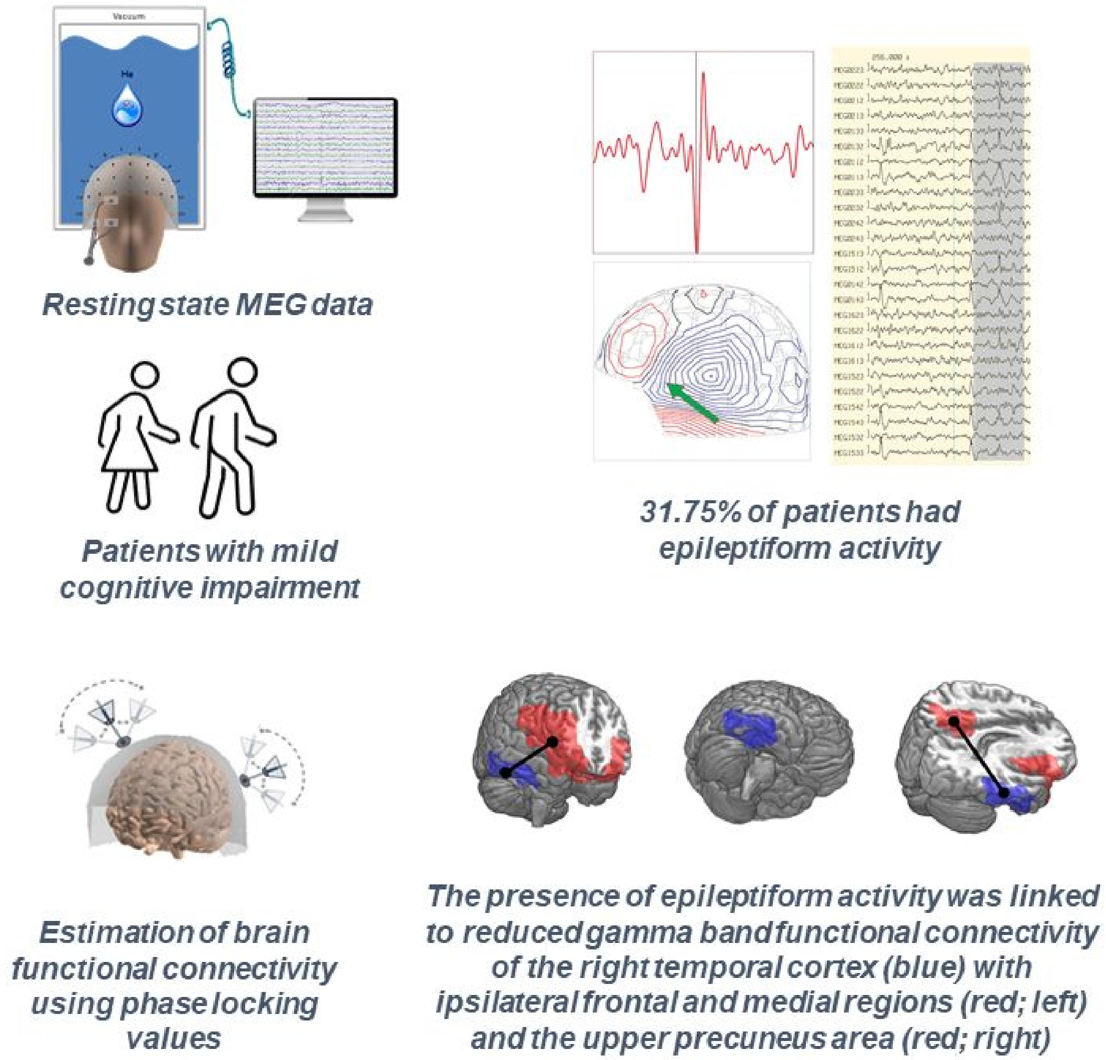

- To comply with our ethical standards, the appropriate checklist must be included (see Reporting Guideline section within 'Instructions to authors').

We include the checklist of STROBE.

REFERENCES

Babiloni, C. *et al.* (2020) ‘Abnormalities of Cortical Sources of Resting State Delta Electroencephalographic Rhythms Are Related to Epileptiform Activity in Patients With Amnesic Mild Cognitive Impairment Not Due to Alzheimer’s Disease’, *Frontiers in Neurology*, 11(October), pp. 1–20. doi: 10.3389/fneur.2020.514136.

Bagić, A. I. *et al.* (2011) ‘American clinical magnetoencephalography society clinical practice guideline 1: Recording and analysis of spontaneous cerebral activity’, *Journal of Clinical Neurophysiology*, 28(4), pp. 348–354. doi: 10.1097/WNP.0b013e3182272fed.

Baker, J. *et al.* (2019) ‘A Longitudinal Study of Epileptic Seizures in Alzheimer’s Disease’, *Frontiers in Neurology*, 10(December), pp. 1–8. doi: 10.3389/fneur.2019.01266.

Horvath, A. *et al.* (2019) ‘Precuneus-dominant degeneration of parietal lobe is at risk of epilepsy in mild alzheimer’s disease’, *Frontiers in Neurology*, 10(AUG), pp. 1–9. doi: 10.3389/fneur.2019.00878.

Nardi Cesarini, E. *et al.* (2020) ‘Late-Onset Epilepsy With Unknown Etiology: A Pilot Study on Neuropsychological Profile, Cerebrospinal Fluid Biomarkers, and Quantitative EEG Characteristics’, *Frontiers in Neurology*, 11(April), pp. 1–11. doi: 10.3389/fneur.2020.00199.

Sen, A., Capelli, V. and Husain, M. (2018) ‘Cognition and dementia in older patients with epilepsy’, *Brain*, 141(6), pp. 1592–1608. doi: 10.1093/brain/awy022.

Vossel, K. A. *et al.* (2016) ‘Incidence and impact of subclinical epileptiform activity in Alzheimer’s disease’, *Ann. Neurol.*, 80(6), pp. 858–870. doi: 10.1002/ana.24794.

Vossel, K. A. *et al.* (2017) ‘Epileptic activity in Alzheimer’s disease: causes and clinical relevance’, *Lancet Neurol.* Memory and Aging Center, Department of Neurology, University of California, San Francisco, CA, USA. Electronic address: kvossel@umn.edu. Department of Neurology, University of Toronto, ON, Canada. Division of Neurology, University of British Columbia, Van: Elsevier Ltd, 16(4), pp. 311–322. doi: 10.1016/S1474-4422(17)30044-3.

Vossel KA *et al.* (2016) ‘Incidence and Impact of Subclinical Epileptiform Activity in Alzheimer’s Disease’, *Ann Neurol.*, pp. 1–54. doi: 10.1002/ANA.24794.
