# Supplementary material for: Gamma band functional connectivity reduction in patients with amnestic mild cognitive impairment and epileptiform activity
Source: Brain Commun. 2022 Feb 3;4(2):fcac012. doi: 10.1093/braincomms/fcac012 (PMC8914494; doi:10.1093/braincomms/fcac012)
Supplement: fcac012_Supplementary_Data [file Revision 1-Decision Letter.pdf › Revision 1-Decision Letter.pdf]

Revision 1-Decision Letter

01-Sep-2021

Dear Dr. Cuesta,

Thank you for your recent submission BRAINCOM-2021-090.R1 entitled "Gamma band functional connectivity reduction in patients with amnestic mild cognitive impairment and epileptiform activity" to Brain Communications.

Your manuscript has been reviewed, and while the reviewers and editorial team appreciate many aspects of the paper, one reviewer still has some concerns that you will need to address in order for the paper to be considered for publication.

Therefore, I invite you to respond to the reviewer comments and revise your manuscript. The reviewer(s)' comments are included at the bottom of this message.

In addition to the reviewers' comments, please also address our Scientific Editor's comments added below to comply with our requirements for presentation of scientific data at Brain Communications.

Please format your revised manuscript according to our Instructions to Authors https://academic.oup.com/braincomms/pages/General_Instructions – a summary of key points is attached to help with this.

To revise your manuscript, click the link below or log into https://mc.manuscriptcentral.com/braincom and enter your Author Centre, where you will find your manuscript title listed under "Manuscripts with Decisions." Under "Actions," click on "Create a Revision." Your manuscript number will be appended to denote a new revision.

*** PLEASE NOTE: This is a two-step process. After clicking on the link, you will be directed to a webpage to confirm. ***

https://mc.manuscriptcentral.com/braincom?URL_MASK=070a3fba4c9a488bade801bfc1bfdb24

Please upload a revised manuscript with the changes you have made shown with "track changes" or in coloured text.

When submitting your revised manuscript, you will be asked to respond to the reviewer(s) comments. Please be as specific as possible in your response to all of the comments.

The editorial team at Brain Communications aims to increase transparency in scientific publishing and to demonstrate the value that peer review brings to the scientific process. Please therefore consider whether you are willing to have reviewer(s)' comments on your paper and your response published as supplementary data with the paper if it is accepted. This is entirely optional and will only be done if both the authors and reviewers agree. While submitting your revision there will be an opportunity to review or update the answer you gave previously, if necessary.

IMPORTANT: Your original files are available to you when you upload your revised manuscript. Please delete any redundant files before completing the submission.

Because we are trying to facilitate timely publication of manuscripts submitted to Brain Communications, we would appreciate your revised manuscript as soon as possible. If it is not possible for you to resubmit within 3 months, the option to submit a revision will expire, and we would then consider your revised paper as a new submission. However, if you need a little more time, please get in touch and we can extend this deadline for you.

Once again, thank you for submitting your manuscript to Brain Communications and I look forward to receiving your revision.

Best wishes

Dr. Alberto Lleo
Associate Editor, Brain Communications

Professor Tara Spires-Jones

Founding Editor, Brain Communications

Reviewer(s)' Comments to Author:

Reviewer: 1

Comments to the Author
Thank you for the detailed answers. I have no more questions and comments and recommend acceptance for publication.

Reviewer: 2

Comments to the Author
Comments to the Authors

The authors have addressed to a sufficient extent the issues I raised in my previous report and the new figures facilitate the comprehension of the methods, making the manuscript more readable. However, as the authors based their study on how the hyper-excitability in MCI patient might induce alterations of crucial frequency bands associated to memory formation, in my opinion a more detailed description of MCI EA+ groups should be made available. For example, a Supplemental Table that shows for each patient the number of spikes, where they are detected (e.g., left T) and the normalized global FC for primary, secondary-1 and secondary-2 networks. It might be also interesting to discuss the large percentage of MCI patients with EA (31%) described in the paper with respect to the incidence reported in the literature, raging from none (routine EEG) to around 12% (long-term monitoring EEG). Moreover, the authors should remove the list of abbreviation currently in the caption of the tables and put it at the end of the table itself, just below the last line of data, as it is usually done in most scientific papers. Finally, I really appreciated the discussion the authors do of the limitations of the detection of EA in their clinical cohort of MCI patients. However, this limitation might cause not only an underestimation of EA detection in MCI EA+ groups but also in the MCI EA- group. I think this point should be discussed more thoroughly. Additional comments on the revised manuscript are:

1) page 13, line 52 the authors state that: EA in patients with AD (including patients with MCI) has been associated with a faster decline in global cognition and executive functions (Vossel KA et al., 2016) as well as with a higher percentage of conversion from MCI to dementia (Vossel et al., 2017). Vossel KA et al. (2016) actually analyzed MCI with epilepsy but not with only EA because they did not detect this group (no epilepsy in the presence of EA) in their large cohort (233 MCI patients). Accordingly, the comment in parenthesis, highlighted in bold above, should be removed because it is not completely correct or, at least, it should be corrected as: (including patients with MCI and epilepsy).

2) page 14, line 47-49 the authors state that: It is possible, then that this gamma band network disruption reflects the episodic memory dysfunction rather than the EA itself. Nonetheless, it appears that EA is associated with the disruption of this network. The authors aimed at studying how the hyper-excitability in MCI patient might induce alterations of crucial frequency bands associated to memory formation. However, with the addition of this new part in the discussion, the message seems to be quite different: the hyper-excitability identified in the EA does not alter, or at least not as the main factor, the functioning of the analyzed network. Although the last sentences conveys the idea that at the end the hyper-excitability is associated with disruption of this network, the final results is actually confusing. I reckon to be very important to clarify this crucial point, because these two experimental groups did not differ for their cognitive performance and therefore most probably neither they did for the functioning of the network. Indeed, they are all categorized as MCI patients and the main aspect that differentiates these two groups is the presence or the absence of EA.

3) Throughout the manuscript, especially in the new added parts, there are some repetitions and incomplete pieces of information.

Comments from the Scientific Editor:

-Please supply a graphical abstract. A graphical abstract is a visual representation of the central finding or methodology of the paper. It will be displayed in the table of contents. Authors should strive to make the graphical abstract informative, interesting, visually appealing, and straightforward (no caption needed, no abbreviations except gene names). Please upload as a separate high resolution image file (tiff preferred). The technical requirements for the graphical abstracts are:
  --size of the submitted image: 1200 pixels square at 300dpi
  --font of the text: Arial 12-16 point (smaller fonts will not be legible online)

- To comply with our ethical standards, the appropriate checklist must be included (see Reporting Guideline section within 'Instructions to authors').
